# TRPC3/6 Channels Mediate Mechanical Pain Hypersensitivity via Enhancement of Nociceptor Excitability and of Spinal Synaptic Transmission

**DOI:** 10.1002/advs.202404342

**Published:** 2024-09-28

**Authors:** Zhi‐Chuan Sun, Wen‐Juan Han, Zhi‐Wei Dou, Na Lu, Xu Wang, Fu‐Dong Wang, Sui‐Bin Ma, Zhi‐Cheng Tian, Hang Xian, Wan‐Neng Liu, Ying‐Ying Liu, Wen‐Bin Wu, Wen‐Guang Chu, Huan Guo, Fei Wang, Hui Ding, Yuan‐Ying Liu, Hui‐Ren Tao, Marc Freichel, Lutz Birnbaumer, Zhen‐Zhen Li, Rou‐Gang Xie, Sheng‐Xi Wu, Ceng Luo

**Affiliations:** ^1^ Department of Neurobiology School of Basic Medicine Fourth Military Medical University Xi'an 710032 China; ^2^ Department of Neurosurgery Xi'an Daxing Hospital Xi'an 710016 China; ^3^ The Assisted Reproduction Center Northwest Women and Children's Hospital Xi'an 710000 China; ^4^ Department of Orthopedics Xijing Hospital Fourth Military Medical University Xi'an 710032 China; ^5^ Department of Orthopedic Surgery The University of Hong Kong‐Shenzhen Hospital Shenzhen Guangdong 518053 China; ^6^ Institute of Pharmacology Heidelberg University 69120 Heidelberg Germany; ^7^ Institute of Biomedical Research (BIOMED) Catholic University of Argentina Buenos Aires C1107AVV Argentina; ^8^ Signal Transduction Laboratory National institute of Environmental Health Sciences Research Triangle Park NC 27709 United States; ^9^ Innovation Research Institute Xijing Hospital Fourth Military Medical University Xi'an 710032 China

**Keywords:** mechanical pain hypersensitivity, nociceptor, synaptic potentiation, TRPC3, TRPC6

## Abstract

Patients with tissue inflammation or injury often experience aberrant mechanical pain hypersensitivity, one of leading symptoms in clinic. Despite this, the molecular mechanisms underlying mechanical distortion are poorly understood. Canonical transient receptor potential (TRPC) channels confer sensitivity to mechanical stimulation. TRPC3 and TRPC6 proteins, coassembling as heterotetrameric channels, are highly expressed in sensory neurons. However, how these channels mediate mechanical pain hypersensitivity has remained elusive. It is shown that in mice and human, TRPC3 and TRPC6 are upregulated in DRG and spinal dorsal horn under pathological states. Double knockout of TRPC3/6 blunts mechanical pain hypersensitivity, largely by decreasing nociceptor hyperexcitability and spinal synaptic potentiation via presynaptic mechanism. In corroboration with this, nociceptor‐specific ablation of TRPC3/6 produces comparable pain relief. Mechanistic analysis reveals that upon peripheral inflammation, TRPC3/6 in primary sensory neurons get recruited via released bradykinin acting on B1/B2 receptors, facilitating BDNF secretion from spinal nociceptor terminals, which in turn potentiates synaptic transmission through TRPC3/6 and eventually results in mechanical pain hypersensitivity. Antagonizing TRPC3/6 in DRG relieves mechanical pain hypersensitivity in mice and nociceptor hyperexcitability in human. Thus, TRPC3/6 in nociceptors is crucially involved in pain plasticity and constitutes a promising therapeutic target against mechanical pain hypersensitivity with minor side effects.

## Graphical abstract

Sun et al. described how TRPC3/6 in primary sensory neurons get recruited via released bradykinin after peripheral inflammation, which in turn facilitates BDNF secretion from spinal nociceptor terminals and potentiates synaptic transmission and eventually contributes to mechanical pain hypersensitivity.

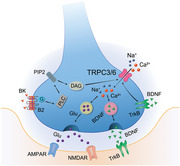

## 1 Introduction

Chronic pain represents a frequent and poorly understood medical problem. Mechanical pain hypersensitivity is one of the leading symptoms in patients with chronic, pathological pain and manifests as exaggerated responses to innocuous or noxious mechanical stimuli upon inflammation or injury.^[^ ^1^, ^2^ ^]^ This sensitized mechanical pain is assumed to be caused by peripheral sensitization or spinal amplification.^[^ ^1^, ^3^, ^4^ ^]^ However, the exact substrates underlying mechanical pain hypersensitivity at peripheral and spinal cord levels are not fully unraveled.

The detection of innocuous and noxious mechanical stimuli primarily relies on mechanically activated ion channels expressed in primary sensory neurons, i.e., low‐threshold mechanoreceptors (LTMRs) and nociceptors.^[^ ^5^, ^6^, ^7^ ^]^ In recent years, significant advances have been made in identifying several ion channels involved in mechanotransduction and mechanical sensitization, such as Piezo2,^[^ ^8^, ^9^, ^10^ ^]^ TACAN,^[^ ^11^, ^12^ ^]^ and TMC proteins.^[^ ^13^ ^]^ Apart from these, emerging evidence has accumulated that canonical transient receptor potential (TRPC) channels confer sensitivity to mechanical stimulation in vertebrate cells.^[^ ^14^ ^]^ TRPC channels form non‐selective cation channels that are permeable to Na^+^ and Ca^2+^ upon activation, contributing to membrane depolarization and initiation of Ca^2+^‐dependent intracellular cascades. Based on amino acid sequence homology and functional similarities, TRPC proteins appear to assemble as heterotetrametric channels within subgroups, one consisting of TRPC3/6/7 and the other of TRPC1/4/5.^[^ ^15^, ^16^, ^17^ ^]^ Within subgroups, functional redundancy and compensation are observed.^[^ ^14^, ^16^, ^18^, ^19^ ^]^ Previous studies using morphological approaches and high‐coverage single‐cell RNA‐sequencing demonstrate that TRPC3 and TRPC6 are highly expressed in primary sensory neurons of rodents and implicated in normal mechanotransduction in subsets of sensory neurons and cochlear hair cells.^[^ ^20^, ^21^, ^22^, ^23^, ^24^, ^25^, ^26^ ^]^ However, whether and how TRPC3 and TRPC6 mediate mechanical pain hypersensitivity under inflammatory states remains elusive.

Here, we sought to elucidate the exact role and underlying mechanisms of TRPC3 and TRPC6 in mechanical pain hypersensitivity in pain circuits at the DRG and spinal cord levels by combining genetic, behavioral, electrophysiological, biochemical, and fiber photometry recording approaches. Our results demonstrate that TRPC3 and TRPC6 display significant upregulation at DRG and the spinal dorsal horn in pathological pain states in mice or humans. Double knockout of TRPC3 and TRPC6 (TRPC3/6 DKO), but not single knockout of either subunit blunts mechanical pain and hypersensitivity in mice, in large part by decreasing nociceptor hyperexcitability and spinal synaptic potentiation via a presynaptic mechanism. In corroboration with this, specific genetic ablation of TRPC3/6 in nociceptors produces pain relief comparable to relief seen in mice with global TRPC3/6 deletion (DKO mice) designating TRPC3/6 in nociceptors as a critical molecular determinant for pronociceptive transmission. Further mechanistic analysis revealed that upon inflammation, TRPC3/6 in primary sensory neurons gets recruited via released bradykinin acting on its B1 and B2 receptors, facilitating the production and secretion of BDNF from spinal nociceptor terminals, which in turn potentiates synaptic transmission through TRPC3/6, and eventually resulting in mechanical pain hypersensitivity. Our findings unraveled the role of TRPC3/6 in nociceptors functioning in the detection of mechanical stimuli and mediation of mechanical pain hypersensitivity and revealed a feed‐forward regulatory network underlying this function. Moreover, pharmacologically antagonizing TRPC3/6 at DRG level relieves mechanical pain hypersensitivity in mice and nociceptor hyperexcitability in humans under pathological states. Hence, targeting TRPC3/6 in nociceptors action may represent what we believe to be a novel therapeutic strategy against mechanical pain hypersensitivity with least side effects.

## 2 Results

### 2.1 TRPC3/6 Deficiency Blunts Mechanical Pain Hypersensitivity

**Figure 1:**
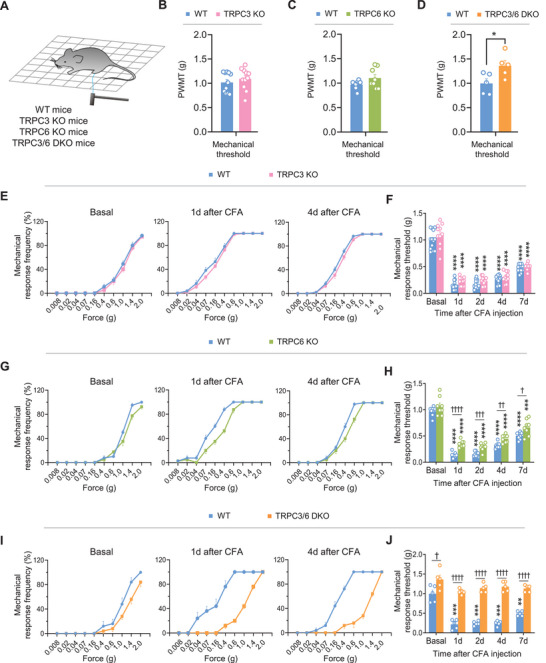
TRPC3/6 deficiency blunts mechanical pain hypersensitivity. A) Schematic illustration of mechanical behavioral testing in different genotypes of mice. B–D) Comparison of basal mechanical sensitivity in TRPC3 KO (B, n = 11), TRPC6 KO (C, n = 8), TRPC3/6 DKO (D, n = 5) mice and their corresponding wildtype (WT) littermates. *P < 0.05, by two‐tailed unpaired t‐test. E,G,I) Comparison of response frequency to von Frey hairs in TRPC3 KO (E), TRPC6 KO (G) and TRPC3/6 DKO (I) mice with WT littermates prior to and at 1d and 4d following CFA inflammation. F,H,J) Magnitude and time course of mechanical pain hypersensitivity to von Frey hairs application to ipsilateral hindpaws following CFA inflammation in TRPC3 KO (F, n = 11), TRPC6 KO (H, n = 8) and TRPC3/6 DKO (J, n = 5) mice as compared to their WT littermates. **P < 0.01, ***P < 0.001, ****P < 0.0001 at all‐time points for different genotypes versus basal by two‐way ANOVA, ^†^ *P* < 0.05, ^††^ *P* < 0.01, ^†††^ *P* < 0.001, ^††††^ *P* < 0.0001 at all‐time points for either TRPC3 KO, TRPC6 KO or TRPC3/6 DKO versus their WT littermates by two‐way ANOVA. Data are represented as mean ± S.E.M. See Table S2 (Supporting Information) for detailed statistical information. WT, wildtype; TRPC3 KO, TRPC3 knockout; TRPC6 KO, TRPC6 knockout; TRPC3/6 DKO, TRPC3/6 double knockout; PWMT, paw withdrawal mechanical threshold.

To investigate a specific role of TRPC3 and TRPC6 in pain regulation, we tested basal nociception and inflammatory pain hypersensitivity in TRPC3 knockout (TRPC3 KO), TRPC6 knockout (TRPC6 KO) and TRPC3/6 double knockout (TRPC3/6 DKO) mice. We first compared the basal mechanical sensitivity in these knockouts and their C57Bl6 wildtype (WT) controls. TRPC3 KO and TRPC6 KO mice did not show significant differences in von Frey test in comparison with their WT controls (**Figure** **1**A–C). TRPC3 and TRPC6 are known to heteromultimerize and show some functional redundancy in rat brain.^[^ ^14^, ^16^, ^19^ ^]^ We tested TRPC3/6 DKO mice for deficits in basal mechanical nociception. In comparison with WT controls, TRPC3/6 DKO mice displayed a blunted response to mechanical stimuli as compared to WT mice (Figure 1A,D). Motor‐coordination on a rotarod was normal in all knockout mouse lines as compared to WT controls (Figure S1A–C, Supporting Information). These data suggest that TRPC3 and TRPC6 are required for the normal mechanical sensitivity.

Next, we went on to address whether TRPC3 and TRPC6 are involved in the development of mechanical pain hypersensitivity associated with peripheral inflammation. Unilateral injection of complete Freund's adjuvant (CFA) into the intraplantar surface of hindpaw induced persistent inflammatory pain over 1 week, as characterized by leftward and upward shift in the stimulus‐response curve over basal curve, reflecting mechanical allodynia and hyperalgesia (Figure 1E). Similar to wildtype (WT) mice, TRPC3 KO mice demonstrated a comparable deviation over basal curve and identical relative drop in mechanical threshold upon CFA inflammation (Figure 1E,F). A mild, but significant reduction of mechanical allodynia and hyperalgesia was observed in CFA‐treated TRPC6 KO mice as compared to CFA‐treated WT mice (Figure 1G,H). To test whether TRPC3 and TRPC6 display redundancy in pain hypersensitivity, we further assessed TRPC3/6 DKO mice. In comparison with WT controls, TRPC3/6 DKO mice showed a prominent reduction in mechanical pain hypersensitivity upon CFA inflammation (Figure 1I,J). The magnitude of inhibition in mechanical pain hypersensitivity in TRPC3/6 DKO mice was stronger than that in TRPC6 KO and TRPC3 KO mice, confirming in vivo functional redundancy within TRPC3/6 complex. We thus focused our further mechanistic analyses using primarily TRPC3/6 DKO mice. These results indicate that TRPC3 and TRPC6 collectively contribute to mechanical pain hypersensitivity associated with tissue inflammation.

To determine whether deletion of TRPC3/6 induces aberrant developmental defects in sensory neurons, we quantified the number of DRG neurons and spinal dorsal horn neurons in WT and TRPC3/6 DKO mice. The percentages of CGRP‐expressing peptidergic neurons, and IB4‐labelled non‐peptidergic DRG neurons as well as NF200‐positive DRG neurons were not altered by deletion of TRPC3 and TRPC6 (Figure S2A–D, Supporting Information). Furthermore, central and peripheral patterning of peptidergic or non‐peptidergic nociceptors was normal in TRPC3/6 DKO mice, as revealed by immunostaining for CGRP and binding to IB4, respectively, in the spinal dorsal horn and skin (Figure S2E–G, Supporting Information). Neither did we see neuronal loss in the spinal cord, as revealed by NeuN staining (Figure S2H and I, Supporting Information). Taken together, we conclude from the above that the deficit in pain hypersensitivity in TRPC3/6 DKO mice is not due to developmental defects of peripheral sensory and spinal circuits.

### 2.2 TRPC3/6 is Responsible for the Coding of Cutaneous Mechanical Sensitivity in DRG and Spinal Cord Circuits

**Figure 2:**
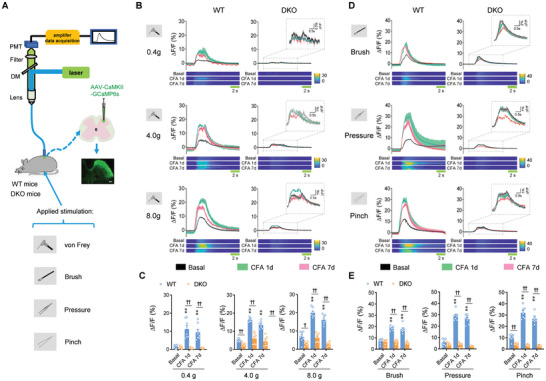
TRPC3/6 is responsible for the coding of cutaneous mechanical sensitivity in DRG and spinal cord circuits. A) Schematic diagram showing fiber photometry recording in spinal dorsal horn excitatory neurons in response to different types of mechanical stimulation applied to its receptive field. B) Typical examples of traces (upper panels) and heat maps (lower panels) of calcium transients induced by different forces of von Frey hairs prior to and at 1d and 7d following CFA inflammation in WT and TRPC3/6 DKO mice. Insets show the magnified calcium transients. C) Quantitative summary from 7 independent experiments of peak GCaMP6s signals evoked by von Frey hairs in WT and TRPC3/6 DKO mice prior to and at different time points after CFA injection. **P < 0.01 for WT CFA versus WT basal, ^#^ *P* < 0.05, ^##^ *P* < 0.01 for DKO versus WT, by Friedman's *M* test. D) Typical examples of traces (upper panels) and heat maps (lower panels) of calcium transients induced by brush, pressure and pinch of the receptive field prior to and at 1d and 7d following CFA inflammation in WT and TRPC3/6 DKO mice. Insets show the magnified calcium transients. E) Quantitative summary from 7 independent experiments of peak GCaMP6s signals evoked by brush, pressure and pinch stimuli in WT and TRPC3/6 DKO mice prior to and at different time points after CFA injection. **P < 0.01 for WT CFA versus WT basal, ^††^ *P* < 0.01 for DKO versus WT, by Friedman's *M* test. Data are represented as mean ± S.E.M. See Table S2 (Supporting Information) for detailed statistical information.

To elucidate whether TRPC3/6 encodes the mechanical sensitivity in pain circuits, we performed in vivo fiber photometry recording in the spinal dorsal horn. The activity of spinal excitatory projection neurons was monitored by stereotaxically injecting AAV2/9‐CaMKII‐GCaMP6s vectors into the lumbar spinal dorsal horn and measuring changes in GCaMP6s fluorescence in response to a wide range of mechanical stimuli applied to the cutaneous receptive field in the basal and CFA‐inflamed state from two genotypes (**Figure** **2**A). Aligning the GCaMP signals with video‐recorded stimulus application revealed that neuronal activity increased with increasing forces of von Frey filaments in naïve WT mice (Figure 2B). This neuronal activity induced by mechanical forces of von Frey filaments at 4–8 g but not 0.4 g was significantly attenuated in TRPC3/6 deficient mice in the basal state (Figure 2B,C). At different time points after CFA‐induced paw inflammation, neuronal activity of excitatory neurons exhibited a remarkable augmentation, manifesting as increased GCaMP6s fluorescence (Figure 2B,C). In striking contrast, this enhanced activity in the CFA‐inflamed state was largely attenuated by loss of TRPC3/6 (Figure 2B,C). These results indicate that TRPC3/6 mediates sensitivity to mechanical pain and inflammation‐induced mechanical pain hypersensitivity in the periphery and spinal circuits. This assumption was supported by analysis of three additional different types of mechanical stimuli, i.e., brush, pressure and pinch applied to the receptive field. Application of three types of mechanical stimuli elicited marked elevations of GCaMP6s fluorescence in spinal excitatory neurons from naïve WT mice, of which the pinch‐induced response but not the pressure‐ or the brush‐induced responses were attenuated in TRPC3/6 DKO mice (Figure 2D,E). Following CFA inflammation, a dramatic enhancement in GCaMP6 signals in spinal excitatory neurons were seen in response to brush, pressure and pinch in WT mice. This inflammation‐induced enhancement of neuronal activity failed to occur after deletion of TRPC3 and 6 (Figure 2D,E). Overall, these results infer that TRPC3/6 is responsible for the coding of cutaneous mechanical sensitivity in DRG and spinal cord circuits and mediates mechanical pain hypersensitivity under pathological states.

### 2.3 TRPC3 and TRPC6 are Upregulated in Mice DRG and Spinal Dorsal Horn as Well as Human DRG under Pathological States

**Figure 3:**
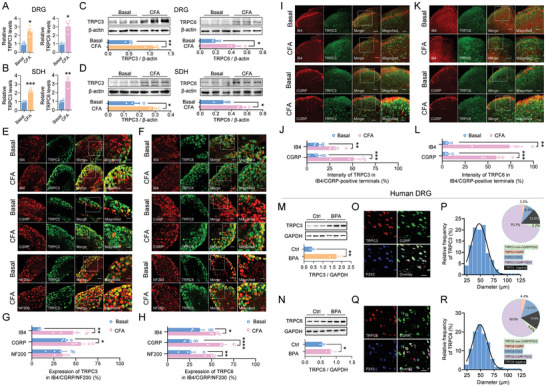
TRPC3 and TRPC6 are upregulated in DRG and spinal dorsal horn following peripheral injury in mice and human. A–D) TRPC3 and TRPC6 are upregulated in the DRG and spinal dorsal horn at both mRNA (A,B) (n = 4) and protein (C,D) (n = 3) levels at 24 h after CFA inflammation in WT mice. **P < 0.01, ***P < 0.001, by two‐tailed unpaired t‐test. E–H) Representative FISH images (E,F) and quantitative summary (G,H) showing the localization of TRPC3 and TRPC6 in different subtypes of DRG neurons and their upregulation upon CFA inflammation in mice (n = 4–5). *P < 0.05, **P < 0.01, ****P < 0.0001, by two‐tailed unpaired t‐test. Scale bar: 100 µm in the left three columns and 50 µm in right magnified panels in (E) and (F). I–L) Representative FISH images (I,K) and quantitative summary (J,L) showing the localization of TRPC3 and TRPC6 in the spinal dorsal horn and their upregulation upon CFA inflammation in WT mice (n = 4–5). **P < 0.01, ***P < 0.001, by two‐tailed unpaired t‐test. Scale bar: 100 µm in the left three columns and 50 µm in right magnified panels in (I) and (K). M,N) TRPC3 (M) (n = 3) and TRPC6 (N) (n = 3) are upregulated in DRGs of patients with brachial plexus avulsion as compared to controls (n = 3). *P < 0.05, **P < 0.01, by two‐tailed unpaired t‐test. O–R) Representative triple immunofluorescence images of DRG neurons from avulsed DRGs in BPA patients for TRPC3 with CGRP and P2X3 (O) and for TRPC6 with CGRP and P2X3 (Q). Scale bar: 100 µm in (O) and (Q). P,R) Relative frequency of TRPC3‐ (P) and TRPC6‐ (R) immunoreactive neurons across population of human DRG neurons after BPA injury. Bars are averages. Insets shown in (P) and (R) are quantitative summary of coexpression of TRPC3 or TRPC6 with CGRP and P2X3. n = 777 neurons for TRPC3 and 799 neurons for TRPC6 from three humans. Data are represented as mean ± S.E.M. See Table S2 (Supporting Information) for detailed statistical information. DRG, dorsal root ganglion; SDH, spinal dorsal horn; BPA, brachial plexus avulsion.

We next employed several different approaches to examine changes of TRPC3 and TRPC6 expression in DRG and spinal dorsal horn in inflammatory pain states in mice. The qRT‐PCR analysis revealed a significant upregulation of TRPC3 and TRPC6 at mRNA level in DRG and spinal dorsal horn at 24 h after CFA inflammation (**Figure** **3**A,B). Consistently, CFA inflammation induced a prominent increased expression of TRPC3 and TRPC6 at protein level in DRG as well as spinal dorsal horn (Figure 3C,D). To further characterize the TRPC3 and TRPC6 expression profiles and its changes in DRG and spinal dorsal horn under pathological states, we utilized the fluorescence in situ hybridization (FISH) assay. The specificity of TRPC3 and TRPC6 riboprobe was confirmed using TRPC3/6 DKO mice (Figure S3A and B, Supporting Information). FISH analysis showed that TRPC3 and TRPC6 are expressed in mouse DRG neurons, ranging from small‐ to large‐diameter neurons. Dual immunofluorescence experiments showed that TRPC3 and TRPC6 were highly expressed in isolectin B4 (IB4)‐labelled non‐peptidergic nociceptors, CGRP‐expressing peptidergic nociceptors as well as large‐diameter neurofilament‐200‐immunoreactive neurons in mouse DRG (Figure 3E,F). This expression profile is consistent with previous reports.^[^ ^21^, ^25^, ^27^ ^]^ Following peripheral inflammation, TRPC3 and TRPC6 expression exhibited significant upregulation in IB4‐labeled and CGRP‐expressing DRG neurons, and TRPC6 was also upregulated in NF200‐positive large DRG neurons (Figure 3E–H). In addition, we also observed expression of TRPC3 and TRPC6 in the spinal dorsal horn. As in DRG neurons, TRPC3 and TRPC6 are present in both IB4‐labeled non‐peptidergic and CGRP‐expressing peptidergic central terminals in the superficial dorsal horn (Figure 3I,K), which shows significant upregulation after CFA inflammation (Figure 3I–L). As expected, TRPC3 and TRPC6 was also found in the intrinsic spinal dorsal horn neurons and showed upregulation after CFA inflammation (Figure 3I,K). Ultrastructural preembedding double immunostaining further confirmed that TRPC3 and TRPC6 are localized in both presynaptic terminals of nociceptors as co‐labelled with CGRP and postsynaptic spinal neurons (Figure S4A–D, Supporting Information). The above expression of TRPC3 and TRPC6 in DRG was further confirmed by using RNAscope assay, showing the high colocalization of two subunits in DRG neurons (Figure S5A–D, Supporting Information).

In further support of a crucial role of TRPC3 and TRPC6 in pathological pain and its translational potential in clinic, we characterized TRPC3 and TRPC6 expression in human DRG samples under physiological and pathological pain states. We took avulsed DRG samples from brachial plexus avulsion (BPA) patients as described in detail in Methods. BPA patients and BPA mouse models exhibited chronic mechanical pain hypersensitivity.^[^ ^28^ ^]^ As compared to controls, BPA patients showed upregulation of TRPC3 and TRPC6 in the avulsed DRGs (Figure 3M,N). Immunofluorescence staining showed that TRPC3 and TRPC6 are observed in 86.5% and 84.1% of DRG neurons in BPA patients, respectively, particularly enriched in small to medium neurons ranging from 24–88 µm for TRPC3 and 24–96 µm for TRPC6 (Figure 3O–R). Within TRPC3^+^ or TRPC6^+^ population, 95.1% or 94.5% could be classified as nociceptors, expressing either CGRP or P2X3 (Figure 3O–R). We selected P2X3 expression rather than IB4‐binding here because IB4‐binding glycoproteins do not exist in primate DRGs.^[^ ^29^ ^]^ While antibodies against proteins for P2X3 and CGRP label distinct neuronal subpopulations in mice, these markers show substantial overlap in protein labeling in human.^[^ ^30^ ^]^ These results indicate a potential translation for TRPC3 and TRPC6 in clinical pain treatment.

### 2.4 Excitability of Nociceptive DRG Neurons is Substantially Reduced in TRPC3/6 DKO Mice

**Figure 4:**
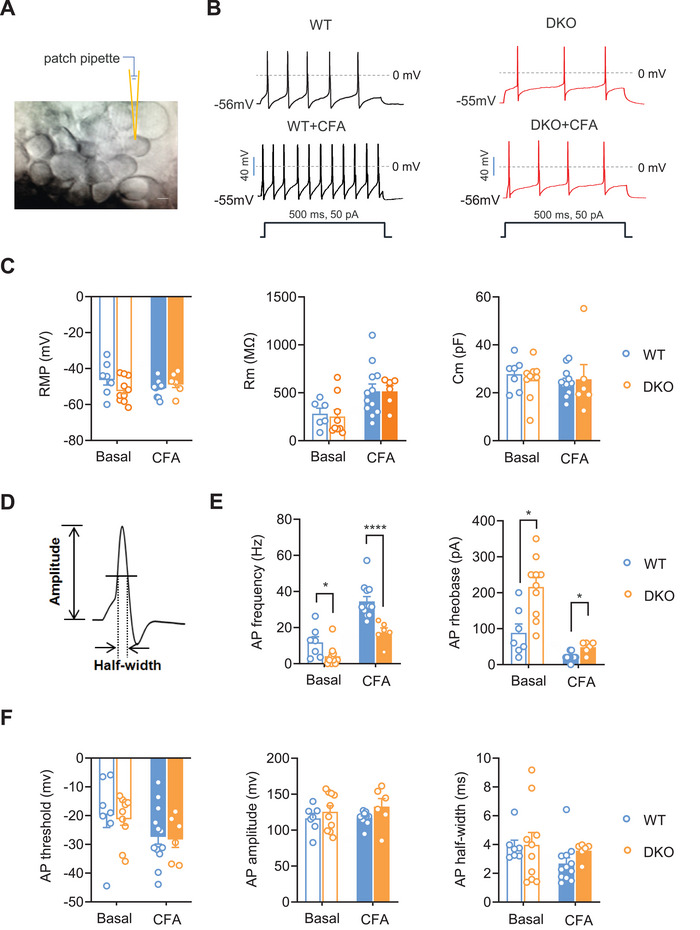
Excitability of nociceptive DRG neurons is substantially reduced in TRPC3/6 DKO mice. A) Photograph showing whole‐cell patch‐clamp recording from small‐diameter DRG neurons from WT and TRPC3/6 DKO mice. Scale bar: 10 µm. B) Action potentials (APs) induced by depolarizing current injection at 50 pA in DRG neurons from WT and TRPC3/6 DKO mice in both basal and CFA‐inflamed state (n = 6–12). C) Comparison of passive membrane properties including resting membrane potential, membrane resistance, membrane capacitance in small DRG neurons derived from WT and DKO mice in basal and inflamed state (n = 6–12). D) A representative AP evoked by depolarizing current injection and depiction of analysis of different parameters of AP. E,F) Comparison of active membrane properties of small DRG neurons from WT and DKO mice in either basal or CFA‐inflamed state, including AP frequency, AP rheobase, AP threshold, AP amplitude and AP half‐width (n = 6–12). *P < 0.05, ****P < 0.0001, by one‐way ANOVA and Kruskal‐Wallis *H* test. Data are represented as mean ± S.E.M. See Table S2 (Supporting Information) for detailed statistical information. RMP, resting membrane potential; Rm, membrane resistance; Cm, membrane capacitance; AP, action potential.

To determine the cellular and molecular mechanisms by which TRPC3 and TRPC6 regulate mechanical pain, we first employed patch‐clamp recordings in small‐diameter nociceptive DRG neurons from whole‐mount DRG preparations of WT and TRPC3/6 DKO mice (**Figure** **4**A,B). Passive membrane properties including resting membrane potential and membrane resistance as well as membrane capacitance were not significantly different between the two genotypes in either basal or CFA‐inflamed states (Figure 4C). However, the active membrane properties of small nociceptive DRG neurons derived from TRPC3/6 DKO mice represented marked differences compared with WT controls (Figure 4B,D–F). For example, the firing frequency evoked by a depolarizing current step was significantly lower in small DRG neurons from TRPC3/6 deficient mice than in WT mice in the basal state (Figure 4E). The rheobase required to evoke the action potential was much higher in TRPC3/6 DKO mice than WT mice (Figure 4E). Upon tissue inflammation, WT DRG neurons exhibited enhanced excitability, as characterized by increased firing frequency and lowered rheobase (Figure 4E). However, this hyperexcitability of small DRG neurons in CFA‐inflamed state was much lower in TRPC3/6 DKO mice (Figure 4E). The other parameters such as action potential threshold, amplitude as well as half‐width were not different between two genotypes (Figure 4F). The recorded small‐diameter DRG neurons were further confirmed to be nociceptive by using Neurobiotin added in the intrapipette and further co‐immunostaining with IB4 and anti‐CGRP (Figure S6A and B, Supporting Information). Overall, these data suggest that TRPC3/6 at DRG plays an important role in the regulation of nociceptor excitability and hyperexcitability after peripheral inflammation.

### 2.5 Efficacy of Spinal Synaptic Transmission and Plasticity is Compromised in TRPC3/6 DKO Mice

**Figure 5:**
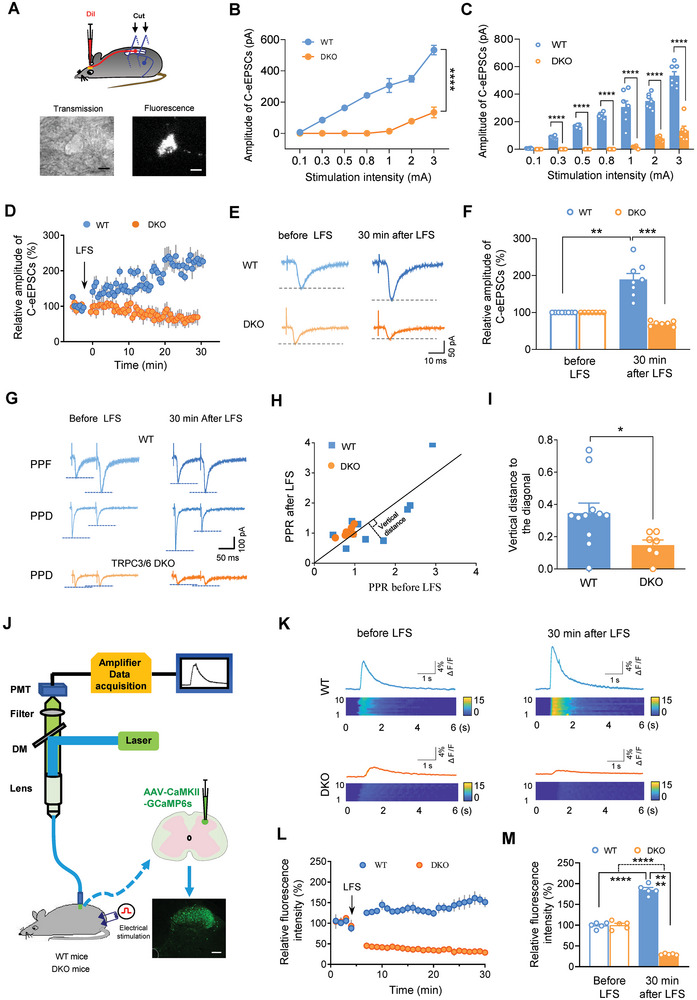
Efficacy of spinal synaptic transmission and plasticity is compromised in TRPC3/6 DKO mice. A) Schematic diagram showing whole‐cell patch‐clamp recording from spinal‐PAG projection neurons in spinal lamina I retrogradely labeled by DiI injection into contralateral ventrolateral PAG. B,C) Input‐output curves of C‐eEPSCs in spinal‐PAG projection neurons evoked by primary afferent stimulation at C‐fiber intensity from WT and TRPC3/6 DKO mice in the basal state. n = 7 for WT, n = 6 for DKO. *P < 0.05, ****P < 0.0001, by Friedman's *M* test for (B), Mann‐Whitney *U* test for (C). D–F) Time course (D), representative traces (E) and quantitative summary (F) of synaptic LTP induced by low‐frequency conditioning stimulation (LFS, 2 Hz, 3 mA) in WT and DKO mice. n = 10 for WT, n = 10 for DKO. **P < 0.01, ***P < 0.001, by Kruskal‐Wallis *H* test. G) Traces of typical recordings showing PPF or PPD of C‐ eEPSCs induced by pairs of pulses with an interval of 110 ms prior to and 30 min following LFS. H) Paired‐pulse ratio (PPR) prior to LFS is plotted against PPR at 30 min after LFS in WT and DKO mice. I) C‐eEPSCs recorded in WT mice showed clear change of PPR following LFS, which is significantly larger than DKO mice. n = 7–11. *P < 0.05, by two‐tailed unpaired t‐test. J) Schematic diagram showing fiber photometry recording in spinal dorsal horn excitatory neurons in response to electrical stimulation applied to its receptive field. K) Typical traces (upper panels) and heat map (lower panels) of calcium transients recorded in spinal excitatory neurons in response to a test electrical stimulation applied to the receptive field prior to and at 30 min after conditioning LFS in WT and DKO mice. L,M) Time course (L) and quantitative summary (M) of calcium transients prior to and after LFS in WT and DKO mice. n = 5 for WT, n = 5 for DKO. ****P < 0.0001, by one‐way ANOVA. Data are represented as mean ± S.E.M. See Table S2 (Supporting Information) for detailed statistical information. PPF, paired‐pulse facilitation; PPD, paired‐pulse depression.

Assuming a crucial role of TRPC3/6 in nociceptor excitability, we next aimed to test whether TRPC3/6 is involved in synaptic transmission and activity‐dependent synaptic plasticity at spinal synapses between nociceptive primary afferents and spinal lamina I projection neurons. Given that synaptic LTP evoked by natural, asynchronous low‐rate discharges in C‐nociceptors on spinal‐PAG (periaqueductal grey) projection neurons has been proposed to be a key cellular basis for pain hypersensitivity,^[^ ^4^, ^31^, ^32^, ^33^ ^]^ we recorded C‐fiber‐evoked EPSCs on lamina I spinal‐PAG projection neurons in dorsal root‐attached spinal cord slice preparation. Spinal‐PAG projection neurons were retrogradely labelled upon stereotactic injection of DiI in the PAG (**Figure** **5**A for schematic diagram). Basal synaptic transmission was analyzed by depicting detailed input‐output curves (I‐O curve) representing the relationship between the intensity of primary afferent stimulation and evoked EPSCs. As shown in Figure 5B, TRPC3/6 DKO mice showed a downward shift of I‐O curve as compared to WT mice, indicative of a reduced basal nociceptive transmission after loss of TRPC3/6 (Figure 5B,C). To further assess the role of TRPC3/6 on activity‐dependent synaptic plasticity, we then analyzed spinal LTP induced by a conditioning low frequency stimulation (LFS) of dorsal root at holding potential of ‐70 mV in WT and TRPC3/6 DKO mice. In spinal‐PAG projection neurons of WT mice, LFS (2 Hz, 2 min) of dorsal root produced LTP with a magnitude of more than 200% at 30 min (Figure 5D–F). In striking contrast, the same LFS did not evoke LTP and even led to LTD in spinal‐PAG projection neurons in TRPC3/6 DKO mice (Figure 5D–F). This result indicates that TRPC3/6 are key determinants in spinal nociceptive transmission and activity‐dependent synaptic plasticity at the first synapse of pain circuits.

We then assessed the relative contributions of TRPC3/6 via presynaptic and postsynaptic mechanisms to modulate spinal LTP in spinal‐PAG projection neurons. To address this, we performed the paired‐pulse ratio (PPR) analysis, which represents a short‐term plasticity and is well accepted as an indication of presynaptic mechanisms of long‐term potentiation in the hippocampus.^[^ ^34^ ^]^ In spinal slices from WT mice, we recorded paired‐pulse facilitation (PPF) in 47.6% of the recorded neurons as well as paired‐pulse depression (PPD) in 52.4% of the recorded neurons prior to conditioning LFS. In TRPC3/6 DKO mice, PPF was observed in 20% and PPD in 80% of the recorded neurons. Previous studies have shown that PPR (PPF or PPD) can decrease as well as increase in conjunction with LTP in a manner inversely proportional to the PPR prior to conditioning stimulus in brain regions, e.g. hippocampus,^[^ ^34^ ^]^ spinal cord.^[^ ^32^, ^33^, ^35^ ^]^ Upon application of LFS, a majority of neurons from WT mice demonstrated an obvious change in PPF or PPD (Figure 5G). We plotted the PPR of the entire cohort of recorded neurons at 30 min after LFS as a function of the basal PPR prior to LFS (Figure 5H). To evaluate the change of PPF and PPD altogether, we established a parameter which can reflect the magnitude of PPR change following LFS, that is the vertical distance of each spot shown in Figure 5H to the diagonal line (Figure 5H). This parameter has been verified previously.^[^ ^33^, ^36^ ^]^ The spots falling on the diagonal line represent no significant change of PPR. Quantification analysis revealed that WT mice showed much stronger changes of PPR after LFS relative to TRPC3/6 DKO mice (Figure 5I). Taken together, it can be inferred from the above that TRPC3/6 might modulate spinal synaptic plasticity via a presynaptic mechanism involving an increase of transmitter release probability from nociceptive primary afferents.

The crucial role of TRPC3/6 on spinal LTP was confirmed with alternative in vivo evidence by using fiber photometry imaging in the spinal cord. With injection of AAV2/9‐CaMKII‐GCaMP6s vectors into the spinal dorsal horn, we measured changes in the GCaMP6s fluorescence of spinal excitatory neurons in response to electrical stimuli in WT and TRPC3/6 DKO mice (Figure 5J for schematic diagram). As shown in Figure 5K, application of a test electrical stimulation to the peripheral receptive field at C‐fiber intensity (3 mA, 500 µs in duration) elicited a pronounced elevation in GCaMP6s fluorescence in spinal excitatory neurons (Figure 5K). Prior to conditioning LFS of the receptive field, test electrical stimulation‐induced GCaMP6s fluorescence signal was stable and constant (Figure 5L). Following conditioning LFS, the GCaMP6s signals displayed a long‐lasting potentiation by more than 150% at 30 min after LFS in WT mice (Figure 5K–M). In striking contrast, this potentiation of GCaMP6s signals was abolished and even converted to depression in TRPC3/6 DKO mice (Figure 5K–M). This data further indicates that a loss of TRPC3/6 was linked to a failure of activity‐dependent synaptic potentiation at circuits between nociceptive primary afferents and spinal dorsal horn.

### 2.6 TRPC3/6 Facilitates Activity‐Dependent Spinal Functional Plasticity in Inflammatory Pain States

**Figure 6:**
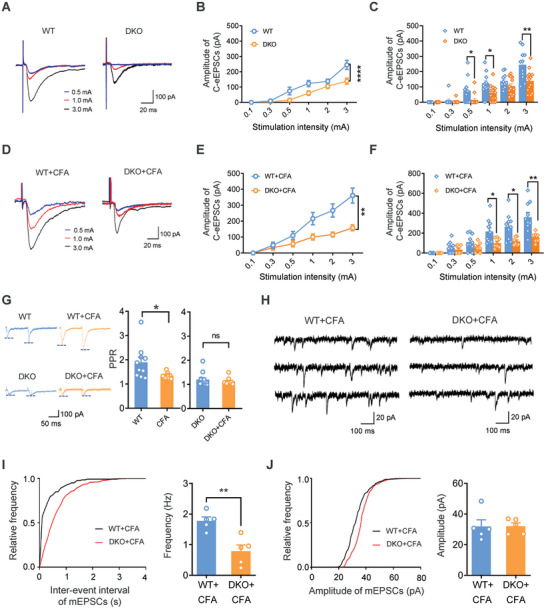
TRPC3/6 facilitates activity‐dependent spinal functional plasticity under inflammatory pain states. A–C) Typical traces (A) and quantitative summary (B,C) of C‐eEPSCs in spinal lamina I projection neurons evoked by primary afferent stimulation in WT and DKO mice in the basal state. n = 11 for WT, n = 13 for DKO. *P < 0.05 by Friedman's *M* test in (B), *P < 0.05, **P < 0.01 by Mann‐Whitney *U* test in (C). D–F) Typical traces (D) and quantitative summary (E,F) of C‐eEPSCs in spinal‐PAG projection neurons evoked by primary afferent stimulation in WT and DKO mice at 24 h after CFA inflammation. n = 11 for WT, n = 13 for DKO. *P < 0.05, **P < 0.01 by two‐way ANOVA. G) Typical examples (left panels) and quantitative summary of PPF (right panels) of C‐eEPSCs recorded in WT and DKO mice in both basal and CFA‐inflamed state. n = 10 for WT, n = 10 for DKO. *P < 0.05 by Mann‐Whitney *U* test. H) Representative traces of mEPSCs recorded in CFA‐inflamed WT and DKO mice. I,J) Cumulative curves and quantitative summary for mEPSCs frequency (I) and amplitude (J) derived from CFA‐treated WT and DKO mice. n = 5 for WT, n = 5 for DKO. **P < 0.01, by two‐tailed unpaired t‐test. Data are represented as mean ± S.E.M. See Table S2 (Supporting Information) for detailed statistical information.

Apart from electrically‐evoked synaptic plasticity, natural inflammation or injury are able to induce prolonged or long‐term changes in synaptic transmission in the spinal cord.^[^ ^3^, ^4^, ^37^ ^]^ Next, we further assessed the involvement of TRPC3/6 in the natural inflammation‐induced synaptic potentiation at the spinal level. C‐fiber‐evoked EPSCs were recorded in spinal lamina I projection neurons from WT and TRPC3/6 DKO mice in the basal as well as CFA‐inflamed states. Consistent with the above, TRPC3/6 DKO mice showed a reduced basal synaptic transmission as compared to WT mice, characterized by a downward shift of I‐O curve of eEPSCs (**Figure** **6**A–C). In WT controls, peripheral inflammation produced a prominent synaptic potentiation, as characterized by a significant upward shift in the I‐O curve over basal curve (Figure 6D–F). The magnitude of C‐eEPSCs was greatly enhanced after CFA inflammation (Figure 6F). In striking contrast, TRPC3/6 DKO mice displayed very little deviation in I‐O curve over basal curve in CFA‐inflamed state (Figure 6D–F). Comparison of the magnitude between two genotypes revealed that CFA‐induced enhancement of C‐eEPSCs was impaired in TRPC3/6 deficient mice (Figure 6F). These results suggest that TRPC3/6 contributes to spinal synaptic potentiation after peripheral inflammation. To elucidate whether presynaptic or postsynaptic mechanism is involved in the modulation of synaptic potentiation by TRPC3/6, PPR and mEPSCs analysis were performed in WT and TRPC3/6 DKO mice in the CFA‐inflamed state. As shown in Figure 6G, following CFA inflammation, WT mice showed a reduced PPR of C‐eEPSCs, indicating an increased transmitter release probability. In contrast, this reduction in PPR after paw inflammation was not observed after deletion of TRPC3/6 (Figure 6G). Furthermore, analysis of mEPSCs revealed that loss of TRPC3/6 induced a marked reduction in the frequency but no alteration in the amplitude of mEPSCs in the inflammatory state (Figure 6H–J). These two lines of evidence collectively indicate that TRPC3/6 potentiates spinal synaptic transmission upon inflammation at least in part via a presynaptic mechanism involving increased transmitter release probability from central terminals of nociceptive primary afferent inputs.

### 2.7 TRPC3/6 Facilitates Production and Secretion of BDNF from Primary Sensory Neurons upon Inflammation

**Figure 7:**
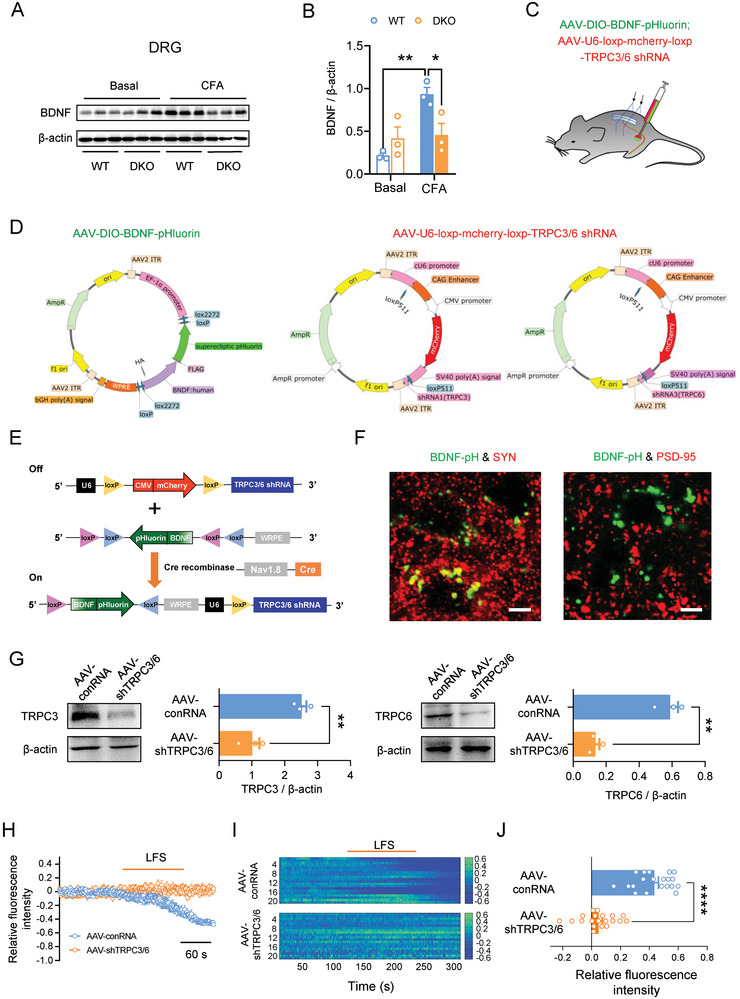
TRPC3/6 facilitates production and secretion of BDNF from primary sensory neurons upon inflammation. A,B) Typical example (A) and quantitative summary (B) showing levels of BDNF in L3‐L4 DRGs derived from WT and DKO mice prior to and at 24 h after CFA inflammation (n = 3 mice in each lane). *P < 0.05, **P < 0.01, by one‐way ANOVA. C) Scheme illustrating the experimental approach for imaging activity‐induced changes in BDNF‐pHluorin fluorescence from spinal terminals of nociceptors in SNS‐Cre and nociceptor‐specific TRPC3/6 knockdown mice. Nociceptor‐specific knockdown of TRPC3/6 is achieved via injection of Cre‐dependent AAV2/8 shTRPC3/6 into L3‐L4 DRGs in SNS‐Cre mice. D,E) Schematic diagram showing the construction of AAV2/8‐EF1a‐DIO‐BDNF‐pHluorin and Cre‐dependent AAV2/8 expressing shRNA TRPC3/6 (AAV2/8‐U6‐loxp‐CMV‐mcherry‐loxp‐shRNA TRPC3/6). F) Double immunofluorescence staining images showing colocalization of BDNF‐pHluorin (BDNF‐pH) puncta with the presynaptic marker synaptophysin (SYN), but juxtaposition with the postsynaptic marker PSD‐95. Scale bar: 5 µm. G) Western blot analysis with anti‐TRPC3 and anti‐TRPC6 antibody confirmed the efficient knockdown of TRPC3/6 by the above approach. **P < 0.01, by two‐tailed unpaired t‐ test. H–J) Sample traces (H), heat map (I) and quantitative summary (J) of BDNF‐pHluorin fluorescence changes evoked by LFS after intraganglionic injection of AAV2/8 conRNA and AAV2/8 shTRPC3/6 in SNS‐Cre mice. ****P < 0.0001, by two‐tailed unpaired t‐test. Data are represented as mean ± S.E.M. See Table S2 (Supporting Information) for detailed statistical information.

BDNF is known to be required for the induction of synaptic potentiation at some glutamatergic synapses including spinal synapses and further pain hypersensitivity.^[^ ^38^, ^39^, ^40^, ^41^, ^42^, ^43^, ^44^ ^]^ We therefore attempted to test whether TRPC3/6 regulates the BDNF response in pain circuits at DRG and spinal cord levels. In WT mice, persistent activation of nociceptors by CFA inflammation produced prominent upregulation of BDNF production in lysates from L3/4 DRGs over the basal state (**Figure** **7**A,B). This effect was largely reduced in mice devoid of TRPC3/6, indicating the dependence of BDNF production upon activation of TRPC3/6 in DRGs (Figure 7A,B).

Subsequently, we specifically examined TRPC3/6‐dependent BDNF secretion from spinal nociceptor terminals by expressing BDNF tagged with a pH‐sensitive fluorescent protein (superecliptic pHluorin; BDNF‐pH), which is known to undergo the same intracellular processing and exhibits similar biological activity as the native BDNF.^[^ ^45^ ^]^ BDNF‐pH was restrictedly expressed in presynaptic nociceptor terminals by injection of an AAV2/8 vector containing double‐floxed inversed BDNF‐pH codon into L3/L4 DRGs of SNS‐Cre mice under the control of the Nav1.8 promoter (Figure 7C–E). Meanwhile, by using Cre‐loxP approaches, specific deletion of TRPC3/6 in nociceptors was achieved via coinjection of loxP‐TRPC3 shRNA‐expressing AAV2/8 and loxP‐TRPC6 shRNA‐expressing AAV2/8 into L3/L4 DRGs of SNS‐Cre mice (Figure 7C–E). SNS‐Cre mice were generated to express Cre recombinase selectively in nociceptive DRG or TG (trigeminal ganglion) neurons under Nav1.8 promoter and thus enable selective modulation of genes in nociceptive sensory neurons.^[^ ^46^ ^]^ At 4 weeks following stable virus expression, high‐level BDNF‐pH expression was observed in spinal nociceptor terminals, as verified by axonal BDNF‐pH puncta largely localized to areas marked with the presynaptic marker synaptophysin and juxtaposed to the postsynaptic marker PSD‐95 (Figure 7F). Western blotting analysis confirmed the efficiency of TRPC3/6 knockdown by this virion strategy (Figure 7G). Using super‐resolution confocal microscopy of dorsal root‐attached spinal slices, activity‐induced BDNF secretion was monitored by changes in the fluorescence intensity of BDNF‐pH puncta. Following LFS applied to dorsal root, we demonstrated that most fluorescent puncta displayed fusion with secretion, with a dramatic reduction of BDNF‐pH fluorescence below the basal level in spinal slices derived from SNS‐Cre mice (Figure 7H–J). In striking contrast, knockdown of TRPC3/6 in nociceptors abolished BDNF secretion induced by LFS of dorsal root (Figure 7H–J). These results suggest that presynaptic TRPC3/6 facilitates activity‐dependent BDNF secretion from spinal nociceptor terminals.

### 2.8 Marked Defects in Inflammatory Pain Hypersensitivity in Nociceptor‐Specific Loss of TRPC3/6 as well as BDNF

**Figure 8:**
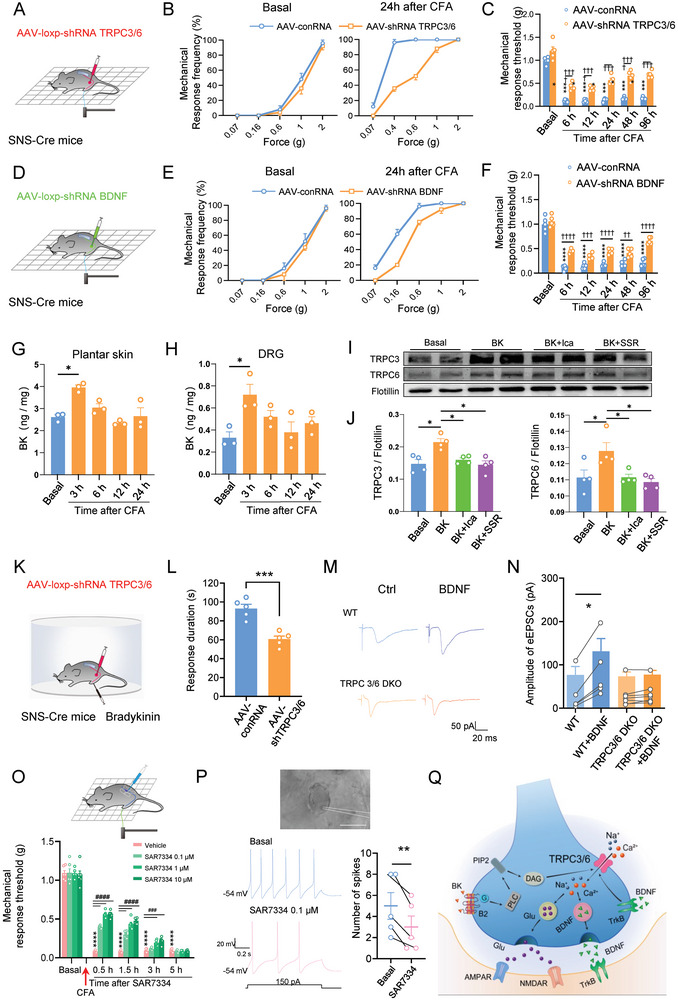
Marked defects in inflammatory pain hypersensitivity in nociceptor‐specific loss of TRPC3/6 as well as BDNF. A) Schematic illustration of expression of AAV2/8‐loxp‐shRNA TRPC3/6 in L3/L4 DRGs of SNS‐Cre mice for nociceptor‐ specific knockdown of TRPC3/6. B,C) Stimulus‐response curve (B) and mechanical response threshold (C) at different time points to von Frey hairs applied to the ipsilateral hindpaw following peripheral CFA inflammation in SNS‐Cre mice expressing shRNA TRPC3/6 and shRNA control. n = 5, ****P < 0.0001 at all‐time points for AAV‐conRNA versus basal, ^†††^ *P* < 0.001, ^††††^ *P* < 0.0001 at all‐time points for AAV‐shRNA TRPC3/6 versus AAV‐conRNA, by two‐way ANOVA. D) Schematic illustration of specific knockdown of BDNF in nociceptors via injection of AAV2/8‐loxp shRNA BDNF into L3/L4 DRGs of SNS‐Cre mice. E,F) Stimulus‐response curve (E) and mechanical threshold (F) at different time points to von Frey hairs applied to the ipsilateral hindpaw following CFA inflammation in SNS‐Cre mice expressing shRNA BDNF and shRNA control. n = 5, ****P < 0.0001 at all‐time points for AAV‐conRNA versus basal, ^††^ *P* < 0.01, ^††††^ *P* < 0.0001 at all‐time points for AAV‐shRNA BDNF versus AAV‐conRNA, by two‐way ANOVA. G,H) ELISA assay showing the increased production of bradykinin in the inflamed hindpaw (G) and lumbar DRGs (H) at different time points following CFA inflammation. n = 3, *P < 0.05, by two‐way unpaired t‐test. I,J) Representative immunoblots (I) and quantitative summary (J) showing the expression of TRPC3 and TRPC6 in the membrane fraction of DRG tissue after incubation with bradykinin for 1 h without and with the presence of B1 receptor antagonist, SSR240612 (10 µM) or B2 receptor antagonist, Icatibant (50 nM). Ica, Icatibant; SSR, SSR240612. K,L) Schematic illustration (K) and quantitative summary (L) showing that nociceptor‐specific knockdown of TRPC3/6 attenuates bradykinin‐induced spontaneous nociception. n = 5, ***P < 0.001, by two‐way unpaired t‐test. M,N) Representative traces (M) and quantitative summary (N) showing that BDNF‐induced synaptic potentiation is eliminated in TRPC3/6 DKO mice as compared to WT mice. n = 5–7, *P < 0.05, by Friedman's *M* test. O) Intervertebral foramen injection of TRPC3/6 antagonist, SAR7334 produced a dose‐dependent relief of CFA‐induced mechanical pain hypersensitivity in WT mice. n = 6, ****P < 0.0001 for Vehicle + CFA versus basal, ^###^ *P* < 0.001, ^####^ *P* < 0.0001 for SAR7334 + CFA versus Vehicle + CFA, by two‐way ANOVA. P) Upper panel showing whole‐cell patch clamp recordings from typical human small DRG neurons. Lower panels showing typical examples and quantitative summary showing that bath application of TRPC3/6 antagonist, SAR7334 (0.1 µM) largely attenuated the firing frequency induced by depolarizing current injection in small DRG neurons from BPA patients. n = 5, **P < 0.01, by paired t‐test. Scale bar: 50 µm. Q) A schematic model proposing how TRPC3/6 channels get recruited upon inflammation and mediates mechanical pain hypersensitivity via feed‐forward regulatory network (see text for details). Data are represented as mean ± S.E.M. See Table S2 (Supporting Information) for detailed statistical information.

We went on to reveal whether TRPC3/6‐BDNF signaling pathway from nociceptors play a crucial role in the development of mechanical pain hypersensitivity upon inflammation. Following CFA‐induced hindpaw inflammation, SNS‐Cre mice injected with AAV2/8‐conRNA in L3/4 DRGs exhibited a notable mechanical allodynia and hyperalgesia at different time points (**Figure** **8**A–C). By contrast, this mechanical pain hypersensitivity was apparently attenuated in mice expressing AAV‐shRNA TRPC3/6 in nociceptors (Figure 8A–C). Likewise, nociceptor‐specific knockdown of BDNF was able to reduce CFA‐induced mechanical pain hypersensitivity in comparison with control group (Figure 8D–F). Immunofluorescence and western blotting analysis confirmed a nociceptor‐specific loss of BDNF by this virion strategy (Figure S7A–D, Supporting Information).

We further identified how the TRPC3/6‐BDNF signaling pathways in nociceptors are recruited under inflammatory pain states. Pioneering functional and structural evidence demonstrated that TRPC3 and TRPC6 channels get activated by PLC stimulation and diacylglycerol (DAG) production in response to G protein coupled receptors (GPCRs) in different biological system.^[^ ^47^, ^48^, ^49^, ^50^, ^51^ ^]^ Bradykinin is one of the most important inflammatory mediators upon tissue inflammation and plays a crucial role in mediating pain sensitization via interaction with B2/B1 G‐protein‐coupled receptors (GPCRs).^[^ ^52^, ^53^ ^]^ In our case, we observed using ELISA assay that intraplantar CFA inflammation elicits increased production of bradykinin in the inflamed area and DRG at 3 h post‐CFA, returning gradually to the basal level at 12 h post‐CFA, which is consistent with its short half‐life ^[^ ^54^, ^55^ ^]^ (Figure 8G,H). Incubation of lumbar DRGs from WT mice with bradykinin (100 nM) for 1 h was found to increase TRPC3 and TRPC6 expression in the membrane fraction of DRG as compared to vehicle treatment (Figure 8I,J). Further analysis revealed that this increased membrane trafficking of TRPC3 and TRPC6 induced by bradykinin is significantly attenuated by Icatibant (50 nM), a B2 receptor antagonist, or SSR240612 (10 µM), a B1 receptor antagonist, indicating the involvement of B2 and B1 receptors in the interaction between bradykinin and TRPC3 and TRPC6 (Figure 8I,J). At behavioral level, intraplantar injection of bradykinin elicited a robust spontaneous nocifensive response, as characterized by lifting and licking of the injected paw in SNS‐Cre mice expressing AAV2/8‐conRNA (Figure 8K,L). In striking contrast, this bradykinin‐induced nocifensive response was much lowered in SNS‐Cre mice expressing AAV2/8‐shRNA TRPC3/6 (Figure 8K,L). This led us to infer that TRPC3/6, highly expressed in rodent nociceptors, is coupled to bradykinin receptor, a pro‐inflammatory metabotropic receptor and trigger downstream BDNF production and secretion, which in turn leads to mechanical pain hypersensitivity upon inflammation. In the other way around, previous studies have shown that BDNF regulates several neuronal activities through activation of TRPC channels.^[^ ^56^, ^57^ ^]^ Whether released BDNF potentiates spinal synaptic transmission upon inflammation in a TRPC3/6‐dependent manner remained unknown. We tested this possibility by observing the regulation of C fiber‐evoked EPSCs (C‐eEPSCs) by BDNF application in WT and TRPC3/6 DKO mice. As shown in Figure 8M,N, bath application of BDNF (100 ng ml^−1^) augmented the amplitude of C‐fiber‐evoked EPSCs (C‐eEPSCs) in spinal lamina I neurons from WT mice, which did not come about in TRPC3/6 DKO mice (Figure 8M,N). Taken together, our results uncover a feed‐forward regulatory network driven by TRPC3/6 in nociceptors that may facilitate nociceptor excitability and synaptic transmission and hence promote mechanical pain hypersensitivity.

Last, we sought to determine whether TRPC3/6 possesses any potential clinical implication as a crucial target in nociceptors for analgesia. To this end, we performed intervertebral foramen injection of SAR7334, a TRPC3/6 antagonist, at 24 h after CFA inflammation. As compared to vehicle group, intervertebral foramen delivery of SAR7334 dose‐dependently attenuated CFA‐induced mechanical pain hypersensitivity (Figure 8O). More importantly, in whole‐mount DRG preparation from BPA patients (see details in Methods), bath application of SAR7334 (0.1 µM) largely inhibited the firing frequency induced by depolarizing current injection in human small DRG neurons (Figure 8P). This inhibitory effect in human DRG neuronal hyperexcitability was also seen for another TRPC3/6 antagonist, GSK283 (10 µM) (Figure S8A–C, Supporting Information). These strongly suggest the potential clinical implication of TRPC3/6 as a key target in nociceptors in treating mechanical pain sensitization. Since the DRG lies outside of the blood‐brain barrier,^[^ ^58^ ^]^ it can be inferred that targeting TRPC3/6 in nociceptors represents a promising strategy for fulfillment of optimal analgesic therapeutics with least side effects.

## 3 Discussion

This study demonstrates that TRPC3 and TRPC6 are together required for mechanical pain and hypersensitivity induced by inflammation, as schematically illustrated in the working model in Figure 8Q. Following inflammation, proinflammatory mediators including bradykinin gets released, which activates its corresponding G‐protein‐coupled receptors B2 and B1, leading to membrane trafficking of TRPC3/6 channels in nociceptive DRG neurons and elevation of intracellular Ca^2+^ levels and hence DRG neuronal hyperexcitability. This results in increased production and secretion of BDNF from spinal nociceptor terminals, which further enhances synaptic transmission through TRPC3/6 channels. Thus, this study primarily clarifies how TRPC3/6 channels get recruited upon inflammation and mediates mechanical pain hypersensitivity via a feed‐forward regulatory network.

### 3.1 TRPC3/6 is Required for Mechanical Pain and Hypersensitivity Following Peripheral Inflammation

The novel and most important findings of this study are that we identify TRPC3/6 as the critical mediator for mechanical pain and hypersensitivity upon peripheral inflammation. Members of the broadly expressed TRPC family have been shown to be activated by mechanical stimuli in a variety of biological systems, i.e., cardiovascular system, skeletal muscle, airway smooth muscle, endothelial cells, nervous system.^[^ ^14^ ^]^ Despite this, whether TRPC protein act as a direct mechanosensor remains problematic, because overexpression of these subunits in HEK293 cells does not induce mechanosensitive currents.^[^ ^59^ ^]^ However, overexpression of TRPC3 and TRPC6 in ND‐C cells, a sensory neuron‐derived cell line,^[^ ^60^ ^]^ has been shown to elicit mechanically gated inward currents.^[^ ^25^ ^]^ Furthermore, deletion of TRPC3 and TRPC6 silences half of small‐diameter sensory neurons expressing mechanically activated rapidly adapting (RA) currents.^[^ ^25^ ^]^ These lines of evidence provide a hint that TRPC3 and TRPC6 are at least potential components of mechanotransducing complexes. In support of this assumption, our present study demonstrated that TRPC3/6 are responsible for the coding of cutaneous mechanical sensitivity in DRG and spinal cord circuits. This is characterized by the elevated GCaMP6s signals using fiber photometry recording in spinal excitatory neurons in response to a variety of cutaneous mechanical stimuli in WT mice and prominent reduction in GCaMP6s responses in TRPC3/6 DKO mice. In agreement with this neuronal response to mechanical stimuli, double knockout of TRPC3/6 blunts the basal mechanical pain, which is consistent with a previous observation.^[^ ^25^ ^]^ Thus, following peripheral inflammation, TRPC3/6 are shown to be significantly upregulated in DRG and spinal cord of mice as well as in DRG of human, and this further enhances mechanical stimuli‐induced GCaMP6s signals in spinal neurons and hence induces mechanical allodynia and hyperalgesia in behavior. Genetic ablation of TRPC3/6 reverses these effects at both neuronal and behavioral levels. In contrast, single knockout of TRPC3 or TRPC6 does not alter or slightly reduces mechanical pain response and allodynia. This indicates that TRPC3 and TRPC6 may show some compensation in sensory functions, consistent with their functional redundancy observed in other systems.^[^ ^14^, ^16^, ^18^, ^19^ ^]^ Overall, these results provide strong evidence that TRPC3/6 are key determinants for detection of mechanical stimuli and mediation of mechanical pain and hypersensitivity. Consistent with our observation, TRPC6 is shown to act in concert with TRPV4 to mediate mechanical hyperalgesia induced by carrageenan and paclitaxel chemotherapy.^[^ ^61^ ^]^ In a human study, TRPC6 has been linked with the degeneration of intervertebral disc and development of low back pain.^[^ ^62^ ^]^

### 3.2 TRPC3/6 Facilitates Mechanical Pain and Hypersensitivity via Enhancement of Nociceptor Excitability and Spinal Synaptic Transmission

Another intriguing finding of the present study is that we uncovered the cellular basis for TRPC3/6 to mediate mechanical pain and sensitization. Mounting evidence has shown that peripheral pain sensing neurons, called nociceptors, are critical origins of pain caused by inflammation or injury.^[^ ^63^, ^64^, ^65^, ^66^, ^67^, ^68^ ^]^ Aberrant hyperexcitability in nociceptors is assumed to be a key driver for the generation of chronic pain states.^[^ ^6^, ^64^, ^69^, ^70^, ^71^ ^]^ The TRPC proteins are known to form non‐selective cationic channels, whose activation enables entry of Ca^2+^ and Na^+^ into the cell, leading to membrane depolarization.^[^ ^72^ ^]^ It is thus reasonable to speculate that TRPC3/6 may facilitate mechanical pain and hypersensitivity via enhancement of nociceptor excitability. Indeed, this assumption was confirmed by our observation that TRPC3/6 deficiency reduces the excitability of nociceptors in the basal state and alleviated the magnitude of hyperexcitability caused by peripheral inflammation. This data suggests a crucial role of TRPC3/6 in the determination of nociceptor excitability. Consistent with our results, TRPC3 is shown to be required for IgG immune complex‐induced excitation of rat DRG neurons.^[^ ^73^ ^]^

Synapse between peripheral nociceptors and spinal neurons is the first gate conveying peripheral nociceptive information to the central nervous system and its plasticity is considered as a cellular basis for the development and maintenance of pain hypersensitivity following inflammation or injury.^[^ ^3^, ^4^, ^7^, ^32^, ^69^, ^74^ ^]^ LTP can be triggered at spinal synapses between nociceptor terminals and spinal‐PAG projection neurons by activation of nociceptive nerve afferents at low‐rate frequencies relevant to pathological pain.^[^ ^4^, ^31^, ^32^, ^33^ ^]^ This synaptic potentiation is assumed to require presynaptic mechanisms involving an increase in transmitter release probability from presynaptic nociceptor terminals.^[^ ^4^, ^32^, ^33^, ^75^ ^]^ Considering the crucial role of TRPC3/6 in determining nociceptor excitability, we would expect that spinal synaptic transmission and potentiation may be regulated subsequently by TRPC3/6. In the present study, recordings of C‐eEPSCs in spinal‐PAG projection neurons showed that spinal synaptic transmission and activity‐dependent synaptic plasticity are compromised by loss of TRPC3/6. Of note, spinal LTP evoked by conditioning stimulation is even converted to LTD in TRPC3/6 DKO mice. PPR and mEPSCs analysis reveal a presynaptic TRPC3/6 function involving an increase of transmitter release probability. This presynaptic regulation is strongly supported by our FISH and ultrastructural data showing dense expression of TRPC3/6 in CGRP‐expressing peptidergic and IB4‐binding nonpeptidergic terminals in the spinal dorsal horn. Cumulatively, we can infer from the above that TRPC3/6 facilitates mechanical pain and hypersensitivity via enhancement of nociceptor excitability and subsequent spinal synaptic potentiation through a presynaptic mechanism involving increased transmitter release probability from central terminals of nociceptors.

### 3.3 TRPC3/6‐BDNF Signaling Pathway in Nociceptors Facilitates Mechanical Pain Hypersensitivity

Exactly how TRPC3/6 accomplishes the above action at DRG, and spinal cord circuits remains elusive so far. Previous studies have demonstrated the crucial role of BDNF in spinal synaptic potentiation and pain hypersensitivity.^[^ ^38^, ^39^, ^40^, ^41^, ^42^, ^43^, ^44^ ^]^ However, the exact sources of BDNF, i.e., presynaptically‐ or postsynaptically‐ or even glial cell‐derived, and precise mechanisms of action have remained unclear. Given the reported Ca^2+^‐dependent BDNF secretion^[^ ^45^, ^76^, ^77^ ^]^ together with Ca^2+^ influx upon TRPC3/6 activation^[^ ^72^ ^]^ (and our present results), we proposed a hypothesis that TRPC3/6 expressed in primary sensory neurons may act upstream to cause BDNF production and further its secretion from spinal terminals of primary afferents, which in turn results in nociceptor hyperexcitability and spinal synaptic potentiation and eventually pain hypersensitivity. In agreement with this assumption, we did observe that repetitive activation of nociceptors by peripheral inflammation leads to BDNF production in a TRPC3/6‐dependent manner in DRG. Further imaging of spinal nociceptor terminals expressing BDNF‐pH in spinal slices reveals a TRPC3/6‐dependent BDNF secretion from presynaptic nociceptor terminals. Thus, our current results provide the direct evidence that activation of TRPC3/6 in primary sensory neurons is responsible for triggering activity‐dependent BDNF production and secretion from primary nociceptive neurons. Similar to our observation, TRPC3 has also been reported to regulate BDNF release in human airway smooth muscle.^[^ ^78^ ^]^ The other way around, we further showed that this BDNF secretion in turn can potentiate spinal synaptic transmission through activation of TRPC3/6, as characterized by the impaired augmentation of C‐eEPSCs by BDNF application in TRPC3/6‐deficient mice. This is consistent with previous reports showing TRPC3/6 acts as a downstream target for BDNF in other systems, i.e., hippocampal neurons or olfactory ensheathing cells.^[^ ^56^, ^57^ ^]^ Together, our results uncovered a feed‐forward regulatory network between TRPC3/6 activation and BDNF secretion in nociceptors facilitating nociceptor excitability and spinal synaptic transmission. Apart from BDNF from spinal nociceptor terminals, some studies have reported the origin of microglia for BDNF in mediating pain hypersensitivity caused by nerve injury as well as spinal synaptic plasticity induced by conditioning stimulus.^[^ ^79^, ^80^, ^81^, ^82^ ^]^ However, recent evidence on microglia RNAseq in the spinal dorsal horn suggest a lack of expression of the *bdnf* gene in microglia, while primary afferents do express BDNF.^[^ ^83^ ^]^ It remains to be further investigated whether BDNF from microglia takes part in facilitating TRPC3/6 function in spinal nociceptor terminals and its subsequent signaling cascades.

### 3.4 Targeting Peripheral TRPC3/6 Represents a Novel Therapeutic Intervention for Analgesia with Least Side Effects

In an effort to specifically link cellular changes described above at DRG and spinal cord circuits to changes in nociceptive behavior, we conditionally ablated TRPC3/6 in nociceptors and observed a comparable antinociceptive effects after CFA inflammation with global TRPC3/6 deletion. Thus, our study implicates TRPC3/6 in nociceptors as key mediators for the development of mechanical pain hypersensitivity associated with peripheral inflammation. Similarly, nociceptor‐specific knockdown of BDNF produces significant analgesia, supporting the involvement of TRPC3/6‐BDNF signaling cascades. However, one question still remains regarding how TRPC3/6 gets recruited in inflammatory pain states. TRPC3/6 have been shown to be activated by PLC stimulation and DAG production in response to GPCRs in various biological systems.^[^ ^47^, ^48^, ^49^, ^50^, ^51^ ^]^ Bradykinin is one of well‐established inflammatory mediator upon tissue inflammation and has been implicated in pain sensitization via interaction with B1/B2 GPCRs.^[^ ^52^, ^53^ ^]^ To establish whether TRPC3/6 gets recruited upon bradykinin release caused by inflammation, we observed that CFA inflammation leads to two‐fold increase of bradykinin in the inflamed area and lumbar DRGs using ELISA assay. Incubation of lumbar DRGs from naïve mice with bradykinin caused significant increase of TRPC3 and TRPC6 expression in the membrane fraction of DRG tissue, which is mediated by B2/B1 receptors. This cellular interaction bears significance in pain sensitization. Specific knockdown of TRPC3/6 in nociceptors largely reduced spontaneous nocifensive response induced by intraplantar bradykinin injection. Furthermore, intervertebral foramen injection of TRPC3/6 antagonist relieved inflammation‐induced mechanical pain hypersensitivity. Taken together, these results point to the conclusion that TRPC3/6, highly expressed in nociceptors, is coupled to B2/B1, a pro‐inflammatory GPCR^[^ ^84^, ^85^ ^]^ and trigger downstream BDNF production and secretion, which in turn potentiates spinal synaptic transmission through TRPC3/6. In consequence, this feed‐forward regulatory network between TRPC3/6 and BDNF in nociceptors may underlie the mechanical pain hypersensitivity associated with peripheral inflammation.

In summary, this study sheds light on the functional role of TRPC3/6 in the pain transmission and sensitization caused by peripheral inflammation. Further mechanistic analysis reveals that nociceptor‐localized TRPC3/6 is well placed to orchestrate nociceptor hyperexcitability and spinal synaptic potentiation via presynaptic mechanisms, thereby providing a basis for opening a novel therapeutic target to treat patients suffering from common cause of mechanical allodynia with least side effects. This promising possibility in clinical translation in pain treatment is strongly supported by the highly expressed TRPC3 and TRPC6 in human nociceptive DRG neurons and significant upregulation following inflammation as well as marked alleviation of abnormal nociceptor hyperexcitability by pharmacologically antagonizing TRPC3 and TRPC6 under pathological states. Given the fact that DRG is located at the first station along ascending nociceptive pathway and lies outside of the blood‐brain barrier,^[^ ^58^ ^]^ emerging attention has been paid in target‐rich peripheral nociceptors for drug discovery in pain treatment for fulfillment of optimal analgesic efficacy with least side effects.^[^ ^86^, ^87^ ^]^ Taken together, targeting TRPC3/6 in nociceptors may pave a new way for this direction.

## 4 Experimental Section

### Animals

A double knockout mouse line TRPC3/6^−/−^ (TRPC3/6 DKO mice) were generated by crossing two mouse lines TRPC3^−/−^, TRPC6^−/−^. Every single knockout mouse line had been back crossed to C57Bl6 stain for at least eight generations. Experiments were conducted using adult mice (6‐8 week‐old) of both sexes, with identical number of females and males in each group to minimize the possible gender difference. Mice were housed up to 5 per cage and maintained on a 12 h light/dark cycle with ad libitum access to food and water. All animal procedures were reviewed and approved by Institutional Animal Care and Use Committee of the Fourth Military Medical University (FMMU, IACUC‐20160960). All testing was done in a double‐blinded manner. The experimenter was blind to treatment throughout. See the details for mouse lines in Table S1 (Supporting Information).

### Animal Pain Models

Unilateral injection of complete Freund's adjuvant (20 µl) (CFA) was performed into the intraplantar surface of mouse hindpaw. Various tests including western blotting, patch clamp recordings were conducted at 24 h post CFA injection. The intraplantar bradykinin test were performed by injection of bradykinin (0.5 mM, 20 µl) into the intraplantar surface of mouse hindpaw.

### Human DRG Tissues

In a subset of clinical specimen experiments, we used human DRG tissues harvested from brachial plexus avulsion (BPA) patients who admitted to Orthopedics department of Xijing Hospital, the first affiliated hospital of the Fourth Military Medical University. Most BPA patients suffered from severe neuropathic pain. A series of reconstruction surgical strategy is needed to treat BPA, which includes partial resection of the avulsed nerve roots (including DRG cell bodies) and the following nerve transposition for the recovery of motor function of the affected limb. As a human DRG specimen, the intraoperative resected avulsed roots (including DRG cell bodies) of brachial plexus were collected and subjected to further biochemical analysis and patch‐clamp recording. Healthy human DRG tissues were donated by the tissue bank of the orthopedics department. The study was approved by the Ethics Committee of Xijing Hospital, Fourth Military Medical University (KY20222228‐F‐1). Patients involved in this study have signed the informed consent form, and all specimens were handled in an anonymized way according to ethical and legal standards.

### Behavioral Analyses

All mice were allocated randomly in experimental group. Before behavioral tests, mice were allowed to acclimatize to the behavioral testing room for 3 days and in individual test compartments for at least 1 h. All testing was conducted in a blinded manner.

### Mechanical Allodynia

Mechanical withdrawal threshold testing (von Frey test) was conducted using calibrated von Frey filaments ranging from 0.008 – 2.0 g (0.008, 0.02, 0.04, 0.07, 0.16, 0.40, 0.60, 1.0, 1.4, and 2.0 g) on an elevated mesh‐bottomed platform (Danmic Global, CA, USA). Beginning with 0.008 g, filaments were applied to the lateral plantar surface with just enough force to bend the fiber and held for 1 s. A “positive response” to the von Frey mechanical stimuli was defined as an abrupt foot lift upon application of von Frey hairs. Each filament was applied 10 times, and the paw withdrawal response frequency was recorded. The force of a particular filament required to elicit 50% frequency of paw withdrawal was expressed as the mechanical threshold.

### Quantitative RT‐PCR

Mouse L3‐L4 DRGs and spinal dorsal horn tissue from L3‐L4 segments were harvested, shock‐frozen on dry ice. Total RNA from DRGs and spinal dorsal horn was extracted using TRIzol Reagent (Invitrogen) in accordance with the manufacturer's protocol. RNA extracts were reverse‐transcribed by PrimeScript RT Master Mix (TaKaRa, #RR036A, Dalian, China) at 37 °C for 15 min and 85 °C for 5 s. Targets were then amplified in triplicate by quantitative RT‐PCR using TB Premix Ex Taq (#RR820A, TaKaRa) on a StepOnePlus Real‐Time PCR System (Applied Biosystems, Foster City, CA, USA), normalized to GAPDH, and quantified by the comparative cycle threshold method (2^ΔΔCT^). Primer sequences was used as follows. TRPC3 forward: TTCATGTTCGGTGCTCGTGG, reverse: TGACATTGAGCGTGCGAGAC; TRPC6 forward: CCTTGCTGTTGCCATTGGATTGC, reverse: GTGAAGGAGGCTGCGTGTGC; GAPDH forward: TGTGTCCGTCGTGGATCTGA; reverse: TTGCTGTTGAAGTCGCAGGAG. See the list of reagents used in Table S1 (Supporting Information).

### Western Blotting

Mouse L3‐L4 DRGs and spinal dorsal horn tissue from L3‐L4 segments as well as human DRG and its associated spinal root were collected and lysed using RIPA lysis buffer (50 mM Tris‐HCl, pH 7.4, 150 mM NaCl, 5 mM EDTA, 1% Triton X‐100, 0.5% sodium deoxycholate, 0.1% SDS) and standard protease inhibitors by sonication on ice, and centrifuged at 12 000 rpm for 10 min. Total protein was measured by BCA Protein Assay Kit (#23 225, Thermo Scientific, Waltham, MA, USA), mixed with 5× SDS‐PAGE loading buffer (#CW0027, CWBIO, Beijing, China), and heated at 100 °C for 10 min. Protein samples were resolved by SDS‐PAGE, and immunoblotted with corresponding antibodies (Table S1, Supporting Information). Proteins were visualized with enhanced chemiluminescence detection methods. The scanned images were quantified using ImageJ software. Specific bands for each protein were normalized to its respective β‐actin loading control. See the list of antibodies used in Table S1 (Supporting Information).

### Immunofluorescence Labelling

Mice were anesthetized with pentasorbital sodium and transcardially perfused with saline followed by 4% paraformaldehyde, and DRG and spinal cord tissues were removed. Mouse DRG and spinal cord tissues as well as human DRG tissues were postfixed overnight in 4% paraformaldehyde, and cryoprotected in 30% sucrose at 4 °C until the tissue sank to the bottom of the container. DRG sections (16 µm in thickness) and transverse spinal cord sections (16 µm) were cut on a cryostat and were immunostained with appropriate antibodies (Table S1, Supporting Information). Briefly, sections were incubated with a solution containing 0.3% Triton X‐100 and 5% bovine serum albumin (BSA) for 1 h at room temperature. Sections were then incubated with primary antibodies over night at 4 °C. After three washes with PBST, the secondary antibodies were applied for 2 h at room temperature. All images were captured with an Olympus laser scanning confocal microscope equipped with argon and krypton lasers (Olympus FV1200, Ishikawa, Japan). DRG and spinal neurons were visualized with a 10X (0.4 NA), 20X (0.75 NA), 40X (0.95 NA) objective using the DAPI, 488, 594 and 647 channels and filters. See the list of antibodies used in Table S1 (Supporting Information).

### Fluorescence In Situ Hybridization (FISH)

The methods for fluorescence in situ hybridization have been described elsewhere.^[^ ^88^ ^]^ In brief, a complementary DNA fragment of TRPC3 (GenBank accession NM_01 9510.3, TRPC3 nucleotides 149–2881), TRPC6 (GenBank accession NM_0 012 82086.1, TRPC6 nucleotides 291–2849) was cloned and riboprobes were synthesized by digoxigenin labelling. The adult mice were deeply anesthetized and perfused with 0.01 M diethylpyrocarbonate‐treated phosphate‐buffered saline (DEPC‐ PBS, pH 7.4) followed by 4% formaldehyde in 0.1 M phosphate buffer (PB, pH 7.4). The DRG and spinal cord tissues were removed and sectioned on a cryostat at 16 µm thickness. The sections were then acetylated for 10 min and pre‐incubated for 1 h at 60 °C and then hybridized for 18 h at 60 °C with 1 µg mL^−1^ sense or antisense DIG‐ labelled TRPC3 or TRPC6 riboprobes in hybridization buffer. After that, the hybridized sections were incubated overnight at room temperature in a mixture of peroxidase‐ conjugated anti‐DIG antibody (1:100) and one of the following antibodies: Dy Light 594 Griffonia Simplicifolia Lectin‐Isolectin B4 (1:300), goat anti‐CGRP (1:300), or mouse anti‐NF200 (1:300). The TRPC3 and TRPC6 probe hybridization signal was amplified by using the biotinylated tyramine‐glucose oxidase. After washing, sections were incubated with a mixture of 2 µg mL^−1^ Alexa Fluor 488‐conjugated streptavidin to visualize the riboprobes and 4 µg mL^−1^ Alexa Fluor 594‐conjugated anti‐ goat, or anti‐ mouse IgG to visualize other molecules for 3–4 h at room temperature. See the list of reagents used in Table S1 (Supporting Information).

### ELISA Assay

Mouse L3‐L4 DRGs were collected at different time points after intraplantar injection of CFA. The tissue samples were homogenized in a lysis buffer containing protease and phosphatase inhibitors. Protein concentrations were determined by BCA Protein Assay (Pierce). Bradykinin (ab136936) ELISA kit was purchased from Abcam. Detection of bradykinin was performed in accordance with the manufacturer's protocol. In brief, 100 µl standard product and samples were added into the appropriate wells separately, and then incubated at 37 °C for 90 min. The liquid was removed and 100 µl Biotinylated Detection antibody working solution was added into each well, incubating at 37 °C for 1 h. After wash, 100 µl HRP enzyme conjugate working solution was added for incubation at 37 °C for 30 min followed by substrate solution (TMB) (90 µl) incubation at 37 °C for 15 min away from light. The reaction was terminated by adding 50 µl stop solution, and read the O.D. absorbance at 450 nm immediately. The standard curve was drawn, and the concentration calculated for each well. See the list of reagents used in Table S1 (Supporting Information).

### RNAscope Assay

RNAscope in situ hybridization multiplex version 2 on fresh DRG frozen tissue was performed as instructed by Advanced Cell Diagnostics (ACD), as previously described.^[^ ^89^ ^]^ In brief, slides with DRG sections were removed from the cryostat and immediately transferred to cold 10% formalin for 15 min. The tissues were then dehydrated with graded ethanol at room temperature. A protease IV digestion was performed for 30 min for all experiments. TRPC3 and TRPC6 probes were designed and synthesized by ACD. The DRG sections were incubated with TRPC3 and TRPC6 probes and then washed with 1X RNAscope wash buffer. The tissues were incubated in 1:5000 DAPI for 1 min before being washed, air‐dried, and coverslipped with Prolong Gold Antifade mounting medium. Images (×20) were acquired on an FV3000 confocal microscope (Olympus). The acquisition parameters were set based on guidelines for the FV3000 provided by Olympus. See the list of reagents used in Table S1 (Supporting Information). All tissues were checked for RNA quality by using a positive control probe cocktail (ACD Cat. 320 861), which contains probes for high‐, medium‐ and low‐expressing mRNAs that are present in all cells (ubiquitin C>peptidyl‐prolyl cis–trans isomerase B> DNA‐directed RNA polymerase II subunit RPB1). All tissues showed robust signal for all three positive control probes. A negative control probe (ACD Cat. 320 871) against the bacterial DapB gene (ACD) was used to check for non‐specific/background label.

### Intact Whole‐Mount DRG Preparations and Whole‐Cell Patch Clamp Recording

As described previously,^[^ ^69^ ^]^ L3/L4 DRGs were carefully removed and placed into artificial cerebrospinal fluid (ACSF). After removing the connective tissue, the ganglia were digested with a mixture of 0.4 mg ml^−1^ trypsin and 1.0 mg ml^−1^ type‐A collagenase (Sigma) for 40 min at 37 °C. The intact ganglia were then incubated in ACSF oxygenated with 95% O_2_ and 5% CO_2_ at 28 °C for at least 1 h before transferring them to the recording chamber. The ACSF contained (in mM): NaCl, 125; KCl, 2.5; NaH_2_PO_4_, 1.2; MgCl_2_, 1.0;CaCl_2_, 2.0; NaHCO_3_, 26 and glucose 10. The pipette solution for DRG recording contained the following (in mM): K‐gluconate, 126; NaCl, 10; MgCl_2_, 1; EGTA, 10; NaATP, 2 and MgGTP, 0.1, adjusted to pH 7.4 with KOH and osmolarity 295–300 mOsm. DRG neurons were visualized with a 40X water‐immersion objective using a microscope (BX51WI; Olympus, Tokyo, Japan) equipped with infrared differential interference contrast optics. Whole‐cell current and voltage recordings were made from DRG neurons with patch‐pipette electrodes having a resistance of 5–8 MΩ. Signals were acquired using an Axopatch 700B amplifier (Molecular Devices Corporation), low‐pass filtered at 5 kHz, sampled at 10 kHz and data analyzed using pCLAMP10.0 software. For membrane properties analysis, membrane potential was held at ‐70 mV under current‐clamp mode and pipette capacitance compensation was performed. Depolarizing current steps (500 ms in duration and 20 pA increments) were used to detect the AP. The AP threshold was determined by differentiating the AP waveform and setting a rising rate of 10 mV ms^−1^ as the AP inflection point. The AP amplitude was measured from the equipotential point of the threshold to the spike peak. The AP duration was measured at the half‐width of the spike.

DRG neurons were patched in the presence of neurobiotin (1%, Vectorlabs) in the intrapipette solution. After recording, DRG containing neurobiotin‐filled cells was fixed with 4% paraformaldehyde and was subsequently immunostained with Alexa Fluor 350‐conjugated streptavidin (Molecular probe; dilution 1:600) and double‐stained with anti‐CGRP antibody (Abcam, 1:500) or isolectin B4 (Vector, 1:200) using standard protocol as described above. See the list of reagents used in Table S1 (Supporting Information).

### DiI Labelling of Spinal Projection Neurons In Vivo and Spinal Slice Patch Clamp Recordings

Mice were placed in a stereotaxic apparatus and received a single injection of 100 nl of 2.5% DiI into the right PAG according to coordinates derived from the atlas of Paxinos and Watson. After a 2‐ to 3‐day survival period, transverse 350–450 µm thick spinal cord slices with dorsal roots attached were obtained. The slices were stored in an incubation solution at room temperature (in mM: NaCl, 95; KCl, 1.8; KH_2_PO_4_, 1.2; CaCl_2_, 0.5; MgSO_4_, 7; NaHCO_3_, 26; glucose, 15; sucrose, 50; oxygenated with 95% O_2_, 5% CO_2_; pH 7.4). A slice was then transferred into a recording chamber and superfused with oxygenated recording solution at 3 ml min^−1^ at room temperature. The recording solution was identical to the incubation solution except for (in mM): NaCl, 127; CaCl_2_, 2.4; MgSO_4_, 1.3 and sucrose 0. All injection sites were confirmed histologically. To detect lamina, I projection neurons which were labelled by DiI from the injection sites of PAG (as described above), slices were illuminated with a monochromator, and visualized with an upright fluorescence Olympus BX51WI microscope (Olympus, Japan), equipped with Dodt‐infrared optics using a 40X, 0.80 NA water‐immersion objective and a cooled CCD camera (TILL Photonics, Gräfelfing, Germany).

Standard whole‐cell patch clamp recordings were performed with glass pipettes having a resistance of 4–6 MΩ in lamina I of spinal dorsal horn. The pipette solution consisted of (in mM): K‐gluconate, 135; KCl, 5; CaCl_2_, 0.5; MgCl_2_, 2; EGTA, 5; HEPES, 5 and Mg‐ATP, 5, pH 7.4 with KOH, measured osmolarity 300 mOsm. QX‐314 (5 mM) was added to the pipette solution to prevent discharge of action potentials (Aps). The electrophysiological properties of the recorded neurons were acquired with an Axon700B amplifier (Molecular Devices Corporation) and pCLAMP10.0 software. Signals were low‐pass filtered at 5 kHz, sampled at 10 kHz and analyzed offline. The membrane potential was held at ‐70 mV. Evoked excitatory postsynaptic currents (eEPSCs) from labelled neurons in lamina I were recorded by stimulating dorsal root with a suction electrode in the presence of inhibitory synaptic transmission antagonists, gabazine (10 µM) and strychnine (1 µM). Test pulses of 0.1 ms with intensity of 3 mA were given at 30 s intervals. Aδ‐fiber or C‐fiber evoked EPSCs (eEPSCs) were distinguished on the basis of the conduction velocity (CV) of afferent fibers (Aδ: 2–13 m s^−1^; C: <0.8 m s^−1^; calculated from the latency of EPSC from a stimulus artifact and the length of dorsal root), as described previously.^[^ ^32^, ^90^ ^]^ Aδ‐fiber responses were considered as monosynaptic in origin when the latency remained constant and there was no failure during stimulation at 20 Hz for 1s, while C‐fiber responses were considered as monosynaptic in origin when the latency remained constant and failures did not occur during repetitive stimulation at 2 Hz for 10 s.^[^ ^32^, ^90^ ^]^ Spinal LTP was induced by low frequency stimulation (LFS, 2 Hz for 2 min) delivering to dorsal root at a holding potential of ‐70 mV with same intensity as test stimulation. Synaptic strength was quantified by the peak amplitudes of EPSCs. The mean amplitude of 10 EPSCs evoked by test stimuli prior to conditioning stimulation served as a control. Significant changes from control were assessed by measuring the peak amplitudes of five consecutive EPSCs every 5 min after conditioning stimulation.

**mEPSCs analysis** In a subset of experiments, miniature EPSCs (mEPSCs) were recorded in the presence of TTX (0.5 µM), gabazine (50 µM) and strychnine (10 µM) at a holding potential of ‐70 mV.

**Paired‐pulse ratio (PPR) analysis** In a subset of experiments, paired‐pulse stimuli with an inter‐stimulus interval of 110 ms (0.1 ms pulse duration, 3 mA intensity, every 30 s) were applied to dorsal root. Paired‐pulse ratio of AMPARs‐mediated eEPSCs was recorded at a holding potential of ‐70 mV and calculated as the amplitude of the second eEPSCs divided by that of the first eEPSCs in a pair. See the list of reagents used in Table S1 (Supporting Information).

### Stereotaxic Surgery

Virus injection in DRG and spinal cord was performed as described previously.^[^ ^32^, ^33^, ^91^, ^92^, ^93^ ^]^ For DRG injection, briefly, 4‐6‐week old mice were anesthetized with isoflurane and two lumbar DRGs exposed by removal of the lateral processes of the vertebrae. The epineurium over the DRG was opened, and the glass pipette with fine tip was inserted into the ganglion, to a depth of 100 µm from the surface of the exposed ganglion. After waiting 2 min to allow sealing of the tissue around the pipette tip, 1.0 µl of AAV2/8 virions expressing DIO‐BDNF‐pHluorin, Loxp‐TRPC3/TRPC6‐shRNA, Loxp‐BDNF‐shRNA were injected into DRGs of SNS‐Cre at a rate of 0.1 µl min^−1^ using microprocessor‐controlled minipump (RWD). For spinal photometry recording, 1.0 µl of AAV2/9‐CaMKIIa‐GCaMP6s was injected into L3‐L4 spinal dorsal horn segment of WT and TRPC3/6 DKO mice. For TRPC3/6 knockdown specifically in nociceptors experiments, mixture of 500 nl of AAV2/8‐U6‐Loxp‐TRPC3 shRNA and 500 nl of AAV2/8‐U6‐Loxp‐TRPC6 shRNA was unilaterally injected into L3/L4 DRGs of SNS‐Cre mice. AAV2/8‐U6‐scrambled shRNA was served as controls. For testing BDNF secretion from spinal nociceptor terminals, 900 nl mixture of a 1:1:1 ratio of AAV2/8‐DIO‐BDNF pHluorin, AAV2/8‐U6‐loxp‐TRPC3 shRNA and AAV2/8‐U6‐loxp‐TRPC6 shRNA was injected into L3/L4 DRGs of SNS‐Cre mice. After viral injection, the pipette was kept in place for 10 min to allow diffusion of the virus. The muscles overlying the spinal cord was carefully sutured and mice allowed to recover at 37 °C warming blanket. Mice were allowed to recover for 4 weeks before commencing behavioral and imaging analysis. At the end of the experiment, mice were perfused as described above and expression of virus was confirmed via fluorescence analysis or combined with western blotting analysis for some viruses. Data were excluded in experiments when the viral injections were in incorrect location. Details of virus information have been provided in Table S1 (Supporting Information). See the list of viruses used in Table S1 (Supporting Information).

### Nociceptor‐Specific Knockdown of TRPC3, TRPC6 or BDNF

Nociceptor‐specific knockdown of TRPC3/6 or BDNF was achieved with the methods described previously.^[^ ^94^ ^]^ Briefly, according to the mouse cDNA sequence of TRPC3, TRPC6 or BDNF, we generated oligonucleotides corresponding to TRPC3‐specific shRNA (sense strand: GCCACTCAAGGTCTAGGATCA; antisense strand: TGATCCTAGACCTTGAGTGGC), TRPC6‐specific shRNA (sense strand: GCAACAGCTCCTGTCCATATG; antisense strand: CATATGGACAGGAGCTGTTGC), BDNF‐specific shRNA (sense strand: GCCCGACTACGCCGGTGCACC; antisense strand: GGTGCACCGGCGTAGTCGGGC), annealed them and cloned them into an AAV2/8 plasmid that designed with a floxed mcherry or enhanced green fluorescent protein (EGFP)‐tagged stop sequence (rAAV2/8‐U6‐Loxp‐CMV‐mcherry‐Loxp‐shRNA TRPC3 (TRPC6), rAAV2/8‐ U6‐Loxp‐CMV‐EGFP‐Loxp‐shRNA BDNF) (Figure 7D). Similarly, a control shRNA (rAAV2/8‐U6‐Loxp‐CMV‐mcherry‐Loxp or rAAV2/8‐U6‐Loxp‐CMV‐EGFP‐Loxp) was cloned and packaged (AAV2/8‐conRNA) to serve as a negative control for potential unspecific effects associated with delivery of shRNA. To accomplish nociceptor‐specific knockdown of TRPC3/6, mixture of 500 nl of Loxp‐TRPC3 shRNA‐expressing AAV2/8 and 500 nl of Loxp‐TRPC6 shRNA‐expressing AAV2/8 were unilaterally injected into L3/L4 DRGs of SNS‐Cre mice as described above. Similarly, BDNF knockdown in nociceptors was achieved by injection of Loxp‐ BDNF shRNA‐expressing AAV2/8 (1.0 µl) into L3/L4 DRGs of SNS‐Cre mice. AAV2/8‐ conRNA was injected in a similar way as controls. See the list of viruses used in Table S1 (Supporting Information).

### Analysis of BDNF Secretion in Presynaptic Terminals of Nociceptors with BDNF‐ pHluorin

AAV2/8‐DIO‐BDNF‐pHluorin was constructed by inserting BDNF‐pHluorin fragment into AAV2/8‐DIO vector, as shown in detail in Figure 7D and described previously.^[^ ^33^ ^]^ Briefly, for making a custom AAV2/8‐DIO vector, double floxed (floxed with two loxPs and lox2272) gene insertion cassette was inserted into AAV2/8‐EF1a vector through AscI and NheI restriction enzyme sites. Then, PCR‐amplified BDNF‐pHluorin from puc‐BDNF‐pHluorin was inserted into AAV2/8‐DIO vector through NheI and AscI restriction enzyme site to construct AAV2/8‐DIO‐BDNF pHluroin. Sequencing and restriction enzyme reactions were performed to verify the plasmid. Packaging (serotype 8) and purification of AAV2/8‐DIO‐BDNFpHluorin were carried out by BrainVTA as described in Table S1 (Supporting Information). The titre for each virion is above 2.00E+12 vg/mL and with high quality. For testing the role of TRPC3/6 localized in nociceptors in BDNF secretion from spinal nociceptor terminals, 900 nl mixture of a 1:1:1 ratio of AAV2/8‐DIO‐BDNF pHluorin, AAV2/8‐U6‐loxp‐mcherry‐loxp‐TRPC3 shRNA and AAV2/8‐U6‐loxp‐mcherry‐TRPC6 shRNA was injected into L3/L4 DRGs of SNS‐Cre mice. Mice were tested four weeks after injection. At the end of the experiment, mice were perfused as described above and expression of virus was confirmed via fluorescence analysis. See the list of viruses used in Table S1 (Supporting Information).

### Intervertebral Foramen Injection of Drugs In Vivo

Intervertebral foramen delivery of SAR7334 (0.1, 1, 10 µM) was performed as described previously.^[^ ^95^ ^]^ Briefly, the bilateral iliac spines were used to locate the L3 and L4 vertebrae of mice. The 26‐gauge needle mated to a Hamilton syringe (Hamilton, Reno, NV) was inserted at a 45° angle at the intersection of the lower edge of the ipsilateral L3 and L4 vertebrae and the paravertebral line, infiltrating the intervertebral foramen with 50 µl SAR7334 (0.1, 1, 10 µM). There was a sense of restriction when the needle entered the transverse foramen, and the paw retraction reaction of mice was the sign of the needle entering the transverse foramen. See the list of reagents used in Table S1 (Supporting Information).

### Fiber Photometry Recording

Fiber photometry was used to record calcium‐dependent activity dynamics with the commercialized fiber photometry system (ThinkerTech, Beijing, China) as described previously.^[^ ^96^, ^97^ ^]^ An optical fiber was implanted into the surface of spinal dorsal horn of mice. To excite GCaMP6s, a 473‐nm LED (Cree XP‐E LED, Shenzhen, China) was reflected off a dichroic mirror (MD498, Thorlabs, Newtown, NJ, USA) that was focused by a × 20 objective lens (0.4 NA; Olympus, Ishikawa, Japan) and coupled to an optical commutator (Doris Lenses). An optical fiber (230 µm OD, 0.37 NA) guided the light between the commutator and the implanted optical fiber. The laser power at the tip of the optical fiber was adjusted to 0.01–0.02 mW to decrease laser bleaching. Fluorescence was bandpass‐filtered (MF525‐39, Thorlabs), and an amplifier was used to convert the CMOS (DCC3240M, Thorlabs) current output to signals, which was further filtered through a low‐pass filter (40 Hz cut off, ThinkerTech). The analog voltage signals were digitalized at 50 Hz and recorded by the multi‐channel fiber photometry recording system (ThinkerTech). Probing of the skin with 2 g von Frey filaments at the hindpaw ipsilateral to optical fiber implantation was used as a search stimulus to identify the receptive field of the GCaMP6s‐expressing dorsal horn neurons to be activated with optical fiber. Subsequently, GCaMP6s fluorescence signals were recorded in response to a variety of cutaneous mechanical stimuli as well as electrical stimuli applied to the receptive field. The forms of mechanical and electrical stimuli used for quantification include: 1) different forces of von Frey filaments, 2) innocuous brushing at a frequency of 2 times/s with a hairy paint brush, 3) pressure exerted by picking up a fold of skin with a flattened alligator clip, 4) noxious stimulus exerted by pinching a fold of skin with a small, serrated clip; 5) electrical stimulation at C‐fiber intensity (3 mA, 500 µm in duration).

### Pre‐Embedding Immunogold‐Silver Cytochemistry

As previously described,^[^ ^98^ ^]^ animals were anesthetized with pentobarbital sodium and transcardially perfused with 4% paraformaldehyde and 0.05% glutaraldehyde. Vibrotome sections (50 µm) of spinal cord were cut. TRPC3 or TRPC6 was detected by the immunogold‐silver staining method and CGRP by the immunoperoxidase method. Briefly, sections were incubated in the primary antibodies of rabbit anti‐TRPC3 (Alomone, 1:200) and goat anti‐CGRP (Abcam, 1:500) or rabbit anti‐TRPC6 (Alomone, 1:200) and goat anti‐CGRP (Abcam, 1:500). The secondary antibodies were a mixture of anti‐rabbit IgG conjugated to 1.4 nm gold particles (Nanoprobes, Stony Brook, NY, 1:100) and horseradish peroxidase‐conjugated anti‐goat IgG (1:200 dilution). Silver enhancement was performed with HQ Silver Kit (Nanoprobes) for visualization of TRPC3 or TRPC6 immunoreactivity. They were then visualized by the glucose oxidase‐3, 3′‐diaminobenzidine method for CGRP immunoreactivity.

Immuno‐labeled sections were fixed with 0.5% osmium tetroxide, dehydrated in graded ethanol series, then in propylene oxide, and finally flat‐embedded in Epon 812 between sheets of plastic. Three to four sections containing both TRPC3 (or TRPC6) and CGRP immunoreactivity in the superficial spinal dorsal horn were selected, trimmed and glued onto blank resin stubs. Serial ultrathin sections were cut with an Ultramicrotome (Leica EM UC6, Wetzlar, Germany) using a diamond knife (Diatome, Hatfield, PA) and mounted on formvar‐coated mesh grids (6–8 sections/grid). They were then counterstained with uranyl acetate and lead citrate, and observed under a JEM‐1230 electron microscope (JEOL LTD, Tokyo, Japan) equipped with CCD camera and its application software (832 SC1000, Gatan, Warrendale, PA). Electron micrographs were arranged and contrast‐enhanced by the computer. See the list of reagents used in Table S1 (Supporting Information).

### Statistical Analysis

Data are analyzed in GraphPad Prism version 8.0 (GraphPad Software, La Jolla, CA, USA) and Statistical Program for Social Sciences 21.0 software (SPSS, Inc., Chicago, IL, USA). The normality test was performed by the Shapiro‐Wilk test. The homogeneity of variance test was performed by Levene's test. Data that met these two conditions were analyzed using a two‐tailed unpaired or paired t‐test, one‐way analysis of variance (ANOVA) or repeated‐measures ANOVA followed by Tukey's multiple comparisons test. Data sets that were not normally distributed were analyzed with a nonparametric test (Table S2, Supporting Information). Data were reported as mean ± SEM. *P* value less than 0.05 was considered statistically significant.

## Conflict of Interest

The authors declare no conflict of interest.

## Author Contributions

Z.C.S., W.J.H., Z.W.D., N.L., X.W., F.D.W. and S.B.M. contributed equally to this work. Z.C.S., Z.W.D., X.W., Z.Z.L. and W.N.L. conducted western blotting. N.L., W.J.H. and R.G.X. performed DRG and spinal slice patch clamp recording. Z.C.S., Z.W.D., N. L., X. W. performed animal preparation. S.B.M., Z.C.S., Z.W.D. conducted immunofluorescence staining and fluorescence in situ hybridization. Z.C.S. and Z.C.T. performed behavioral testing. H.X. provided the human DRG samples and performed immunofluorescence staining. R.G.X., F.D.W., S.B.M., W.B.W., Z.C.S. conducted in vivo fiber photometry recording. R.G.X. and Z.C.S. performed BDNF secretion imaging analysis. X.W. and Y.Y.L. conducted ultrastructural experiment. Z.C.S., W.G.C. conducted stereotaxic surgery. Z.C.S., W.J.H, N.L., F.D.W., Z.W.D., S.B.M., X.W., R.G.X., Z.C.T., F.W. analyzed the data. L.B., M.F., H.R.T., D.J. provided critical input on study design and interpretation. C.L., R.G.X., S.X.W. designed studies, C.L. wrote the draft manuscript. C.L., R.G.X., Z.Z.L., S.X.W. supervised the experiments and revised the manuscript. All the authors read and approved the final manuscript.

## Supporting information



Supporting Information

Supporting Tables

## Data Availability

The data that support the findings of this study are available from the corresponding author upon reasonable request.
