# Supplementary material for: TRPC3/6 Channels Mediate Mechanical Pain Hypersensitivity via Enhancement of Nociceptor Excitability and of Spinal Synaptic Transmission
Source: Adv Sci (Weinh). 2024 Sep 28;11(44):2404342. doi: 10.1002/advs.202404342 (PMC11600220; doi:10.1002/advs.202404342)

**Supplementary Figure legends**

**Fig. S1 Motor-coordination on a rotarod was normal in all knockout mouse lines as compared to WT controls. (A-C)** Comparison of the latency to fall on a rotarod in different knockout mouse lines, e.g. TRPC3 KO (**A**), TRPC6 KO (**B**) and TRPC3/6 DKO (**C**) as compared to their corresponding WT littermates, indicating no alteration in motor coordination after single or double knockout of TRPC3 and TRPC6. n = 6 for each genotype. WT, wildtype; 3KO, TRPC3 knockout; 6KO, TRPC6 knockout; DKO, TRPC3/6 double knockout. *P* > 0.05, by two-tailed unpaired t-test. Data are represented as mean ± S.E.M. See Supplementary Table 2 for detailed statistical information.

**Fig. S2 TRPC3/6 DKO mice did not show aberrant developmental defects in sensory neurons.** (**A**) Representative immunofluorescence images (**A-C**) and quantitative summary (**D**) showing the percentages of CGRP-expressing peptidergic neurons, IB4-labelled non-peptidergic DRG neurons as well as NF200-positive DRG neurons were not altered by deletion of TRPC3 and TRPC6. *P* > 0.05 by two-tailed unpaired t-test. Scale bar: 100 μm for the left three columns and 50 μm for the right magnified column in (**A-C**). (**E, F**) TRPC3/6 DKO and WT mice showed similar patterns targeting of nociceptors in the spinal cord (E, F) and the skin (G) as shown by binding to IB4 and immunoreactivity for CGRP. n = 5, *P* > 0.05 by two-tailed unpaired t-test. Scale bar: 100 μm in (**E**) and (**G**). (**H, I**) Deficiency of TRPC3/6 did not induced spinal neuronal loss as well, as revealed by NeuN staining. n = 10, *P* > 0.05 by two-tailed unpaired t-test. Scale bar: 100 μm in the left three columns and 50 μm in the right magnified column in (**H**). Data are represented as mean ± S.E.M. See Supplementary Table 2 for detailed statistical information.

**Fig. S3 The specificity of TRPC3 and TRPC6 riboprobe was confirmed in DRG and spinal cord sections using TRPC3/6 DKO mice.** (A, B) No specific signals were seen for TRPC3 and TRPC6 in DRG sections (A) and spinal cord sections (B) from TRPC3/6 DKO mice.

**Fig. S4 Ultrastructural preembedding double immunostaining showing TRPC3 and TRPC6 are localized in both presynaptic terminal of nociceptors and postsynaptic spinal neurons.** (**A, C**) Immunogold particles showing that TRPC3 (**A**) and TRPC6 (**C**) immunoreactivity are seen in both presynaptic terminals (T, indicated by red arrows) and postsynaptic dendrites (Den, indicated by green arrows). (**B, D**) Photomicrographs showing double labelling of TRPC3 (**B**, yellow arrows) and TRPC6 (**D**, yellow arrows) in nociceptive terminals that are CGRP-positive in the superficial dorsal horn. n = 3, scale bar: 0.2 μm.

**Fig. S5 RNAscope showing the co-localization of TRPC3 and TRPC6 in DRG neurons.** RNAscope TRPC3 expression (green) overlaid with TRPC6 expression (red) and DAPI (blue). Scale bar: 100 μm.

**Fig. S6 Identification of patch clamp recording from nociceptive DRG neurons.** (**A and B**) Images showing double immunofluorescence staining of CGRP or isolectin B4 with Neurobiotin in the patched DRG neurons. Scale bar: 30 μm.

**Fig. S7 Establishment and verification of BDNF knockdown specifically in nociceptive DRG neurons.** (**A**) Schematic diagram showing the construction of Cre-dependent AAV2/8 expressing shRNA BDNF (AAV2/8-U6-Loxp-CMV-EGFP-Loxp-shRNA BDNF). (**B**) Injection of Loxp-BDNF shRNA-expressing AAV2/8 into SNS-Cre expressing mice enable BDNF shRNA transcription in a nociceptor-specific manner. (**C**) Immunostaining images showing nociceptive-specific knockdown of BDNF via injection of rAAV2/8-U6-Loxp-CMV-EGFP-Loxp-shRNA (BDNF) into L3/L4 DRGs of SNS-Cre mice. Note that EGFP immunoreactivity is mainly absent in small- to medium-diameter DRG neurons (arrow), but remains intact in large-diameter DRG neurons (arrowhead). (**D**) Western blot analysis showing that AAV2/8-shRNA BDNF expressing mice showed a dramatic loss of BDNF as compared to AAV2/8-conRNA expressing mice. Shown are typical example blots in left panels and quantitative summary in right panels (n = 4). ***P* < 0.01 by unpaired *t* test with Welch's correction. Scale bar = 50 μm in (**C**). Data are represented as mean ± S.E.M. See Supplemental Table 2 for detailed statistical information.

**Fig. S8 The effect of TRPC3/6 antagonist, GSK283 on the DRG neuronal hyperexcitability from BPA patients. (A)** Images showing whole-cell patch clamp recordings from typical human small DRG neurons. **(B, C)** Typical traces (**B**) and quantitative summary (**C**) showing that bath application of TRPC3/6 antagonist, GSK283 (10 μM) largely attenuated the firing frequency induced by depolarizing current injection in small DRG neurons from BPA patients. n =3, ***P* < 0.01, by paired *t*-test. Scale bar = 25 μm in (**A**). Data are represented as mean ± S.E.M. See Supplemental Table 2 for detailed statistical information.

**Fig. S1**


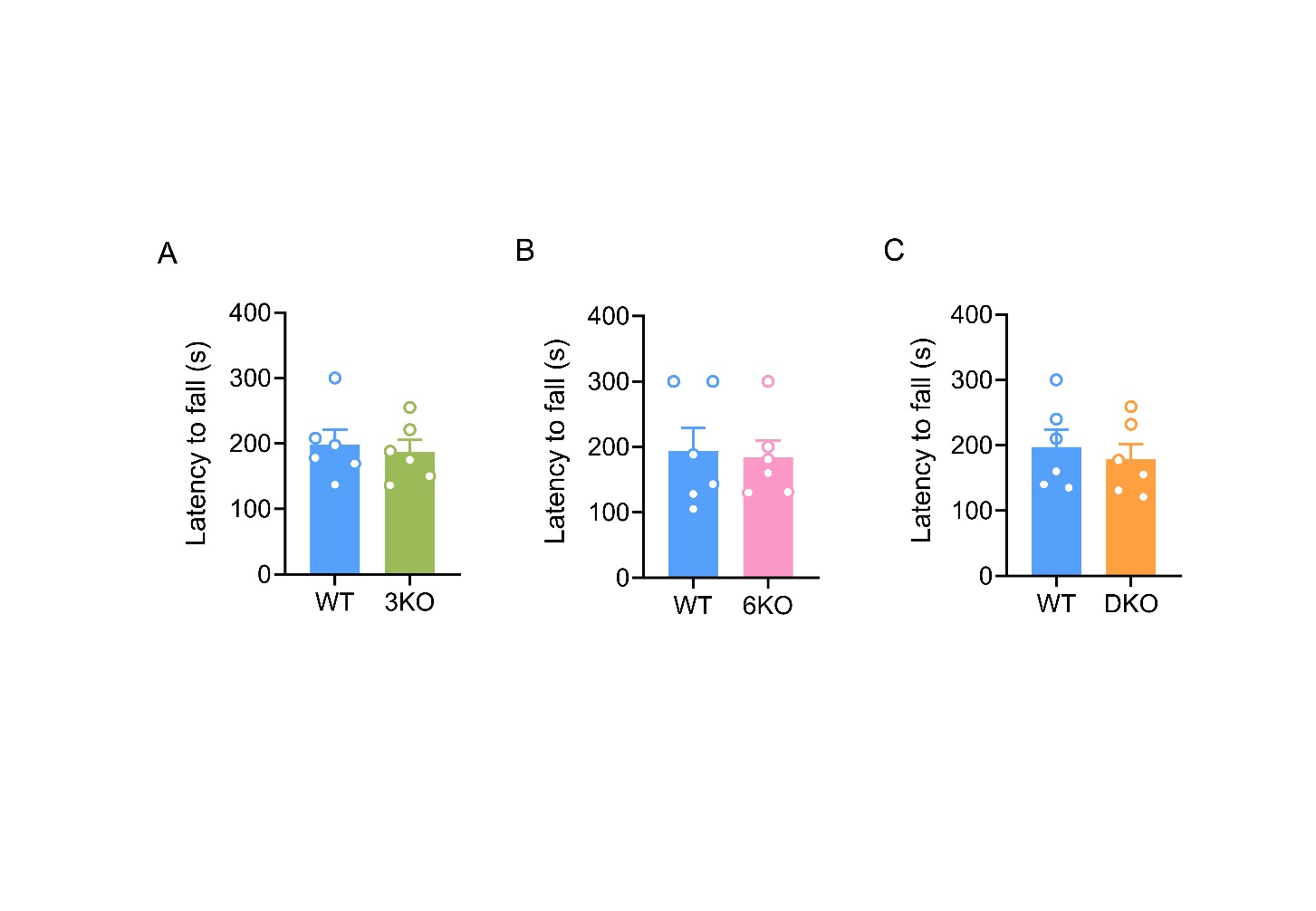


Fig. S2


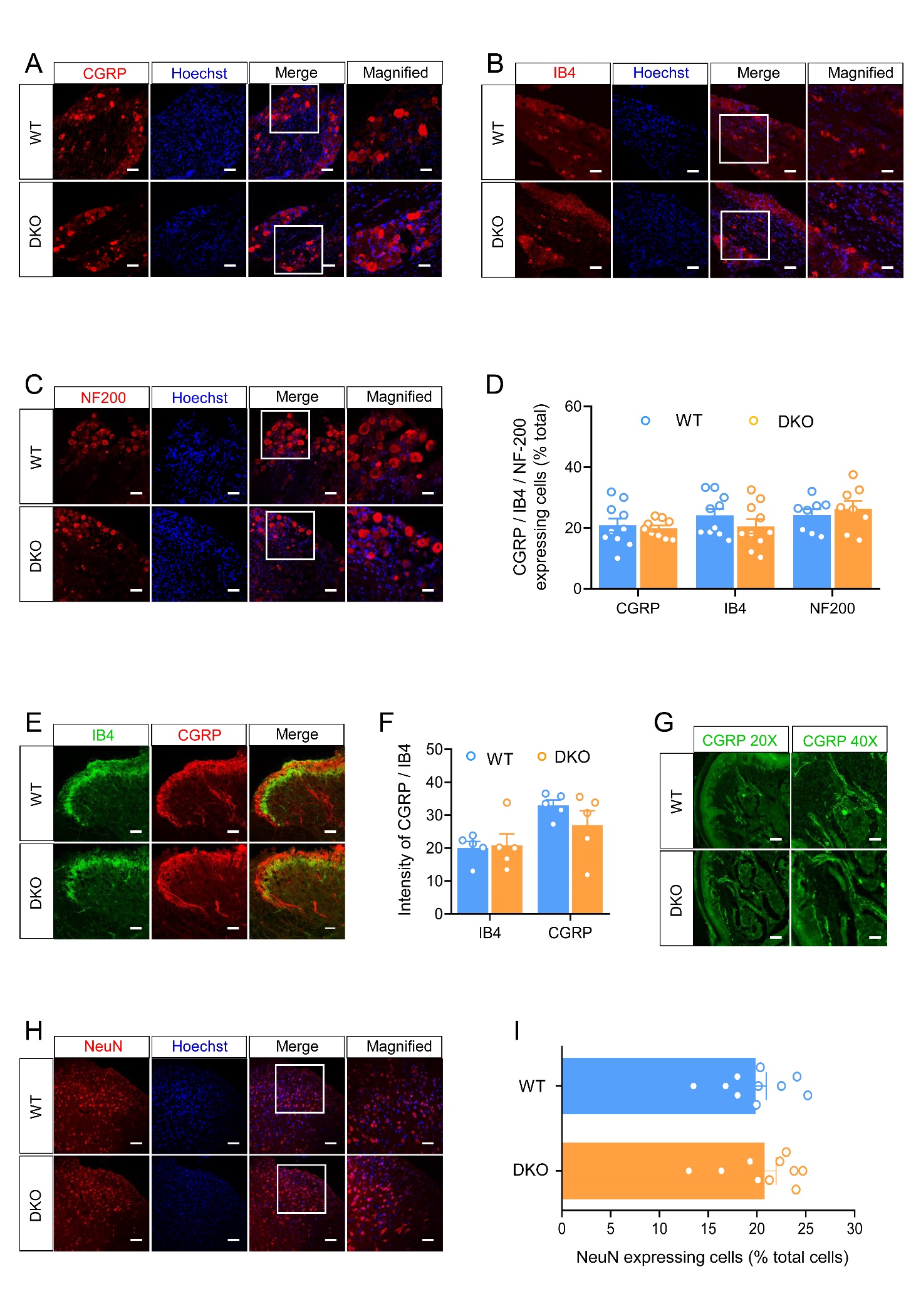


Fig. S3


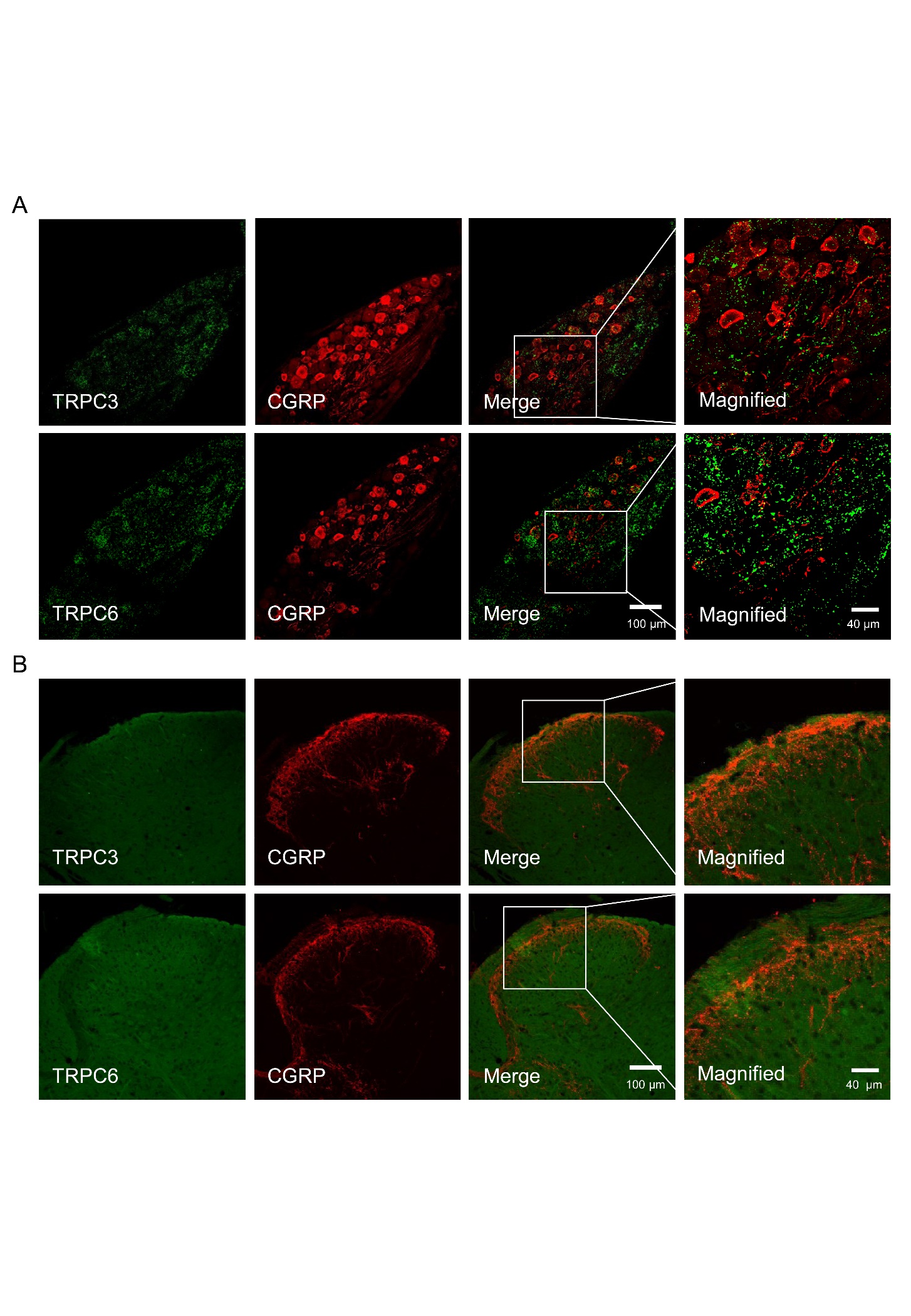


Fig. S4


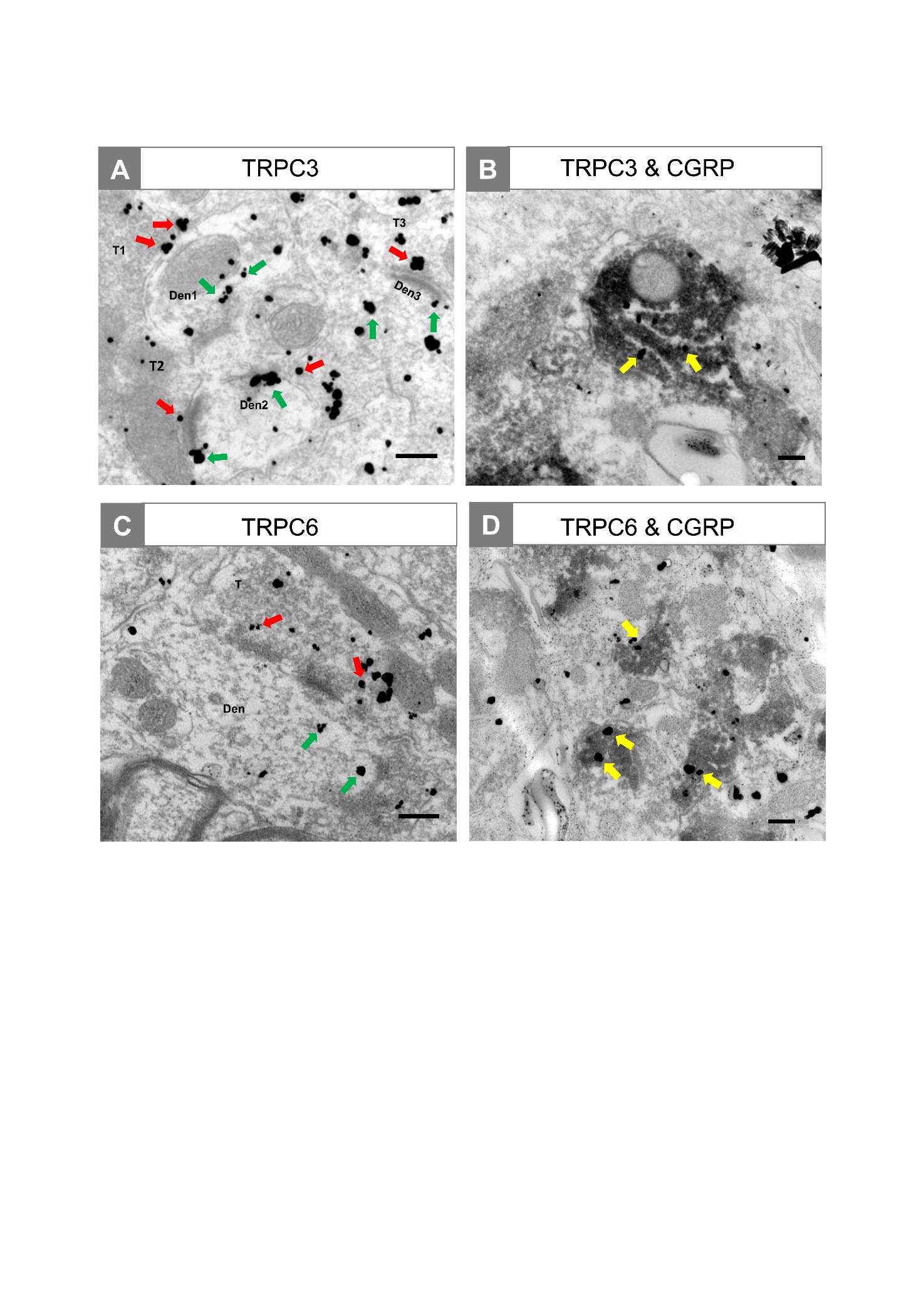


Fig. S5


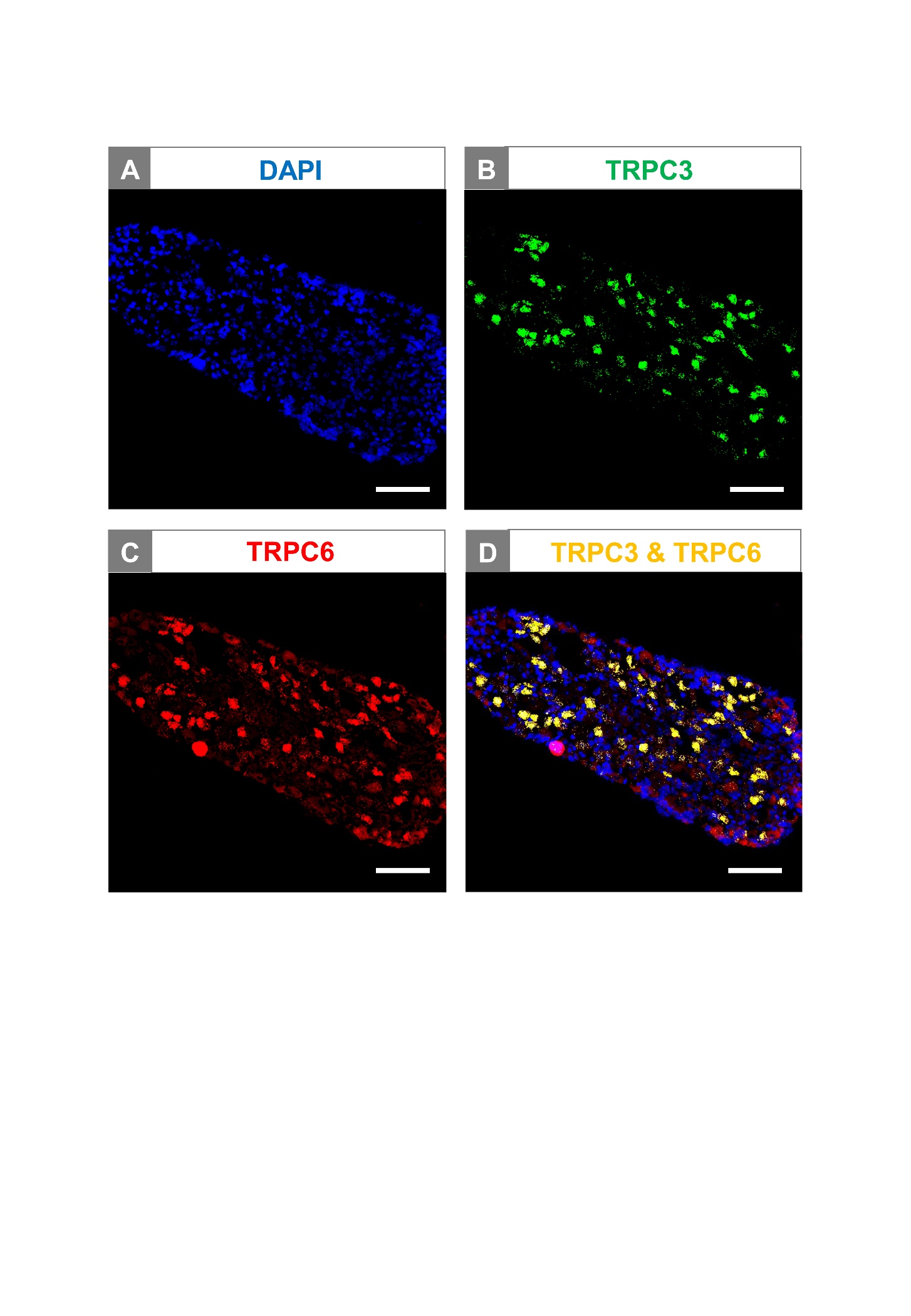


Fig. S6


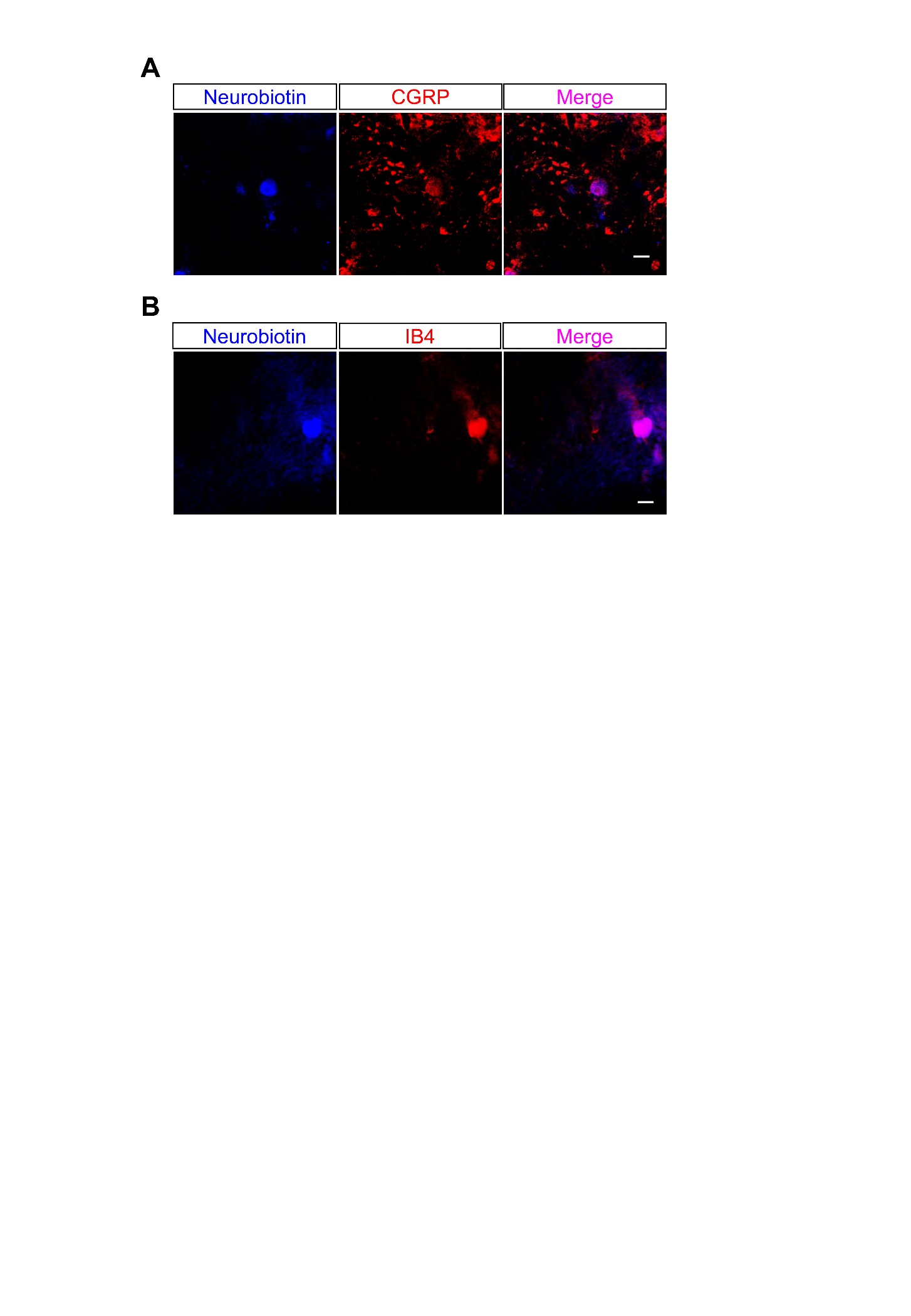


Fig. S7


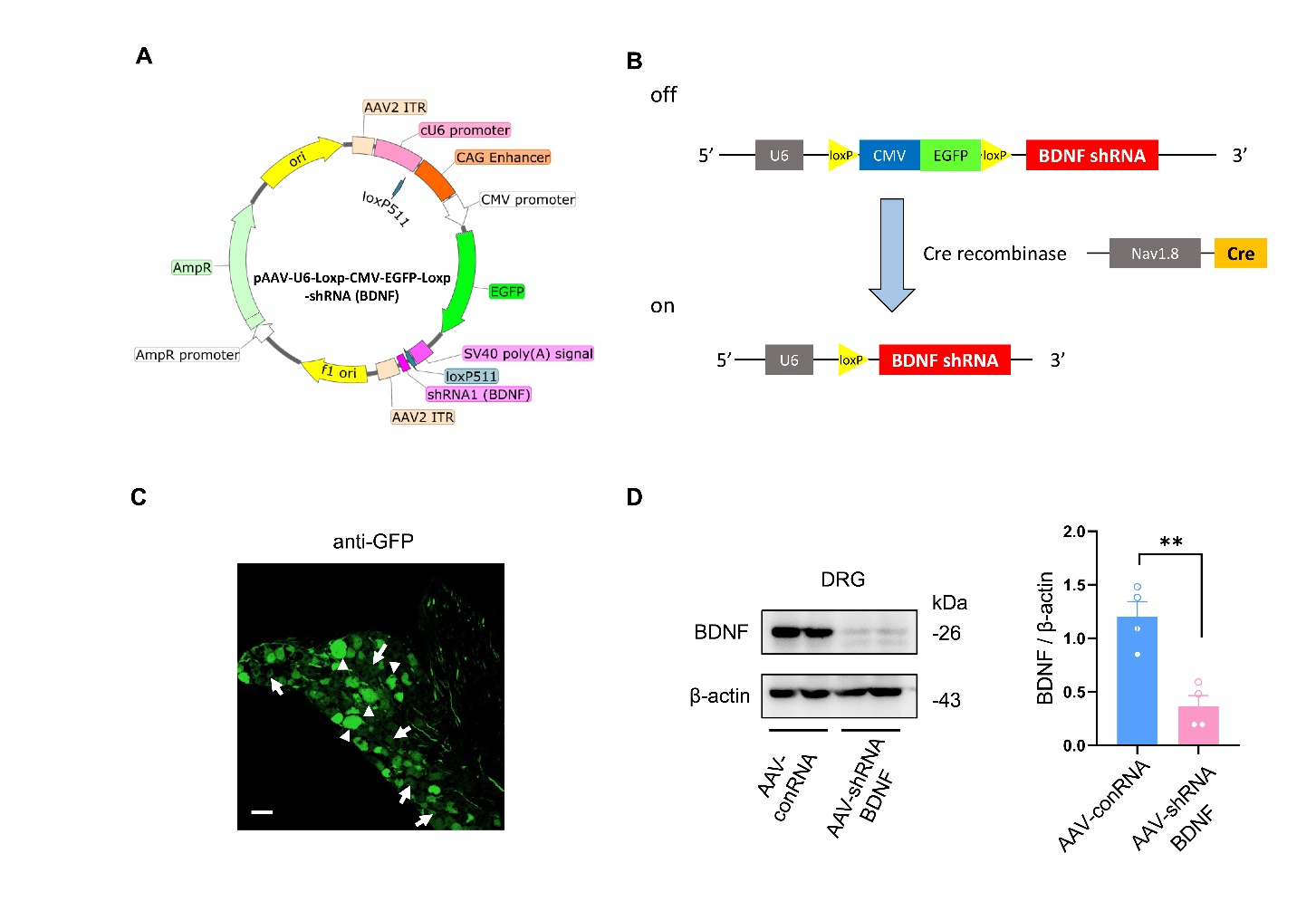


Fig. S8


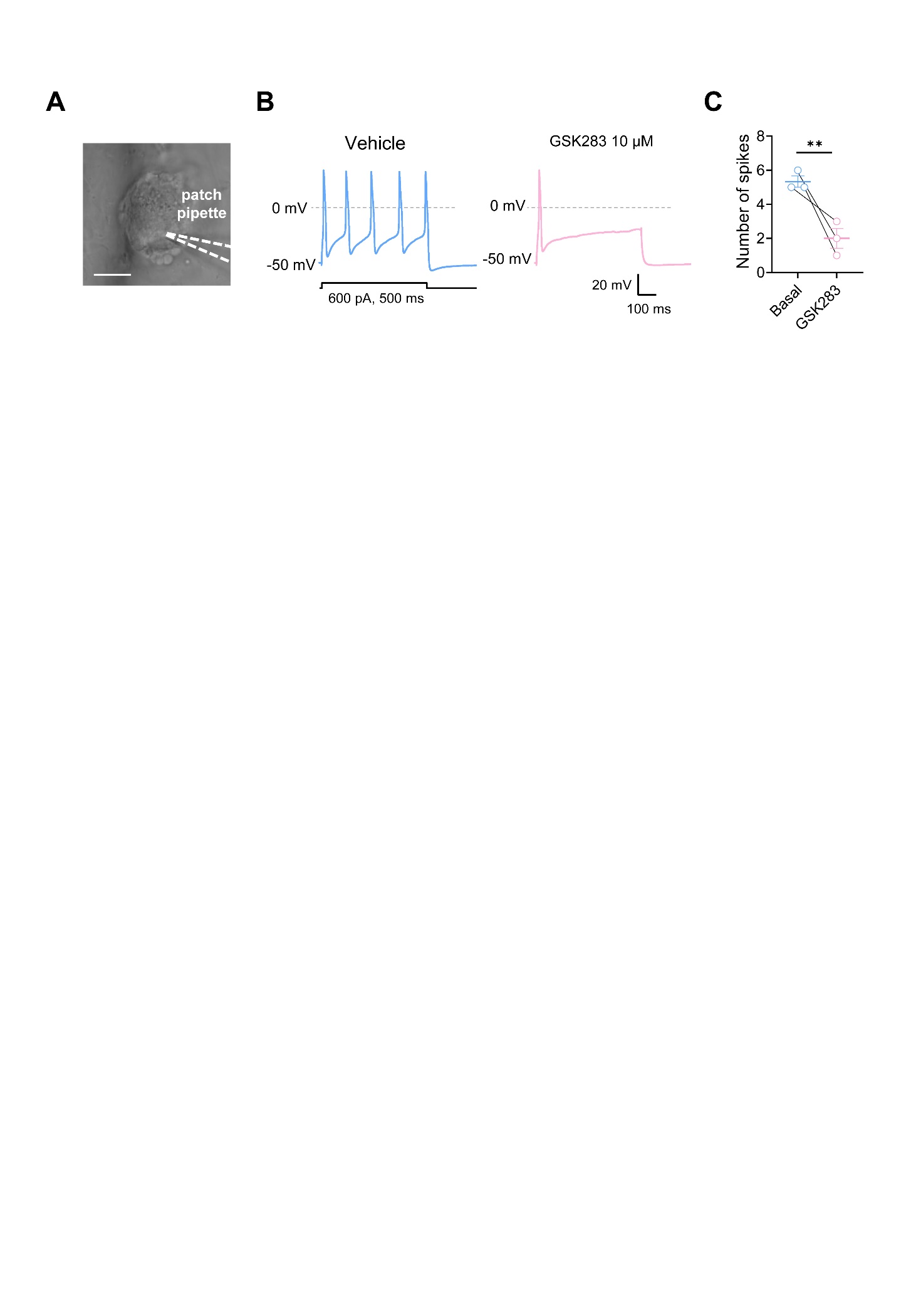

Supplement: Supplementary file 1 — Supporting Information [file ADVS-11-2404342-s001.docx]
