# Supplementary material for: TRPC3/6 Channels Mediate Mechanical Pain Hypersensitivity via Enhancement of Nociceptor Excitability and of Spinal Synaptic Transmission
Source: Adv Sci (Weinh). 2024 Sep 28;11(44):2404342. doi: 10.1002/advs.202404342 (PMC11600220; doi:10.1002/advs.202404342)
Supplement: Supplementary file 2 — Supporting Tables [file ADVS-11-2404342-s002.zip › Supplementary Table 1-1+2-1-R-final.pdf]

Supplemental Table 1

KEY RESOURCES TABLE

REAGENT or	SOURCE	IDENTIFIER	Dilution
Antibodies			
Rabbit anti-TRPC3	Cell Signaling Technology, Boston, MA, USA	77934S	1:1000 (WB)<br>1:200 (IF)
Rabbit anti-TRPC6	Proteintech, Wuhan, China	00026504	1:1000 (WB)<br>1:200 (IF)
Rabbit anti-TRPC3	Alomone Labs, Jerusalem, Israel	ACC-016	1:200 (TEM)
Rabbit anti-TRPC6	Alomone Labs, Jerusalem, Israel	ACC-017	1:200 (TEM)
Rabbit anti- BDNF	Signalway Antibody, Maryland, US	32263	1:1000 (WB)
Mouse anti-P2X3	SANTA CRUZ, Dallas, TX, USA	sc-390572	1:200 (IF)
Mouse anti-β-actin	Proteintech, Wuhan, China	66009-1-AP	1:4000 (WB)
Mouse anti-GAPDH	Proteintech, Wuhan, China	66004-1-Ig	1:4000 (WB)
Rabbit anti-PSD95	Abcam, Cambridgeshire, UK	ab18258	1:1000 (IF)
Rabbit anti-Synaptophysin	Abcam, Cambridgeshire, UK	ab32127	1:200 (IF)
Biotinylated griffonia simplicifolia lectin I, Isolectin B4	Vector, California, USA	B-1205	1:200 (IF)
Goat anti-CGRP	Abcam, Cambridgeshire, UK	ab36001	1:200 (IF)<br>1:500 (TEM)
Mouse anti-NF200	Sigma-Aldrich, St. Louis, MO, USA	N2912	1:200 (IF)
Neurobiotin	Vector, California, USA	SP-1120	1%
Anti-rabbit IgG,HRP-linked Antibody	Cell Signaling Technology, Boston, MA, USA	7074	1:4000 (WB)
Anti-mouse IgG,HRP-linked Antibody	Cell Signaling Technology, Boston, MA, USA	7076	1:4000 (WB)

Donkey anti-Goat IgG (H+L) Highly Cross-Adsorbed Secondary Antibody, Alexa Fluor	Invitrogen, Carlsbad, CA, USA	A21202	1:800 (IF)<br>1:200 (TEM)
Donkey anti-Rabbit IgG (H+L) Cross-Adsorbed Secondary Antibody, Alexa Fluor 594	Invitrogen, Carlsbad, CA, USA	A-1207	1:800 (IF)
Donkey anti-Goat IgG (H+L) Cross-Adsorbed Secondary Antibody, Alexa Fluor 594	Invitrogen, Carlsbad, CA, USA	A11058	1:800 (IF)
Donkey anti-Mouse IgG (H+L) Cross-Adsorbed Secondary Antibody, Alexa Fluor 594	Invitrogen, Carlsbad, CA, USA	A21203	1:800 (IF)
Donkey anti-Mouse IgG (H+L) Cross-Adsorbed Secondary Antibody, Alexa Fluor 647	Jackson, Carlsbad, CA, USA	711-605-151	1:800 (IF)
Bradykinin ELISA kit	Abcam	ab136936	
RNAscope Probe-Mm-TRPC3	Advanced Cell Diagnostics, ACD, California, USA	525201	
RNAscope Probe-Mm-TRPC6-c2	Advanced Cell Diagnostics, ACD, California, USA	442951-c2	
Chemicals, Peptides and Recombinant Proteins			
Strychnine	Sigma-Aldrich, St. Louis, MO, USA	S8753	
Gabazine	Sigma-Aldrich, St. Louis, MO, USA	S106	
Tetrodotoxin (TTX)	TOCRIS, Bristol, UK	43F	
Formalin	Sigma-Aldrich, St. Louis, MO, USA	HT5012	
collagenase	Sigma-Aldrich, St. Louis, MO, USA	C0130	

trypsin	Sigma-Aldrich, St. Louis, MO, USA	T2600000
QX314	Sigma-Aldrich, St. Louis, MO, USA	L5783
ketamine	Sigma-Aldrich, St. Louis, MO, USA	K-002
Capsaicin	TOCRIS, Bristol, UK	0462
BDNF	PeproTech, Cranbury, NJ, USA	450-10
DiI	Molecular Probe, NJ, USA	D3911
SAR7334	MedChemExpress LLC, NJ, USA	HY-15699A
Bradykinin	Abcam, Cambridgeshire, UK	ab120470
Icatibant	MedChemExpress LLC, NJ, USA	HY-17446
SSR 240612	BIOFOUND, Shanghai, China	YzM003514
Complete Freund's adjuvant	Sigma-Aldrich, St. Louis, MO, USA	F5881
Virus Strains		
rAAV-U6-Loxp-CMV-mCherry-SV40 pA-Loxp-shRNA1(TRPC3)	BrainVTA, Wuhan, China	PT-2900
rAAV-U6-Loxp-CMV-mCherry-SV40 pA-Loxp-shRNA3(TRPC6)	BrainVTA, Wuhan, China	PT-2901
rAAV-EF1a-DIO-BDNF-Flag-pHluorin-WPRE-bGH pA	BrainVTA, Wuhan, China	PT-0395
rAAV-U6-Loxp-CMV-EGFP-SV40 pA-Loxp-shRNA1(BDNF)	BrainVTA, Wuhan, China	PT-0521
rAAV-U6-Loxp-CMV-mCherry-SV40 pA-Loxp-shRNA(scramble)	BrainVTA, Wuhan, China	PT-0967
rAAV-U6-Loxp-CMV-EGFP-SV40 pA-Loxp-shRNA(scramble)	BrainVTA, Wuhan, China	PT-0552
rAAV2/9-CaMKIIa-GCaMP6s-WPRE-pA	BrainVTA, Wuhan, China	PT-0110
Experimental models: Organisms/Stains		

Mouse: TRPC3 KO	Dr. Lutz Birnbaumer<br>Dr. Marc Freichel	N./A
Mouse: TRPC6 KO	Dr. Lutz Birnbaumer<br>Dr. Marc Freichel	N./A
Software		
Clampex 9.2	Axon Instrument	N/A
Clampfit 10.6	Axon Instrument	N/A
Olympus Fluoview version 3.1	Olympus	N/A
Other		
Microliter syringe pump	KD Scientific	LEGATO 130
FV1000 laser-scanning confocal microscope	Olympus	N/A
FV3000 laser-scanning confocal microscope	Olympus	N/A
Model 400 heated base<br>Series 8 model 390G	IITC Life Science	Model 400<br>Model 390G
von Frey Hairs	Bioseb	N/A
MultiClamp 700B	Axon Instrument	N/A
Primers		
TRPC3 primer 1 C3LoxF	GCTATGATTAATAGCTCATACCAAGAGATC	
TRPC3 primer 2 C3LoxR	GGTGGAGGTAACACACAGCTAAGCC	
TRPC3 primer 3 C3LoxF2	GAATCCACCTGCTTACAACCATGTG	

TRPC3 primer 4 C3LoxR	GGTGGAGGTAACACACAGCTAAGCC
TRPC6 primer 1<br>TRPC6_01	ACGAGACTAGTGAGACGTGCTACTTCC
TRPC6 primer 2<br>TRPC6_02	GGGTTTAATGTCTGTATCACTAAAGCCTCC
TRPC6 primer 3<br>TRPC6_03	CAGATCATCTCTGAAGGTCTTTATGC
TRPC6 primer 4<br>TRPC6_04	TGTGAATGCTTCATTCTGTTTTGCGCC

Supplemental Table 2

Data	Response variable	Groups	n define as	Animal used	Normality Test (Shapiro-Wilk)	Homogeneity of variance test (Levene's test)	Hypothesis test	Test value	P value	Summary
**Fig1B**	PWMT	WT (n=11), TRPC3 KO (n=11)	mouse	22 mice	Not all subgroups meets p > 0.05		Mann-Whitney U test	U=47.5	0.393	ns
**Fig1C**	PWMT	WT (n=8), TRPC6 KO (n=8)	mouse	16 mice	W=0.849,P=0.093 W=0.869,P=0.146	F=9.675, DFn=1, DFd=14, P=0.008	Two-tailed unpaired separate variance estimation t-test	t=1.691, DF=9.499	0.123	ns
**Fig1D**	PWMT	WT (n=5), TRPC3/6 DKO (n=5)	mouse	10 mice	W=0.860,P=0.227 W=0.970,P=0.875	F<0.0001, DFn=1, DFd=8, P=0.9997	Two-tailed unpaired t-test	t=2.520, DF=8	0.036	*
		WT-Basal (n=13), 3KO-Basal (n=11). WT-CFA1d (n=13), 3KO-CFA1d (n=11). WT-CFA2d (n=13), 3KO-CFA2d (n=11). WT-CFA4d (n=13), 3KO-CFA4d (n=11). WT-CFA7d (n=13), 3KO-CFA7d (n=11).	mouse	24 mice	W=0.883 P=0.079, W=0.946 P=0.597, W=0.884 P=0.082, W=0.882 P=0.109, W=0.930 P=0.340, W=0.911 P=0.249, W=0.898 P=0.126, W=0.942 P=0.549, W=0.927 P=0.310, W=0.909 P=0.237	F=0.257, DFn=1, DFd=22, P=0.617, F=0.071, DFn=1, DFd=22, P=0.793, F=0.008, DFn=1, DFd=22, P=0.930, F=0.710, DFn=1, DFd=22, P=0.409, F=1.046, DFn=1, DFd=22, P=0.318	Two-way repeated-measures ANOVA with post hoc comparison between groups	F=4.026, DF=1	0.057	ns
		WT-Basal vs. 3KO-Basal							0.263	ns

**Fig1F**	Mechanical response threshold	WT-CFA1d vs. 3KO-CFA1d							0.071	ns
		WT-CFA2d vs. 3KO-CFA2d							0.088	ns
		WT-CFA4d vs. 3KO-CFA4d							0.263	ns
		WT-CFA7d vs. 3KO-CFA7d							0.312	ns
		WT-Basal vs. WT-CFA1d							<0.0001	****
		WT-Basal vs. WT-CFA2d							<0.0001	****
		WT-Basal vs. WT-CFA4d							<0.0001	****
		WT-Basal vs. WT-CFA7d							<0.0001	****
		3KO-Basal vs. 3KO-CFA1d							<0.0001	****
		3KO-Basal vs. 3KO-CFA2d							<0.0001	****
		3KO-Basal vs. 3KO-CFA4d							<0.0001	****
		3KO-Basal vs. 3KO-CFA7d							<0.0001	****

Fig1H	Mechanical response threshold	WT-Basal (n=8),<br>6KO-Basal (n=8).<br>WT-CFA1d (n=8),<br>6KO-CFA1d (n=8).<br>WT-CFA2d (n=8),<br>6KO-CFA2d (n=8).<br>WT-CFA4d (n=8),<br>6KO-CFA4d (n=8).<br>WT-CFA7d (n=8),<br>6KO-CFA7d (n=8).	mouse	16 mice	W=0.849 P=0.093,<br>W=0.893 P=0.248,<br>W=0.912 P=0.368,<br>W=0.939 P=0.606,<br>W=0.839 P=0.073,<br>W=0.892 P=0.246,<br>W=0.901 P=0.292,<br>W=0.928 P=0.499,<br>W=0.925 P=0.476,<br>W=0.957 P=0.784	F=2.360,DFn=1,DFd=14,<br>P=0.147,<br>F=0.610,DFn=1,DFd=14,<br>P=0.448,<br>F=0.159,DFn=1,DFd=14,<br>P=0.697,<br>F=1.703,DFn=1,DFd=14,<br>P=0.213,<br>F=3.534,DFn=1,DFd=14,<br>P=0.081	Two-way repeated-measures ANOVA with post hoc comparison	F=38.152,<br>DF=1	<0.0001	****
		WT-Basal vs.<br>6KO-Basal							0.187	ns
		WT-CFA1d vs.<br>6KO-CFA1d							<0.0001	****
		WT-CFA2d vs.<br>6KO-CFA2d							0.00054	***
		WT-CFA4d vs.<br>6KO-CFA4d							0.00142	**
		WT-CFA7d vs.<br>6KO-CFA7d							0.017	*
		WT-Basal vs.<br>WT-CFA1d							<0.0001	****
		WT-Basal vs.<br>WT-CFA2d							<0.0001	****

		WT-Basal vs. WT-CFA4d						<0.0001	****
		WT-Basal vs. WT-CFA7d						<0.0001	****
		6KO-Basal vs. 6KO-CFA1d						<0.0001	****
		6KO-Basal vs. 6KO-CFA2d						<0.0001	****
		6KO-Basal vs. 6KO-CFA4d						<0.0001	****
		6KO-Basal vs. 6KO-CFA7d						0.00039	***
		WT-Basal (n=5), DKO-Basal (n=5). WT-CFA1d (n=5), DKO-CFA1d (n=5). WT-CFA2d (n=5), DKO-CFA2d (n=5). WT-CFA4d (n=5), DKO-CFA4d (n=5). WT-CFA7d (n=5), DKO-CFA7d (n=5).	mouse	10 mice	W=0.860 P=0.227, W=0.970 P=0.875, W=0.870 P=0.268, W=0.960 P=0.809, W=0.966 P=0.846, W=0.903 P=0.428, W=0.921 P=0.534, W=0.867 P=0.254, W=0.822 P=0.122, W=0.802 P=0.084	F<0.0001,DFn=1,DFd=8, P=0.9997, F=4.159,DFn=1,DFd=8, P=0.076, F=1.777,DFn=1,DFd=8, P=0.219, F=3.264,DFn=1,DFd=8, P=0.108, F=2.504,DFn=1,DFd=8, P=0.152	Two-way repeated-measures ANOVA with post hoc comparison	F=164.682 ,DF=1	<0.0001	****
		WT-Basal vs. DKO-Basal						0.036	*
	Mechanical	WT-CFA1d vs. DKO-CFA1d						<0.0001	****

**Fig1J**	response threshold	WT-CFA2d vs. DKO-CFA2d							<0.0001	****
		WT-CFA4d vs. DKO-CFA4d							<0.0001	****
		WT-CFA7d vs. DKO-CFA7d							<0.0001	****
		WT-Basal vs. WT-CFA1d							0.00013	***
		WT-Basal vs. WT-CFA2d							0.00075	***
		WT-Basal vs. WT-CFA4d							0.00026	***
		WT-Basal vs. WT-CFA7d							0.00416	**
		DKO-Basal vs. DKO-CFA1d							0.053	ns
		DKO-Basal vs. DKO-CFA2d							0.622	ns
		DKO-Basal vs. DKO-CFA4d							0.724	ns
		DKO-Basal vs. DKO-CFA7d							0.299	ns
		WT-Basal (n=7), DKO-Basal (n=7). WT-CFA1d (n=7), DKO-CFA1d (n=7). WT-CFA7d (n=7), DKO-CFA7d (n=7).	mouse	14 mice	W=0.847 P=0.117, W=0.939 P=0.632, W=0.814 P=0.056, W=0.897 P=0.312, W=0.820 P=0.065, W=0.951 P=0.741	Not all subgroups meets p > 0.05	Friedman's M test	χ^2 =13.762, DF=1	0.00021	****

	Von Frey 0.40g ΔF/F	WT-Basal vs. DKO-Basal					Mann-Whitney U test		0.277	ns
		WT-CFA1d vs. DKO-CFA1d					Mann-Whitney U test		0.002	**
		WT-CFA7d vs. DKO-CFA7d					Mann-Whitney U test		0.002	**
		WT-Basal vs. WT-CFA1d					Mann-Whitney U test		0.002	**
		WT-Basal vs. WT-CFA7d					Mann-Whitney U test		0.002	**
		DKO-Basal vs. DKO-CFA1d					Mann-Whitney U test		0.406	ns
		DKO-Basal vs. DKO-CFA7d					Mann-Whitney U test		0.142	ns
Fig2C	Von Frey 4.0g ΔF/F	WT-Basal (n=7), DKO-Basal (n=7). WT-CFA1d (n=7), DKO-CFA1d (n=7). WT-CFA7d (n=7), DKO-CFA7d (n=7).	mouse	14 mice	W=0.919 P=0.460, W=0.958 P=0.806, W=0.910 P=0.397, W=0.851 P=0.127, W=0.948 P=0.714, W=0.879 P=0.223	Not all subgroups meets p > 0.05	Friedman's M test	χ^2 =21.000, DF=1	<0.0001	****
		WT-Basal vs. DKO-Basal					Mann-Whitney U test		0.003	**
		WT-CFA1d vs. DKO-CFA1d					Mann-Whitney U test		0.002	**
		WT-CFA7d vs. DKO-CFA7d					Mann-Whitney U test		0.002	**
		WT-Basal vs. WT-CFA1d					Mann-Whitney U test		0.002	**

		WT-Basal vs. WT-CFA7d					Mann-Whitney U test		0.002	**
		DKO-Basal vs. DKO-CFA1d					Mann-Whitney U test		0.064	ns
		DKO-Basal vs. DKO-CFA7d					Mann-Whitney U test		0.338	ns
	Von Frey 8.0g ΔF/F	WT-Basal (n=7), DKO-Basal (n=7). WT-CFA1d (n=7), DKO-CFA1d (n=7). WT-CFA7d (n=7), DKO-CFA7d (n=7).	mouse	14 mice	W=0.895 P=0.302, W=0.936 P=0.599, W=0.869 P=0.183, W=0.864 P=0.165, W=0.924 P=0.504, W=0.966 P=0.867	Not all subgroups meets p > 0.05	Friedman's M test	χ^2 =17.190, DF=1	<0.0001	****
		WT-Basal vs. DKO-Basal					Mann-Whitney U test		0.025	*
		WT-CFA1d vs. DKO-CFA1d					Mann-Whitney U test		0.002	**
		WT-CFA7d vs. DKO-CFA7d					Mann-Whitney U test		0.002	**
		WT-Basal vs. WT-CFA1d					Mann-Whitney U test		0.002	**
		WT-Basal vs. WT-CFA7d					Mann-Whitney U test		0.002	**
		DKO-Basal vs. DKO-CFA1d					Mann-Whitney U test		0.018	*
		DKO-Basal vs. DKO-CFA7d					Mann-Whitney U test		0.749	ns

	Brush ΔF/F	WT-Basal (n=7), DKO-Basal (n=7). WT-CFA1d (n=7), DKO-CFA1d (n=7). WT-CFA7d (n=7), DKO-CFA7d (n=7).	mouse	14 mice	Not all subgroups meets p > 0.05		Friedman's M test	χ2 =21.000, DF=1	<0.0001	****
		WT-Basal vs. DKO-Basal					Mann-Whitney U test		0.277	ns
		WT-CFA1d vs. DKO-CFA1d					Mann-Whitney U test		0.002	**
		WT-CFA7d vs. DKO-CFA7d					Mann-Whitney U test		0.002	**
		WT-Basal vs. WT-CFA1d					Mann-Whitney U test		0.002	**
		WT-Basal vs. WT-CFA7d					Mann-Whitney U test		0.002	**
		DKO-Basal vs. DKO-CFA1d					Mann-Whitney U test		0.749	ns
		DKO-Basal vs. DKO-CFA7d					Mann-Whitney U test		0.225	ns
		WT-Basal (n=7), DKO-Basal (n=7). WT-CFA1d (n=7), DKO-CFA1d (n=7). WT-CFA7d (n=7), DKO-CFA7d (n=7).	mouse	14 mice	W=0.833 P=0.085, W=0.962 P=0.836, W=0.841 P=0.101, W=0.898 P=0.322, W=0.896 P=0.307, W=0.920 P=0.469	Not all subgroups meets p > 0.05	Friedman's M test	χ2 =10.714, DF=1	0.0011	**

**Fig2E**	Pressure ΔF/F	WT-Basal vs. DKO-Basal					Mann-Whitney U test		0.565	ns
		WT-CFA1d vs. DKO-CFA1d					Mann-Whitney U test		0.002	**
		WT-CFA7d vs. DKO-CFA7d					Mann-Whitney U test		0.002	**
		WT-Basal vs. WT-CFA1d					Mann-Whitney U test		0.002	**
		WT-Basal vs. WT-CFA7d					Mann-Whitney U test		0.002	**
		DKO-Basal vs. DKO-CFA1d					Mann-Whitney U test		0.749	ns
		DKO-Basal vs. DKO-CFA7d					Mann-Whitney U test		0.004	**
	Pinch ΔF/F	WT-Basal (n=7), DKO-Basal (n=7). WT-CFA1d (n=7), DKO-CFA1d (n=7). WT-CFA7d (n=7), DKO-CFA7d (n=7).	mouse	14 mice	W=0.845 P=0.110, W=0.969 P=0.888, W=0.934 P=0.582, W=0.959 P=0.810, W=0.906 P=0.366, W=0.892 P=0.286	Not all subgroups meets p > 0.05	Friedman's M test	$\chi 2$ =21.000, DF=1	<0.0001	****
		WT-Basal vs. DKO-Basal					Mann-Whitney U test		0.006	**
		WT-CFA1d vs. DKO-CFA1d					Mann-Whitney U test		0.002	**
		WT-CFA7d vs. DKO-CFA7d					Mann-Whitney U test		0.002	**
		WT-Basal vs. WT-CFA1d					Mann-Whitney U test		0.002	**

		WT-Basal vs. WT-CFA7d					Mann-Whitney U test		0.002	**
		DKO-Basal vs. DKO-CFA1d					Mann-Whitney U test		0.013	*
		DKO-Basal vs. DKO-CFA7d					Mann-Whitney U test		0.004	**
**Fig3A**	Relative TRPC3 levels	WT-Basal(n=4), WT-CFA(n=4)	mouse	8 mice	W=0.852 P=0.234, W=0.982 P=0.914	F=15.23, DFn=3, DFd=3, P=0.0509	Unpaired t test with Welch's correction	t=5.188 DF=3.392	0.0102	*
	Relative TRPC6 levels	WT-Basal(n=4), WT-CFA(n=4)	mouse	8 mice	W=0.908 P=0.472, W=0.896 P=0.414	F=82.86, DFn=3, DFd=3, P=0.0044	Unpaired t test with Welch's correction	t=5.316 DF=3.072	0.0122	*
**Fig3B**	Relative TRPC3 levels	WT-Basal(n=4), WT-CFA(n=4)	mouse	8 mice	W=0.925 P=0.566, W=0.879 P=0.336	F=14.87, DFn=3, DFd=3, P=0.0527	Unpaired t test with Welch's correction	t=11.000 DF=3.402	0.0009	***
	Relative TRPC6 levels	WT-Basal(n=4), WT-CFA(n=4)	mouse	8 mice	W=0.976 P=0.879, W=0.884 P=0.357	F=17.93, DFn=3, DFd=3, P=0.0406	Unpaired t test with Welch's correction	t=7.668 DF=3.334	0.0031	**
**Fig3C**	TRPC3/ β-actin	Basal (n=3), CFA(n=3)	mouse	6 mice	W=0.803 P=0.122, W=0.819 P=0.161	F=3.242, DFn=1, DFd=4, P=0.146	Two-tailed unpaired t-test	t=5.391, DF=4	0.006	**
	TRPC6/ β-actin	Basal (n=3), CFA(n=3)	mouse	6 mice	W=0.784 P=0.078, W=0.889 P=0.350	F=3.125, DFn=1, DFd=4, P=0.152	Two-tailed unpaired t-test	t=3.454, DF=4	0.026	*
**Fig3D**	TRPC3/ β-actin	Basal (n=3), CFA(n=3)	mouse	6 mice	W=0.964 P=0.635, W=0.842 P=0.220	F=0.949, DFn=1, DFd=4, P=0.385	Two-tailed unpaired t-test	t=3.150, DF=4	0.035	*
	TRPC6/ β-actin	Basal (n=3), CFA(n=3)	mouse	6 mice	W=0.904 P=0.399 W=0.812 P=0.144	F=0.349, DFn=1, DFd=4, P=0.586	Two-tailed unpaired t-test	t=3.126, DF=4	0.035	*

**Fig3G**	TRPC3 in IB4	Basal (n=3), CFA(n=5)	mouse	8 mice	W=0.973 P=0.687, W=0.886 P=0.336	F=12.307, DFn=1, DFd=6, P=0.013	Two-tailed unpaired separate variance estimation t-test	t=4.893 DF=4.059	0.008	**
	TRPC3 in CGRP	Basal (n=4), CFA(n=5)	mouse	9 mice	W=0.899 P=0.428 W=0.858 P=0.221	F=8.733, DFn=1, DFd=7, P=0.021	Two-tailed unpaired separate variance estimation t-test	t=3.330, DF=5.710	0.017	*
	TRPC3 in NF200	Basal (n=5), CFA(n=3)	mouse	8 mice	W=0.938 P=0.649 W=0.948 P=0.560	F=1.143, DFn=1, DFd=6 P=0.326	Two-tailed unpaired t-test	t=0.181, DF=6	0.862	ns
**Fig3H**	TRPC6 in IB4	Basal (n=5), CFA(n=5)	mouse	10 mice	Not all subgroups meets p > 0.05		Mann-Whitney U test	U=1.000	0.016	*
	TRPC6 in CGRP	Basal (n=7), CFA(n=7)	mouse	14 mice	W=0.902 P=0.340, W=0.878 P=0.216	F=0.955 DFn=1, DFd=12, P=0.348	Two-tailed unpaired t-test	t=8.655, DF=12	<0.0001	****
	TRPC6 in NF200	Basal (n=4), CFA(n=5)	mouse	9 mice	W=0.973 P=0.862 W=0.822 P=0.121	F=2.018, DFn=1, DFd=7 P=0.198	Two-tailed unpaired t-test	t=4.256, DF=7	0.004	**
**Fig3J**	TRPC3 in IB4	Basal (n=4), CFA(n=5)	mouse	9 mice	W=0.788 P=0.082, W=0.892 P=0.367	F=0.233 DFn=1, DFd=7, P=0.651	Two-tailed unpaired t-test	t=4.223, DF=7	0.004	**
	TRPC3 in CGRP	Basal (n=5), CFA(n=5)	mouse	10 mice	W=0.960 P=0.807 W=0.841 P=0.168	F=3.279, DFn=1, DFd=8 P=0.108	Two-tailed unpaired t-test	t=5.134, DF=8	0.0009	***
**Fig3L**	TRPC6 in IB4	Basal (n=4), CFA(n=4)	mouse	8 mice	W=0.831 P=0.170, W=0.866 P=0.284	F=5.305 DFn=1, DFd=6, P=0.061	Two-tailed unpaired t-test	t=4.698, DF=6	0.003	**
	TRPC6 in CGRP	Basal (n=4), CFA(n=5)	mouse	9 mice	W=0.803 P=0.108 W=0.932 P=0.608	F=7.227, DFn=1, DFd=7, P=0.031	Two-tailed unpaired separate variance estimation t-test	t=8.115, DF=4.661	0.00064	***

**Fig3M**	TRPC3/ GAPDH	Ctrl (n=3), BPA (n=3)	human	6 humans	W=0.9963 P=0.884, W=0.9955 P=0.872	F=2.189, DFn=1, DFd=4, P=0.213	Two-tailed unpaired t-test	t=6.186, DF=4	0.003	**
**Fig3N**	TRPC6/ GAPDH	Ctrl (n=3), BPA (n=3)	human	6 humans	W=0.998 P=0.909, W=0.995 P=0.868	F=1.239, DFn=1, DFd=4, P=0.328	Two-tailed unpaired t-test	t=2.923, DF=4	0.043	*
**Fig4C**	RMP	WT-Basal (n=7), DKO-Basal (n=10). WT-CFA (n=12), DKO-CFA(n=6)	Neuron	5 mice per group	W=0.987 P=0.987, W=0.918 P=0.338 W=0.930 P=0.380, W=0.948 P=0.726	F=1.333, DFn=3, DFd=31,P=0.282	One-way ANOVA	F=1.280, DFn=3, DFd=31	0.298	ns
	Rm	WT-Basal (n=6), DKO-Basal (n=9). WT-CFA (n=12), DKO-CFA(n=6)	Neuron	5 mice per group	Not all subgroups meets p > 0.05		Kruskal-Wallis H test	χ2=9.898, DF=3	0.019	*
		WT-Basal vs. DKO-Basal					Nemenyi multiple comparisons test		0.769	ns
		WT-CFA vs. DKO-CFA					Nemenyi multiple comparisons test		0.705	ns
	Cm	WT-Basal (n=7), DKO-Basal (n=9). WT-CFA (n=12), DKO-CFA(n=6)	Neuron	5 mice per group	Not all subgroups meets p > 0.05		Kruskal-Wallis H test	χ2=2.843, DF=3	0.416	ns

**Fig4E**	AP frequency	WT-Basal (n=7), DKO-Basal (n=10). WT-CFA (n=12), DKO-CFA(n=6)	Neuron	5 mice per group	W=0.927 P=0.525, W=0.8712 P=0.103 W=0.8714 P=0.068, W=0.908 P=0.423	F=2.654, DFn=3, DFd=31,P=0.066	One-way ANOVA	F=42.266, DFn=3, DFd=31	<0.0001	****
		WT-Basal vs. DKO-Basal					Bonferroni's multiple comparisons test		0.04	*
		WT-CFA vs. DKO-CFA					Bonferroni's multiple comparisons test		<0.0001	****
	AP rheobase	WT-Basal (n=7), DKO-Basal (n=10). WT-CFA (n=12), DKO-CFA(n=6)	Neuron	5 mice per group	Not all subgroups meets p > 0.05		Kruskal-Wallis H test	$\chi2$ =24.258, DF=3	<0.0001	****
		WT-Basal vs. DKO-Basal					Nemenyi multiple comparisons test		0.036	*
		WT-CFA vs. DKO-CFA					Nemenyi multiple comparisons test		0.021	*
**Fig4F**	AP threshold	WT-Basal (n=7), DKO-Basal (n=10). WT-CFA (n=12), DKO-CFA(n=6)	Neuron	5 mice per group	Not all subgroups meets p > 0.05		Kruskal-Wallis H test	$\chi2$=4.823, DF=3	0.185	ns
	AP amplitude	WT-Basal (n=7), DKO-Basal (n=10). WT-CFA (n=12), DKO-CFA(n=6)	Neuron	5 mice per group	Not all subgroups meets p > 0.05		Kruskal-Wallis H test	$\chi2$=3.514, DF=3	0.319	ns

	AP half-width	WT-Basal (n=7), DKO-Basal (n=10). WT-CFA (n=12), DKO-CFA(n=6)	Neuron	5 mice per group	Not all subgroups meets p > 0.05		Kruskal-Wallis H test	χ2=5.923, DF=3	0.115	ns
**Fig5 B&C**	Amplitude of C-eEPSCs	WT-0.1mA (n=7), DKO-0.1mA (n=6),WT-0.3mA (n=7), DKO-0.3mA (n=6),WT-0.5mA (n=7), DKO-0.5mA (n=6),WT-0.8mA (n=7), DKO-0.8mA (n=6),WT-1mA (n=7), DKO-1mA (n=6), WT-2mA (n=7), DKO-2mA (n=6),WT-3mA (n=7), DKO-3mA (n=6).	Neuron	5 mice per group	Not all subgroups meets p > 0.05		Friedman's M test	χ2 =38.095, DF=1	<0.0001	****
		WT-0.1mA vs. DKO-0.1mA					Mann-Whitney U test		<0.0001	****
		WT-0.3mA vs. DKO-0.3mA					Mann-Whitney U test		<0.0001	****
		WT-0.5mA vs. DKO-0.5mA					Mann-Whitney U test		<0.0001	****
		WT-0.8mA vs. DKO-0.8mA					Mann-Whitney U test		<0.0001	****
		WT-1mA vs. DKO-1mA					Mann-Whitney U test		<0.0001	****
		WT-2mA vs. DKO-2mA					Mann-Whitney U test		<0.0001	****

		WT-3mA vs. DKO-3mA					Mann-Whitney U test		<0.0001	****
**Fig5F**	Relative amplitude of C-eEPSCs	WT-befroe LFS (n=10), DKO-befroe LFS (n=10). WT-after LFS (n=9), DKO-after LFS (n=9)	Neuron	5 mice per group	W=0.983 P=0.975, W=0.950 P=0.728	F=7.559, DFn=1, DFd=13,P=0.017	Kruskal-Wallis H test	χ2 =27.968, DF=3	<0.0001	****
		WT-befroe LFS vs. WT-after LFS					Nemenyi multiple comparisons test		0.005	**
		WT-after LFS vs. DKO-after LFS					Nemenyi multiple comparisons test		<0.001	***
**Fig5I**	Vertical distance to the diagonal	WT (n=11), TRPC3/6 DKO (n=7)	Neuron	5 mice per group	W=0.886,P=0.124 W=0.916,P=0.437	F=1.387, DFn=1, DFd=16, P=0.256	Two-tailed unpaired t-test	t=2.378 DF=16	0.03	*
**Fig5M**	Relative fluorescene intensity	WT-befroe LFS (n=5), DKO-befroe LFS (n=5). WT-after LFS (n=5), DKO-after LFS (n=5)	Neuron	5 mice per group	W=0.860,P=0.228 W=0.960,P=0.811 W=0.989,P=0.966 W=0.884,P=0.328	F=2.194, DFn=1, DFd=16,P=0.128	One-way ANOVA	F=296.546 , DFn=3, DFd=16	<0.0001	****
		WT-befroe LFS vs. DKO before LFS					Tukey's multiple comparisons test		0.9987	ns
		WT-befroe LFS vs. WT-after LFS					Tukey's multiple comparisons test		<0.0001	****
		DKO-before LFS vs. DKO-after LFS					Tukey's multiple comparisons test		<0.0001	****
		WT-after LFS vs. DKO-after LFS					Tukey's multiple comparisons test		<0.0001	****

**Fig6 B&C**	Amplitude of C-eEPSCs	WT 0.1mA (n=11),DKO 0.1mA (n=13),WT 0.3mA (n=11),DKO 0.3mA (n=13),WT 0.5mA (n=11),DKO 0.5mA (n=13),WT 1mA (n=11),DKO 1mA (n=13), WT 2mA (n=11),DKO 2mA (n=13),WT 3mA (n=11),DKO 3mA (n=13).	Neuron	5 mice per group	Not all subgroups meets p > 0.05		Friedman's M test	$\chi 2$ =19.558, DF=1	<0.0001	****
		WT 0.1mA vs. DKO 0.1mA					Mann-Whitney U test		1	ns
		WT 0.3mA vs. DKO 0.3mA					Mann-Whitney U test		0.856	ns
		WT 0.5mA vs. DKO 0.5mA					Mann-Whitney U test		0.018	*
		WT 1mA vs. DKO 1mA					Mann-Whitney U test		0.011	*
		WT 2mA vs. DKO 2mA					Mann-Whitney U test		0.156	ns
		WT 3mA vs. DKO 3mA					Mann-Whitney U test		0.008	**

**Fig6 E&F**	Amplitude of C-eEPSCs	WT CFA-0.1mA (n=11), DKO CFA-0.1mA (n=13),WT CFA-0.3mA (n=11), DKO CFA-0.3mA (n=13),WT CFA-0.5mA (n=11), DKO CFA-0.5mA (n=13),WT CFA-1mA (n=11), DKO CFA-1mA (n=13), WT CFA-2mA (n=11), DKO CFA-2mA (n=13),WT CFA-3mA (n=11), DKO CFA-3mA (n=13).	Neuron	5 mice per group	W=0.869 P=0.096, W=0.686 P=0.003, W=0.917 P=0.336, W=0.941 P=0.650, W=0.878 P=0.122, W=0.867 P=0.176, W=0.934 P=0.490, W=0.821 P=0.066, W=0.969 P=0.879, W=0.953 P=0.753	F=0.266, DFn=1, DFd=15, P=0.614, F=2.039, DFn=1, DFd=15, P=0.174, F=4.510, DFn=1, DFd=15, P=0.051, F=3.956, DFn=1, DFd=15, P=0.065, F=5.216, DFn=1, DFd=15, P=0.037	Two-way repeated-measures ANOVA with post hoc comparison	F=9.865, DF=1	0.007	**
		WT CFA-0.1mA vs. DKO CFA-0.1mA							1	ns
		WT CFA-0.3mA vs. DKO CFA-0.3mA							0.464	ns
		WT CFA-0.5mA vs. DKO CFA-0.5mA							0.146	ns
		WT CFA-1mA vs. DKO CFA-1mA							0.035	*
		WT CFA-2mA vs. DKO CFA-2mA							0.0103	*
		WT CFA-3mA vs. DKO CFA-3mA							0.004	**
		WT (n=10), CFA (n=10)	Neuron	5 mice per group	Not all subgroups meets p > 0.05		Mann-Whitney U test	21	0.0272	*

**Fig6G**	PPR	TRPC3/6 DKO (n=14), TRPC3/6 DKO + CFA (n=10),	Neuron	5 mice per group	Not all subgroups meets p > 0.05		Mann-Whitney U test	57	0.4708	ns
**Fig6I**	Frequency	WT (n=5), TRPC3/6 DKO (n=5)	Neuron	5 mice per group	W=0.963 P=0.633, W=0.955 P=0.594	F=0.430, DFn=1, DFd=8, P=0.266	Two-tailed unpaired t-test	t=4.083 DF=8	0.004	**
**Fig6J**	Amplitude	WT (n=5), TRPC3/6 DKO (n=5)	Neuron	5 mice per group	W=0.963 P=0.633, W=0.955 P=0.594	F=0.754, DFn=1, DFd=8, P=0.411	Two-tailed unpaired t-test	t=0.026 DF=8	0.98	NS
**Fig7B**	BDNF/ β-actin	WT Basal (n=3), TRPC3/6 DKO Basal (n=3), WT CFA (n=3), TRPC3/6 DKO CFA (n=3)	mouse	12 mice	W=0.841 P=0.216, W=0.929 P=0.486, W=0.958 P=0.607, W=0.898 P=0.380	F=2.486, DFn=3, DFd=8, P=0.135	One-way ANOVA	F=8.495, DFn=3, DFd=8	0.007	**
		WT Basal vs. WT CFA					Tukey's multiple comparisons test		0.005	**
		WT CFA vs. DKO CFA					Tukey's multiple comparisons test		0.048	*
**Fig7G**	TRPC3/ β-actin	conRNA (n=3), shTRPC3/6 (n=3)	mouse	6 mice	W=0.963 P=0.633, W=0.955 P=0.594	F=0.588, DFn=1, DFd=4, P=0.486	Two-tailed unpaired t-test	t=5.729, DF=4	0.005	**
	TRPC6/ β-actin	conRNA (n=3), shTRPC3/6 (n=3)	mouse	6 mice	W=0.951 P=0.575, W=0.926 P=0.473	F=1.659, DFn=1, DFd=4, P=0.267	Two-tailed unpaired t-test	t=8.346, DF=4	0.0011	**
**Fig7J**	Relative fluorescence intensity	conRNA (n=20), shTRPC3/6 (n=20)	mouse	6 mice	W=0.930 P=0.158, W=0.971 P=0.769	F=1.237, DFn=1, DFd=38, P=0.273	Two-tailed unpaired t-test	t=9.982, DF=38	<0.0001	****

Fig8C	Mechanical response threshold	conRNA Basal (n=5),shTRPC3/6 Basal (n=5),conRNA 6h (n=5),shTRPC3/6 6h (n=5),conRNA 12h (n=5),shTRPC3/6 12h (n=5),conRNA 24h(n 5),shTRPC3/6 24h (n=5),conRNA 48h (n 5),shTRPC3/6 48h (n=5),conRNA 96h (n=5),shTRPC3/6 96h (n=5).	mouse	10 mice	W=0.895 P=0.383, W=0.933 P=0.616, W=0.900 P=0.409, W=0.915 P=0.501, W=0.843 P=0.175, W=0.882 P=0.320, W=0.860 P=0.228, W=0.847 P=0.184, W=0.785 P=0.060, W=0.988 P=0.971, W=0.787 P=0.063, W=0.882 P=0.317	F=1.477, DFn=1, DFd=8, P=0.259, F=4.310, DFn=1, DFd=8, P=0.072, F=2.994, DFn=1, DFd=8, P=0.122, F=3.361, DFn=1, DFd=8, P=0.104, F=4.084, DFn=1, DFd=8, P=0.078, F=2.636, DFn=1, DFd=8, P=0.143	Two-way repeated-measures ANOVA with post hoc comparison	F=124.637 ,DF=1	<0.0001	****
		conRNA Basal vs. shTRPC3/6 Basal							0.051	ns
		conRNA 6h vs. shTRPC3/6 6h							<0.0001	****
		conRNA 12h vs. shTRPC3/6 12h							<0.0001	****
		conRNA 24h vs. shTRPC3/6 24h							<0.0001	****
		conRNA 48h vs. shTRPC3/6 48h							<0.0001	****
		conRNA 96h vs. shTRPC3/6 96h							<0.0001	****

		conRNA Basal vs.conRNA 6h							<0.0001	****
		conRNA Basal vs.conRNA 12h							<0.0001	****
		conRNA Basal vs.conRNA 24h							<0.0001	****
		conRNA Basal vs.conRNA 48h							<0.0001	****
		conRNA Basal vs.conRNA 96h							<0.0001	****
		shTRPC3/6 Basal vs. shTRPC3/6 6h							<0.0001	****
		shTRPC3/6 Basal vs. shTRPC3/6 12h							<0.0001	****
		shTRPC3/6 Basal vs. shTRPC3/6 24h							0.000103	***
		shTRPC3/6 Basal vs. shTRPC3/6 48h							<0.0001	****
		shTRPC3/6 Basal vs. shTRPC3/6 96h							0.0008	***

Fig8F	Mechanical response threshold	conRNA Basal (n=5),shBDNF Basal (n=5),conRNA 6h (n=5),shBDNF 6h (n=5),conRNA 12h (n=5),shBDNF 12h (n=5),conRNA 24h(n=5),shBDNF 24h (n=5),conRNA 48h (n=5),shBDNF 48h (n=5),conRNA 96h (n=5),shBDNF 96h (n=5).	mouse	10 mice	W=0.993 P=0.989, W=0.942 P=0.680, W=0.925 P=0.564, W=0.952 P=0.752, W=0.877 P=0.295, W=0.943 P=0.689, W=0.7804 P=0.0556, W=0.923 P=0.548, W=0.813 P=0.103, W=0.862 P=0.236, W=0.7807 P=0.0559, W=0.817 P=0.110	F=0.306, DFn=1, DFd=8, P=0.596, F=0.145, DFn=1, DFd=8, P=0.713, F=0.010, DFn=1, DFd=8, P=0.925, F=0.560, DFn=1, DFd=8, P=0.476, F=1.479, DFn=1, DFd=8, P=0.259, F=0.316, DFn=1, DFd=8, P=0.589	Two-way repeated-measures ANOVA with post hoc comparison	F=63.560, DF=1	<0.0001	****
		conRNA Basal vs. shBDNF Basal							0.49	ns
		conRNA 6h vs. shBDNF 6h							<0.0001	****
		conRNA 12h vs. shBDNF 12h							<0.0001	****
		conRNA 24h vs. shBDNF 24h							<0.0001	****
		conRNA 48h vs. shBDNF 48h							0.0017	**
		conRNA 96h vs. shBDNF 96h							<0.0001	****
		conRNA Basal vs.conRNA 6h							<0.0001	****
		conRNA Basal vs.conRNA 12h							<0.0001	****

		conRNA Basal vs.conRNA 24h							<0.0001	****
		conRNA Basal vs.conRNA 48h							<0.0001	****
		conRNA Basal vs.conRNA 96h							<0.0001	****
		shBDNF Basal vs. shBDNF 6h							<0.0001	****
		shBDNF Basal vs. shBDNF 12h							<0.0001	****
		shBDNF Basal vs. shBDNF 24h							<0.0001	****
		shBDNF Basal vs. shBDNF 48h							<0.0001	****
		shBDNF Basal vs. shBDNF 96h							0.0058	**
**Fig8G**	BK in Plantar skin	C57-Basal(n=3), C57-CFA 3h(n=3), C57-CFA 6h(n=3), C57-CFA 12h(n=3), C57-CFA 24h(n=3)	mouse	15mice	W=0.7970, P=0.1073, W=0.9774, P=0.712, W=0.9582, P=0.6066, W=0.8343, P=0.1994, W=0.8812, P=0.3278	F=1.133, DFn=2, DFd=2, P=0.9378, F=2.502, DFn=2, DFd=2, P=0.5711, F=2.744, DFn=2, DFd=2, P=0.5342, F=12.15, DFn=2, DFd=2, P=0.1521	Brown-Forsythe and Welch ANOVA tests	F = 9.806, DFn = 4, DFd = 3.764	0.0278	*
		C57-Basal vs C57-CFA 3h						*	0.0107	*
		C57-Basal vs C57-CFA 6h						ns	0.6963	ns
		C57-Basal vs C57-CFA 12h						ns	0.7173	ns
		C57-Basal vs C57-CFA 24h						ns	>0.9999	ns

**Fig8H**	BK in DRG	C57-Basal(n=3), C57-CFA 3h(n=3), C57-CFA 6h(n=3), C57-CFA 12h(n=3), C57-CFA 24h(n=3)	mouse	15mice	W=0.785, P=0.079, W=0.807, P=0.132, W=0.985, P=0.766, W=0.944, P=0.5423, W=1, P=0.964	F=1.363, DFn=2, DFd=2, P=0.8462, F=1.124, DFn=2, DFd=2, P=0.9418, F=3.198, DFn=2, DFd=2, P=0.4765, F=1.184, DFn=2, DFd=2, P=0.9156	Brown-Forsythe and Welch ANOVA tests	F = 4.227, DFn = 4, DFd = 10	0.0294	*
		C57-Basal vs C57-CFA 3h							0.0248	*
		C57-Basal vs C57-CFA 6h							0.1802	ns
		C57-Basal vs C57-CFA 12h							0.9699	ns
		C57-Basal vs C57-CFA 24h							0.397	ns
	TRPC3 / Flotillin	C57-Basal(n=4), C57-BK(n=4), C57-BK+Icatibant (n=4), C57-BK+SSR240612(n=4)	mouse	16mice	W=0.8673, P=0.2874, W=0.9781, P=0.8909, W=0.8311, P=0.1706, W=0.9636, P=0.8014	F=1.407, DFn=3, DFd=3, P=0.7859, F=3.307, DFn=3, DFd=3, P=0.3521, F=1.302, DFn=3, DFd=3, P=0.8336	Brown-Forsythe and Welch ANOVA tests	F = 8.653, DFn = 3, DFd = 10.13	0.0038	**
		Basal vs. BK							0.0252	*
		BK vs. BK+Icatibant							0.0269	*
		BK vs. BK+SSR240612							0.0181	*

**Fig8J**	TRPC6 / Flotillin	C57-Basal(n=4), C57-BK(n=4), C57-BK+Icatibant (n=4), C57-BK+SSR240612(n=4)	mouse	16mice	W=0.9358, P=0.6292, W=0.7555, P=0.0434, W=0.7343, P=0.0272, W=0.8089, P=0.1191	F=1.26, DFn=3, DFd=3, P=0.8541, F=6.843, DFn=3, DFd=3, P=0.1485 F=5.889, DFn=3, DFd=3, P=0.1794	Brown-Forsythe and Welch ANOVA tests	F = 5.208, DFn = 3, DFd = 12	0.0156	*
		Basal vs. BK							0.0317	*
		BK vs. BK+Icatibant							0.0326	*
		BK vs. BK+SSR240612							0.0121	*
**Fig8L**	Response duration	conRNA Basal (n=5), shTRPC3/6 Basal (n=5)	mouse	10 mice	W=0.979,P=0.927 W=0.988,P=0.972	F=0.665, DFn=1, DFd=8, P=0.438	Two-tailed unpaired t-test	t=5.713 DF=8	0.0004	***
**Fig8N**	Amplitude of eEPSCs	WT-Ctrl (n=5), WT+BDNF (n=5). DKO-Ctrl (n=7), DKO+BDNF (n=7).	mouse	12 mice	Not all subgroups meets p > 0.05		Friedman's M test	χ2=4.920, DF=3	0.178	ns
		WT-Ctrl vs.WT+BDNF					Wilcoxon's Sign Rank Test	Z=2.023	0.043	*

		Vehicle Basal (n=6),SAR7334 0.1μM Basal (n=6),SAR7334 1.0μM Basal (n=6),SAR7334 Basal 10.0μM(n=6). Vehicle 0.5h+CFA (n=6),SAR7334 0.1μM 0.5h+CFA(n=6),SAR7334 1.0μM 0.5h+CFA (n=6),SAR7334 10.0μM 0.5h+CFA (n=6).Vehicle 1.5h+CFA (n=6),SAR7334 0.1μM 1.5h+CFA(n=6),SAR7334 1.0μM 1.5h+CFA (n=6),SAR7334 10.0μM 1.5h+CFA (n=6).Vehicle 3.0h+CFA (n=6),SAR7334 0.1μM 3.0h+CFA(n=6),SAR7334 1.0μM 3.0h+CFA (n=6),SAR7334 10.0μM 3.0h+CFA (n=6).Vehicle 5.0h+CFA (n=6),SAR7334 0.1μM 5.0h+CFA(n=6),SAR7334 1.0μM 5.0h+CFA (n=6),SAR7334 10.0μM 5.0h+CFA (n=6)	mouse	24 mice	W=0.881 P=0.272, W=0.898 P=0.361, W=0.932 P=0.595, W=0.881 P=0.272, W=0.815 P=0.080, W=0.915 P=0.472, W=0.894 P=0.340,W=0.918 P=0.491,W=0.801 P=0.060, W=0.865 P=0.206, W=0.825 P=0.097, W=0.842 P=0.136,W=0.807 P=0.068, W=0.834 P=0.117, W=0.898 P=0.364, W=0.888 P=0.307,W=0.830 P=0.106, W=0.841 P=0.133, W=0.883 P=0.284, W=0.802 P=0.062.	F=1.029, DFn=3, DFd=20, P=0.401, F=1.594, DFn=3, DFd=20, P=0.222, F=2.141, DFn=3, DFd=20, P=0.127, F=1.126, DFn=3, DFd=20, P=0.362, F=1.330, DFn=3, DFd=20, P=0.293.	Two-way repeated-measures ANOVA with post hoc comparison	F=47.003, DF=3	<0.0001	****
		Vehicle Basal vs.Vehicle 0.5h+CFA							<0.0001	****
		Vehicle Basal vs.Vehicle 1.5 h+CFA							<0.0001	****
		Vehicle Basal vs.Vehicle 3.0h+CFA							<0.0001	****

**Fig8O**	Mechanical response threshold	Vehicle Basal vs.Vehicle 5.0h+CFA							<0.0001	****
		Vehicle Basal vs. SAR7334 0.1μM Basal							0.99989	ns
		Vehicle Basal vs. SAR7334 1.0μM Basal							0.99999	ns
		Vehicle Basal vs. SAR7334 10.0μM Basal							0.98290	ns
		Vehicle 0.5h+CFAI vs. SAR7334 0.1μM 0.5h Basal							<0.0001	****
		Vehicle 0.5h+CFAI vs. SAR7334 1.0μM 0.5h Basal							<0.0001	****
		Vehicle 0.5h+CFAI vs. SAR7334 10.0μM 0.5h Basal							<0.0001	****
		Vehicle 1.5h+CFAI vs. SAR7334 0.1μM 1.5h Basal							<0.0001	****
		Vehicle 1.5h+CFAI vs. SAR7334 1.0μM 1.5h Basal							<0.0001	****
		Vehicle 1.5h+CFAI vs. SAR7334 10.0μM 1.5h Basal							<0.0001	****

		Vehicle 3.0h+CFAI vs. SAR7334 0.1μM 3.0h Basal							0.612	ns
		Vehicle 3.0h+CFAI vs. SAR7334 1.0μM 3.0h Basal							<0.0001	****
		Vehicle 3.0h+CFAI vs. SAR7334 10.0μM 3.0h Basal							<0.0001	****
		Vehicle 5.0h+CFAI vs. SAR7334 0.1μM 5.0h Basal							0.74109	ns
		Vehicle 5.0h+CFAI vs. SAR7334 1.0μM 5.0h Basal							0.89965	ns
		Vehicle 5.0h+CFAI vs. SAR7334 10.0μM 5.0h Basal							0.99962	ns
**Fig8P**	Number of spike	Basal (n=5), SAR7334(n=5)	neuron	5 humans	W=0.883 P=0.325		Paired sample test	t=6.325, DF=4	0.003	**
**Sfig1A**	Latency to fall	WT (n=6), TRPC3 KO (n=6)	mouse	12 mice	W=0.892 P=0.326, W=0.963 P=0.840	F=0.034, DFn=1, DFd=10, P=0.858	Two-tailed unpaired t-test	t=0.367, DF=10	0.721	ns
**Sfig1B**	Latency to fall	WT (n=6), TRPC6 KO (n=6)	mouse	12 mice	W=0.844 P=0.141, W=0.851 P=0.160	F=1.335, DFn=1, DFd=10, P=0.275	Two-tailed unpaired t-test	t=0.236, DF=10	0.818	ns
**Sfig1C**	Latency to fall	WT (n=6), TRPC3/6DKO (n=6)	mouse	12 mice	W=0.911 P=0.441, W=0.917 P=0.481	F=0.248, DFn=1, DFd=10, P=0.629	Two-tailed unpaired t-test	t=0.525, DF=10	0.611	ns

**Sfig2D**	CGRP expressing cells	WT (n=10), TRPC3/6DKO (n=9)	mouse	19 mice	W=0.962 P=0.813, W=0.929 P=0.474	F=6.754, DFn=1, DFd=17, P=0.019	Two-tailed unpaired separate variance estimation t-test	t=0.419, DF=12.407	0.682	ns
	IB4 expressing cells	WT (n=10), TRPC3/6DKO (n=10)	mouse	20 mice	W=0.880 P=0.130, W=0.951 P=0.683	F=0.004, DFn=1, DFd=18, P=0.950	Two-tailed unpaired t-test	t=1.151, DF=18	0.265	ns
	NF200 expressing cells	WT (n=8), TRPC3/6DKO (n=8)	mouse	16 mice	W=0.903 P=0.310, W=0.969 P=0.894	F=0.365, DFn=1, DFd=14, P=0.555	Two-tailed unpaired t-test	t=0.660, DF=14	0.52	ns
**Sfig2F**	Intensity of IB4	WT (n=5), TRPC3/6DKO (n=5)	mouse	10 mice	W=0.846 P=0.183, W=0.853 P=0.205	F=0.819, DFn=1, DFd=8, P=0.392	Two-tailed unpaired t-test	t=0.186, DF=8	0.857	ns
	Intensity of CGRP	WT (n=5), TRPC3/6DKO (n=5)	mouse	10 mice	W=0.934 P=0.624, W=0.892 P=0.365	F=4.590 DFn=1, DFd=8, P=0.065	Two-tailed unpaired t-test	t=1.279, DF=8	0.237	ns
**Sfig2I**	NeuN expressing cells (% total cells)	WT (n=10), TRPC3/6DKO (n=10)	mouse	20 mice	W=0.896 P=0.199, W=0.974 P=0.927	F=0.070, DFn=1, DFd=18, P=0.795	Two-tailed unpaired t-test	t=0.579, DF=18	0.57	ns
**SFig7D**	BDNF/ β-actin	conRNA (n=4), shBDNF (n=4)	mouse	10 mice	W=0.9436 P=0.6763, W=0.8376 P=0.1884	F=2.013, DFn=3, DFd=3, P=0.5802	Two-tailed unpaired t-test	t=4.763, DF=6	0.0031	**
**Sfig8C**	Number of spike	Basal (n=3), GSK283(n=3)	neuron	1 human	W=0.75 P= , W=1.000 P＞0.9999	F=3.0, DFn=2, Dfd=2	Two-tailed unpaired t-test	t=5.0, DF=4	0.0075	**
