# Supplementary material for: TRPC3/6 Channels Mediate Mechanical Pain Hypersensitivity via Enhancement of Nociceptor Excitability and of Spinal Synaptic Transmission
Source: Adv Sci (Weinh). 2024 Sep 28;11(44):2404342. doi: 10.1002/advs.202404342 (PMC11600220; doi:10.1002/advs.202404342)
Supplement: Supplementary file 2 — Supporting Tables [file ADVS-11-2404342-s002.zip › Supplementary Table 1-1.docx]

**KEY RESOURCES TABLE**

| **REAGENT or RESOURCE** | **SOURCE** | **IDENTIFIER** | **Dilution** |
| --- | --- | --- | --- |
| Antibodies | | | |
| Rabbit anti-TRPC3 | Cell Signaling Technology, Boston, MA, USA | 77934S | 1:1000 (WB)  1:200（IF） |
| Rabbit anti-TRPC6 | Proteintech, Wuhan, China | 00026504 | 1:1000 (WB)  1:200 (IF) |
| Rabbit anti-TRPC3 | Alomone Labs, Jerusalem, Israel | ACC-016 | 1:200（TEM） |
| Rabbit anti-TRPC6 | Alomone Labs, Jerusalem, Israel | ACC-017 | 1:200 (TEM) |
| Rabbit anti- BDNF | Signalway Antibody, Maryland, US | 32263 | 1:1000 (WB) |
| Mouse anti-P2X3 | SANTA CRUZ, Dallas, TX, USA | sc-390572 | 1:200 (IF) |
| Mouse anti-β-actin | Proteintech, Wuhan, China | 66009-1-AP | 1:4000 (WB) |
| Mouse anti-GAPDH | Proteintech, Wuhan, China | 66004-1-lg | 1:4000 (WB) |
| Rabbit anti-PSD95 | Abcam, Cambridgeshire, UK | ab18258 | 1:1000 (IF) |
| Rabbit anti- Synaptophysin | Abcam, Cambridgeshire, UK | ab32127 | 1:200 (IF) |
| Biotinylated griffonia simplicifolia lectin I, Isolectin B4 | Vector, California, USA | B-1205 | 1:200 (IF) |
| Goat anti-CGRP | Abcam, Cambridgeshire, UK | ab36001 | 1:200 (IF)  1:500 (TEM) |
| Mouse anti-NF200 | Sigma-Aldrich, St. Louis, MO, USA | N2912 | 1:200 (IF) |
| Neurobiotin | Vector, California, USA | SP-1120 | 1% |
| Anti-rabbit lgG,HRP-linked Antibody | Cell Signaling Technology, Boston, MA, USA | 7074 | 1:4000 (WB) |
| Anti-mouse lgG,HRP-linked Antibody | Cell Signaling Technology, Boston, MA, USA | 7076 | 1:4000 (WB) |
| Donkey anti-Goat IgG (H+L) Highly Cross-Adsorbed Secondary Antibody, Alexa Fluor 488 | Invitrogen, Carlsbad, CA, USA | A21202 | 1:800 (IF)  1:200 (TEM) |
| Donkey anti-Rabbit IgG (H+L) Cross-Adsorbed Secondary Antibody, Alexa Fluor 594 | Invitrogen, Carlsbad, CA, USA | A-1207 | 1:800 (IF) |
| Donkey anti-Goat IgG (H+L) Cross-Adsorbed Secondary Antibody, Alexa Fluor 594 | Invitrogen, Carlsbad, CA, USA | A11058 | 1:800 (IF) |
| Donkey anti-Mouse IgG (H+L) Cross-Adsorbed Secondary Antibody, Alexa Fluor 594 | Invitrogen, Carlsbad, CA, USA | A21203 | 1:800 (IF) |
| Donkey anti-Mouse IgG (H+L) Cross-Adsorbed Secondary Antibody, Alexa Fluor 647 | Jackson, Carlsbad, CA, USA | 711-605-151 | 1:800 (IF) |
| Bradykinin ELISA kit | Abcam | ab136936 |  |
| RNAscope Probe-Mm-TRPC3 | Advanced Cell Diagnostics, ACD, California, USA | 525201 |  |
| RNAscope Probe-Mm-TRPC6-c2 | Advanced Cell Diagnostics, ACD, California, USA | 442951-c2 |  |
| Chemicals, Peptides and Recombinant Proteins | | | |
| Strychnine | Sigma-Aldrich, St. Louis, MO, USA | S8753 |  |
| Gabazine | Sigma-Aldrich, St. Louis, MO, USA | S106 |  |
| Tetrodotoxin (TTX) | TOCRIS, Bristol, UK | 43F |  |
| Formalin | Sigma-Aldrich, St. Louis, MO, USA | HT5012 |  |
| collagenase | Sigma-Aldrich, St. Louis, MO, USA | C0130 |  |
| trypsin | Sigma-Aldrich, St. Louis, MO, USA | T2600000 |  |
| QX314 | Sigma-Aldrich, St. Louis, MO, USA | L5783 |  |
| ketamine | Sigma-Aldrich, St. Louis, MO, USA | K-002 |  |
| Capsaicin | TOCRIS, Bristol, UK | 0462 |  |
| BDNF | PeproTech, Cranbury, NJ, USA | 450-10 |  |
| DiI | Molecular Probe, NJ, USA | D3911 |  |
| SAR7334 | MedChemExpress LLC, NJ, USA | HY-15699A |  |
| Bradykinin | Abcam, Cambridgeshire, UK | ab120470 |  |
| Icatibant | MedChemExpress LLC, NJ, USA | HY-17446 |  |
| SSR 240612 | BIOFOUND, Shanghai, China | YzM003514 |  |
| Complete Freund’s adjuvant | Sigma-Aldrich, St. Louis, MO, USA | F5881 |  |
| Virus Strains | | | |
| rAAV-U6-Loxp-CMV-mCherry-SV40 pA-Loxp-shRNA1(TRPC3) | BrainVTA, Wuhan, China | PT-2900 |  |
| rAAV-U6-Loxp-CMV-mCherry-SV40 pA-Loxp-shRNA3(TRPC6) | BrainVTA, Wuhan, China | PT-2901 |  |
| rAAV-EF1a-DIO-BDNF-Flag-pHluorin-WPRE-bGH pA | BrainVTA, Wuhan, China | PT-0395 |  |
| rAAV-U6-Loxp-CMV-EGFP-SV40 pA-Loxp-shRNA1(BDNF) | BrainVTA, Wuhan, China | PT-0521 |  |
| rAAV-U6-Loxp-CMV-mCherry-SV40 pA-Loxp-shRNA(scramble) | BrainVTA, Wuhan, China | PT-0967 |  |
| rAAV-U6-Loxp-CMV-EGFP-SV40 pA-Loxp-shRNA(scramble) | BrainVTA, Wuhan, China | PT-0552 |  |
| rAAV2/9-CaMKIIa-GCaMP6s-WPRE-pA | BrainVTA, Wuhan, China | PT-0110 |  |
| Experimental models: Organisms/Stains | | | |
| Mouse: TRPC3 KO | Dr. Lutz Birnbaumer  Dr. Marc Freichel | N./A |  |
| Mouse: TRPC6 KO | Dr. Lutz Birnbaumer  Dr. Marc Freichel | N./A |  |
| Software | | | |
| Clampex 9.2 | Axon Instrument | N/A |  |
| Clampfit 10.6 | Axon Instrument | N/A |  |
| Olympus Fluoview version 3.1 | Olympus | N/A |  |
| Other | | | |
| Microliter syringe pump | KD Scientific | LEGATO 130 |  |
| FV1000 laser-scanning confocal microscope | Olympus | N/A |  |
| FV3000 laser-scanning confocal microscope | Olympus | N/A |  |
| Model 400 heated base  Series 8 model 390G | IITC Life Science | Model 400  Model 390G |  |
| von Frey Hairs | Bioseb | N/A |  |
| MultiClamp 700B | Axon Instrument | N/A |  |
| Primers | | | |
| TRPC3 primer 1 C3LoxF | GCTATGATTAATAGCTCATACCAAGAGATC |  |  |
| TRPC3 primer 2 C3LoxR | GGTGGAGGTAACACACAGCTAAGCC |  |  |
| TRPC3 primer 3 C3LoxF2 | GAATCCACCTGCTTACAACCATGTG |  |  |
| TRPC3 primer 4 C3LoxR | GGTGGAGGTAACACACAGCTAAGCC |  |  |
| TRPC6 primer 1 TRPC6_01 | ACGAGACTAGTGAGACGTGCTACTTCC |  |  |
| TRPC6 primer 2 TRPC6_02 | GGGTTTAATGTCTGTATCACTAAAGCCTCC |  |  |
| TRPC6 primer 3 TRPC6_03 | CAGATCATCTCTGAAGGTCTTTATGC |  |  |
| TRPC6 primer 4 TRPC6_04 | TGTGAATGCTTCATTCTGTTTTGCGCC |  |  |
